# Identification of a distinct cluster of LY6E^+^ macrophages in esophageal squamous cell carcinoma: functional phenotype, spatial interaction, and prognostic significance

**DOI:** 10.1038/s41416-026-03456-4

**Published:** 2026-04-29

**Authors:** Kun Chen, Jianjiao Ni, Yida Li, Sijia Gu, Ye Li, Xi Yang, Shyamal Goswami, Qiyu Luo, Yalei Zhang, Ling Qian, Yudi Hu, Runye Zhou, Yating Wang, Jingjing Liu, Xiuyu Cai, Chunrong Zhang, Saifullah Afridi, Yi Chen, Peng Wang, Zhengfei Zhu

**Affiliations:** 1 Department of Radiation Oncology, Fudan University Shanghai Cancer Center, Shanghai, China; 2 Department of Oncology, Shanghai Medical College, Fudan University, Shanghai, China; 3 Shanghai Clinical Research Center for Radiation Oncology, Shanghai Key Laboratory of Radiation Oncology, Shanghai, China; 4 The Affiliated Suzhou Hospital of Nanjing Medical University, Suzhou Municipal Hospital, Gusu School, Nanjing Medical University, Suzhou, Jiangsu China; 5 Department of Hepatic Oncology, Zhongshan Hospital, Fudan University, Shanghai, China; 6 Liver Cancer Institute, Zhongshan Hospital, Key Laboratory of Carcinogenesis and Cancer Invasion (Ministry of Education), Fudan University, Shanghai, China; 7 Research Center of Clinical Medicine, Affiliated Hospital of Nantong University, Nantong, China; 8 Department of Physiology and Medicine, Shantiniketan Medical College, Birbhum, India; 9 Key Laboratory of Immune Response and Immunotherapy, Shanghai Institute of Immunity and Infection, Chinese Academy of Sciences, Shanghai, China; 10 Department of VIP Inpatient, Sun Yet-Sen University Cancer Center, Guangdong Provincial Clinical Research Center for Cancer, State Key Laboratory of Oncology in South China, Collaborative Innovation Center for Cancer Medicine, Guangzhou, China; 11 Department of Thoracic Surgery, Nantong Tumor Hospital, Jiangsu, China; 12 Molecular Oncology Laboratory, Vaccine and Drug Development Biotechnology Research Group, VPCLS, TUBİTAK Marmara Research Center, Kocaeli, Istanbul, Turkey; 13 Department of Clinical Laboratory Shanghai East Hospital, Tongji University, Shanghai, China

**Keywords:** Cancer microenvironment, Oesophageal cancer, Monocytes and macrophages

## Abstract

**Background:**

Tumor-associated macrophages are key players in cancer progression, but their heterogeneity is not fully understood. This study aims to investigate the role of a specific macrophage subpopulation marked by LY6E in esophageal squamous cell carcinoma (ESCC).

**Methods:**

We analyzed macrophage subsets in ESCC, focusing on LY6E^+^ populations. Their gene expression signature, functional characteristics, and correlation with patient prognosis and immunotherapy response were assessed through single cell RNA sequencing and multiplex immunohistochemistry.

**Results:**

A distinct LY6E^+^ macrophage subpopulation was identified, enriched in tumors and associated with poor prognosis. These cells exhibited an M2-like, immunosuppressive phenotype, yet possessed high phagocytic and antigen-presenting potential. A potential niche involving LY6E^+^ macrophages, exhausted CD8^+^ T cells, and ICAM2^+^ tumor cells was discovered, which predicted a better response to immunotherapy.

**Conclusions:**

Our findings reveal a critical and complex role for LY6E^+^ macrophages in ESCC, highlighting them as a potential target for therapeutic intervention.

## Background

Esophageal squamous cell carcinoma is the predominant histological subtype of esophageal cancers with relatively high incidence in Central Asia, Eastern, and Southern Africa [1]. With the lack of early detection and efficient treatment strategies, the 5-years survival rate was only 20–30% [2]. With the recent development of immunotherapy, emerging immune checkpoint blockade therapies have been applied in ESCC [3]. However, a considerable group of patients failed to benefit from such therapy. It is urgent to conduct a mapping of tumor-immune contexture to uncover the mechanisms of therapeutic response and resistance.

Macrophages are one of the most abundant and heterogeneous cell populations in the tumor microenvironment (TME) [4]. Tumor-associated macrophages (TAMs) are generally thought to be immunosuppressive and to promote tumor progression and therapy resistance *via* crosstalk with tumor and immune cells, and *via* remodeling of the extracellular matrix [5, 6]. However, TAMs are also the main source of the chemokine CXCL9, which recruits CD8^+^ T cells to the tumor and sensitizes tumor cells to immunotherapy [7, 8]. These observations suggest that TAMs have more functionally diverse pro- and anti-tumor phenotypes. Conventionally, exposure of macrophages to type 1 or type 2 cytokines induces their polarization into phenotypic and functionally distinct subpopulations, which include the classically activated M1 macrophages and alternatively activated M2 macrophages [9]. However, recent multi-omics studies have revealed an even greater diversity of macrophages subsets that defy traditional classification systems.

Single-cell RNA sequencing (scRNA-seq) has been widely used to elucidate the diversity of macrophages in various cancer types [10], and the results have enabled a more precise classification of TAMs phenotypes and functions. For example, the presence of tissue-resident folate receptor beta (FOLR2)^+^ macrophages in breast cancer was reported to correlate positively with the number of CD8^+^ T cells and with a favorable prognosis [11]. In contrast, secreted phosphoprotein 1 (SPP1)^+^ macrophages were associated with an immunosuppressive and tumor-promoting phenotype in multiple cancer types, and their presence correlated with both an unfavorable prognosis and poor response to immunotherapy [12, 13]. Additional TAMs populations expressing high levels of genes associated with the inflammatory response, angiogenesis, and interferon response have also been identified in the TME of various cancers [10]. In ESCC, the diversity of macrophages has also been elucidated by scRNA-seq. For example, 4 macrophages subclusters were identified as Macro-C1-IL6, Macro-C2-IL1RN, Macro-C3-CSF1, and Macro-C4-LILRB2 [14]. Besides, another study also separated macrophages into 4 subpopulations as Macro-C1, Macro-C2, Macro-C3, and Macro-C4 [15]. However, the functional phenotype and prognostic significance of the specific macrophages in ESCC have not been fully elucidated.

In this study, we explored the heterogeneity of TAMs in human ESCC using scRNA-seq and identified a novel population of LY6E^+^ macrophages. These cells possessed an unusual functional phenotype in that they were not only immunosuppressive but also exhibited strong antigen-presenting and phagocytic capabilities. Further investigation, including comprehensive cellular analysis of the spatial interactions between LY6E^+^ macrophages and other cell types within the TME, revealed a potential niche formed by LY6E^+^ macrophages, exhausted CD8^+^ T cells, and ICAM2^+^ tumor cells. This niche was correlated with a superior response to immunotherapy.

## Methods

### Human sample collection

This study analyzed samples from 3 cohorts of ESCC patients. Samples of Cohort 1 were used for scRNA-seq and obtained from 18 treatment-naïve, histologically confirmed ESCC patients who underwent surgical resection at Fudan University Shanghai Cancer Center in 2021. Samples of Cohort 2 were used for multiplexed immunohistochemistry and obtained from 249 treatment-naïve, histologically confirmed ESCC patients who underwent primary surgical resection at Fudan University Shanghai Cancer Center, Sun Yet-Sen University Cancer Center, or Nantong Tumor Hospital in 2013. Samples of Cohort 3 were used for validating the predictive role of LY6E^+^ macrophages in patients receiving immune checkpoint blockade (ICB) therapy and obtained from 22 patients who received neoadjuvant immunotherapy followed by surgical resection at Fudan University Shanghai Cancer Center in 2021–2023. Informed consents were obtained from the patients in the 3 cohorts, and this study was approved by the Ethics Committee of Fudan University Shanghai Cancer Center and the other participating centers (approved number: 050432-4-2307E).

### Tissue preparation for scRNA-seq

Tumor tissues and adjacent normal tissues were collected and immediately placed on ice in MACS tissue storage solution from Miltenyi Biotech (RRID:SCR_008984, 130-100-008). Tissues were minced into 1 mm^3^ pieces and processed to a single-cell suspension using a gentleMACS Dissociator (RRID:SCR_025922) with a digestion cocktail composed of RPMI 1640 medium supplemented with collagenase type IV (0.5 mg/ml; C5138) and DNase I solution (0.1 mg/ml; DN25) from Sigma-Aldrich (RRID:SCR_008988). After filtering (70 μm mesh; BD Biosciences) and incubation with red blood cell lysis buffer for 5 min on ice (RRID:SCR_008984, 130-094-183), the single-cell suspension was analyzed by droplet-based scRNA-seq.

### scRNA-seq data processing

Cell Ranger software (RRID:SCR_017344) was used to align the reads to the reference GRCh38 human genome with the default parameters. The generated raw gene expression metrics for each sample were further analyzed using the Seurat package of R (RRID:SCR_007322). For quality control, samples with red blood cell genes or mitochondrial RNA content >20% were filtered out. Highly variable genes were selected with the FindVariableGenes function and processed with the Normalization and ScaleData functions. The scale data were then subjected to principal component analysis with RunPCA and the top 30 principal components were used to perform unsupervised clustering with FindClusters. Marker genes of specific clusters were identified with the FindAllMarkers function and clusters were annotated with the mark genes. For analysis of scRNA-seq data downloaded from the Gene Expression Omnibus (RRID:SCR_005012), the procedure for processing of the raw gene expression metrics was as described above. Subsequently, myeloid cells were defined, extracted, and merged for further analysis. Merging was performed by running a harmony analysis for batch correction using the Runharmony function with the harmony package (RRID:SCR_022206). Finally, the clustering results were visualized using UMAP plots generated with the RunUMAP function.

### TCR sequencing data processing

TCR sequences from the scRNA-seq analysis were processed using the cellranger vdj pipeline (RRID:SCR_023221) and integrated with T cell clusters using the immunarch package (RRID:SCR_023089). Clonotype analysis was performed with scRepertoire (RRID:SCR_025691) and expanded cells were defined as those with a clone size of ≥3 cells. The clonality of exhausted CD8^+^ T cells was determined using the STARTRAC package (version 0.1.0).

### Cell–cell interaction analysis

Cell–cell interactions were analyzed using the CellChat repository (Version 1.0.0). Based on the random permutations of cells, the mean of the average receptor–ligand pair expression and the *p*-value for the probability of a specific cell type in the interacting pair were calculated. To systematically identify ligand-receptor interactions that may drive transcriptomic alterations in the cell populations of interest, we employed NicheNet using normalized SCT expression matrices. For background gene expression, we retained genes detected in at least 0.1% of target cells. Differential gene sets for NicheNet (version 1.3.1) analysis were defined as follows: (1) tumor versus normal tissues and (2) specific cell subsets versus other related cell populations, applying thresholds of log₂ fold change >0.25 and adjusted *P* value < 0.05. Top candidate ligands were prioritized based on Pearson correlation analyses, which quantified the predictive capacity of sender cell-derived ligands for expression changes in receiver cells.

### Analysis of gene set signature scores

Gene set signature scores were calculated from the scRNA-seq data with the AddModuleScore function of the Seurat package (version 4.4.0). M1-like, M2-like, angiogenesis, phagosome, antigen-presenting, immunoinhibitory gene expression signatures, progenitor exhausted CD8^+^ T cell signature, and terminal exhausted CD8^+^ T cell signature [16] (referred to as ‘feature gene sets’) are shown in Supplementary Table 3. After calculating the scores, each cell was assigned a corresponding gene set score. For the bulk RNA-seq data, gene set variation analysis (GSVA, RRID:SCR_021058) was used to calculate the cell-specific gene signature score to represent the abundance of a specific cell type. The LY6E^+^ macrophages and niche gene signatures are shown in Supplementary Table 3.

### Multiplexed immunohistochemistry and spatial analysis

Tumor microarray slides containing formalin-fixed paraffin-embedded tissues were warmed at 58 °C and then deparaffinized and dewaxed by serial passage through three changes of xylene for 10 min each, two changes of 100% ethanol for ~5 min each, and 95%, 80%, and 70% alcohol for 3 min each. Tumor microarray slides were briefly fixed in 10% neutral-buffered formalin and antigen retrieval was achieved by heating in preheated AR6 buffer (pH 6.0, PerkinElmer) in a pressure cooker for 10 min. After cooling, the slides were washed three times for 5 min each in 0.03% Tris-buffered saline with Tween-20 (Amresco) with gentle agitation. Tissues were then incubated for 10 min with freshly prepared 3% H_2_O_2_ to quench endogenous peroxidase activity, rinsed again, and incubated with 10% normal goat serum (S-1000, Vector Labs) for 20 min at room temperature. Sections were then incubated with primary Abs against human ICAM2 (RRID:AB_2798188), cytokeratin (Abcam, ab80826), CD68 (RRID: AB_2721140), PD1 (CST, 86163), CD8 (RRID: AB_2882205), and LY6E (Abcam, ab300399) or SPP1 (Abcam, ab214050), CD68 (RRID: AB_2721140), and SIRPα (Origene, TA381503) and then incubated with the corresponding isotype-matched polymer-based horseradish peroxidase-conjugated secondary Abs (Vector Labs) for 20 min at room temperature. After washing the slides, multiplexed antigen detection was achieved using a multi-fluorophore kit (Perkin Elmer, NEL810001KT), as specified by the manufacturer. Finally, the tissues were counterstained with 4′,6-diamidino-2-phenylindole (DAPI; Sigma-Aldrich, D9542), mounted in Vectashield Hardset**®** fluorescence mounting medium (Vector Labs), dried in the dark for 30 min, and imaged using a Vectra 3.0 Multispectral Imaging System (RRID:SCR_025828). Image analysis was performed using inForm (RRID:SCR_019155).

To analyze the distance between specific cell types in the ESCC tumor microarrays described above, we enumerated the target phenotype and coordinates of each cell type of interest. For the spatial distribution between LY6E^+^ CD68^+^ cells and ICAM2^+^ CK^+^ cells or PD1^+^ CD8^+^ cells, 39 patients were selected. The distance between any ICAM2^+^ CK^+^ cells or PD1^+^ CD8^+^ cells and the closest LY6E^+^ or LY6E^-^ macrophages was recorded by Qupath (RRID:SCR_018257).

### Statistical analysis

Unless noted, the median value or best cut-off point determined by the “surv_cutpoint” function of the survminer package (RRID:SCR_021094) was used to dichotomize groups based on multiplexed immunohistochemistry markers or GSVA scores for survival analysis or response rate. Kaplan–Meier survival curves were generated and compared using the log-rank test to assess differences in survival between groups. The correlation analysis between immune cells was determined using Pearson correlation analysis. Statistical analysis was performed with Prism 9.0 software (RRID:SCR_002798). Mann–Whitney U test was used to compare two groups. A *p*-value of <0.05 was considered statistically significant.

## Results

### Generation of the ESCC single-cell transcriptomic atlas

**Fig. 1:**
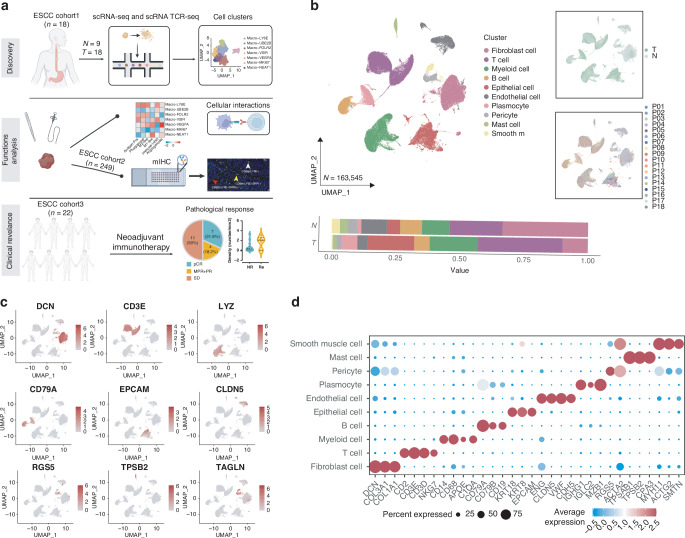
Generation of the ESCC single-cell transcriptomic atlas. **a** Schematic overview of the experimental design. **b** UMAP plot of various cell subpopulations in 27 ESCC tissue samples identified by scRNA-seq. Lower panels show the proportions of each subpopulation in tumor (T) and adjacent normal (N) esophageal tissues, right panels show UMAP plot containing various tissues or samples. **c** UMAP plots showing marker gene expression of the cell subclusters. **d** Dotplot of the top differentially expressed genes in the indicated cell subpopulations.

To characterize the TME in ESCC, we performed scRNA-seq of 18 ESCC tumor tissues and 9 adjacent normal tissues (>3 cm apart) collected from our 18-patients cohort (Fig. 1a and Supplementary Table 1). After filtering to exclude dead cells and doublets, scRNA-seq data from a total of 163,545 live single cells were analyzed. UMAP plots were constructed of the cell clusters annotated with marker genes and the cells were classified into 10 clusters: fibroblasts (*DCN*, *COL3A1*, and *COL1A1* positive), T cells (*CD2*, *CD3D*, and *CD3E* positive), myeloid cells (*CD68*, *CD14*, *APOE*, and *C1QA* positive), B cells (*CD19*, *CD79A*, and *CD79B* positive), epithelial cells (*EPCAM*, *KRT8*, and *KRT18* positive), endothelial cells (*VWF*, *CDH5*, and *CLDN5* positive), plasmocytes (*MZB1*, *IGHG1*, and *IGLC2* positive), pericytes (*RGS5* and *ACTA2* positive), mast cells (*TPSAB1*, *TPSB2*, *CPA3*, and *CTSG* positive), and smooth muscle cells (*MYH11*, *ACTG2*, and *SMTN* positive) (Fig. 1b–d).

### Identification of LY6E^+^ macrophages in human ESCC

**Fig. 2:**
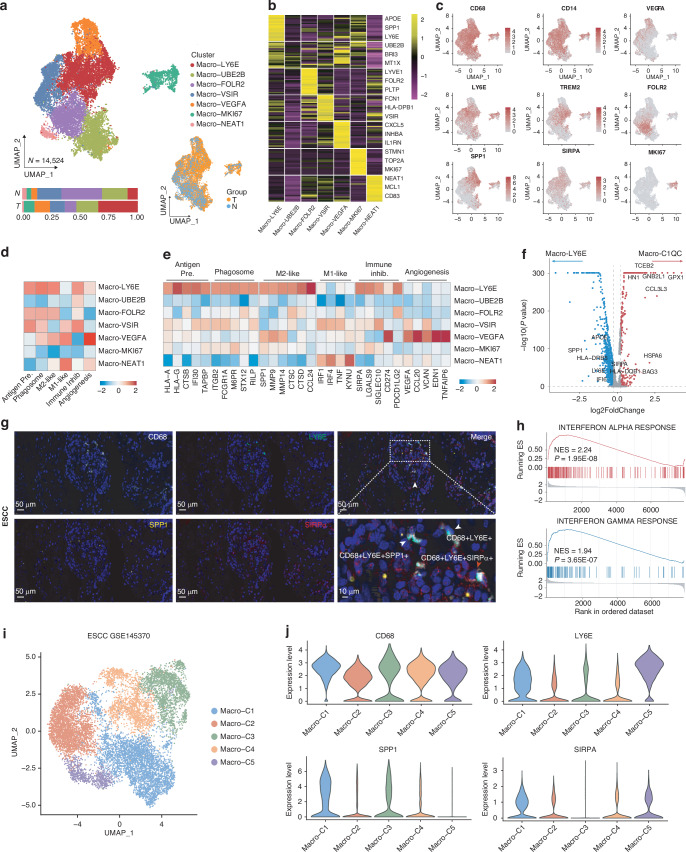
Identification of LY6E^+^ macrophages in human ESCC. **a** UMAP plot of various macrophages subpopulations. Lower panels show the proportions of each subpopulation in tumor and adjacent normal esophageal tissues. **b** Heatmap of the top differentially expressed genes in the indicated macrophages subpopulations. **c** UMAP plots showing marker gene expression of the macrophages subclusters. **d** Heatmap of the module scores of functional gene sets in the macrophage subpopulations. **e** Heatmap of the expression profiles of the feature functional gene sets in the macrophage subpopulations. **f** Volcano plot showing the differentially expressed genes in the Macro-LY6E cluster identified in this study and the Macro-C1QC cluster identified in the analysis of ESCC by Zhang et al. **g** Representative images of in situ immunofluorescent staining of ESCC samples for CD68, LY6E, SPP1, and SIRPα (*n* = 40). Scale bars, 10 μm. **h** GSEA showing significantly up-regulated pathways in Macro-LY6E compared with the findings in other macrophage subclusters. **i** UMAP plot of various macrophages subpopulations in ESCC datasets from GSE145370. **j** Vlnplot of the feature genes by the macrophages subpopulations in the ESCC dataset from GSE145370.

The heterogeneity of TAMs plays a vital role in tumor progression and treatment response. It is essential that TAMs subpopulations are carefully identified and characterized. Herein, a total of 14,524 macrophages were identified and further divided into 7 subclusters. All the clusters were detected in all ESCC tumor and normal tissue samples, regardless of the collection location (Fig. 2a). The Macro-LY6E subset expressed high levels of *LY6E*, *APOE*, and *SPP1*, and was additionally characterized by relatively high expression of gene signatures associated with immune inhibition, antigen presentation, and phagocytosis (Fig. 2b). The Macro-FOLR2 cluster expressed high levels of *FOLR2*, *PLTP*, and *LYVE1* and was located mainly in para-cancerous tissue, indicating that the cells were tissue-resident macrophages [10]. Macro-MKI67 cluster was marked by high expression of proliferation-related genes, such as *MKI67* and *STMN1*. Macro-VEGFA cells were enriched for expression of angiogenesis-related genes, such as *VEGFA*, and expressed the highest levels of those genes among the macrophage subclusters identified. Macro-VSIR cells were marked by high expression of *VSIR* and a strong immunoinhibitory profile consistent with the observed role of these cells in pancreatic cancer [17]. Macro-NEAT1 cells expressed high levels of *NEAT1*, *CD83*, and *MCL1* and possessed the highest M1-like gene signature score, indicating a proinflammatory role. Finally, Macro-UBE2B cells expressed high levels of mitochondria-associated genes such as *MT1X*, but the role of this subcluster remains to be further elucidated (Fig. 2b, c and Supplementary Table 2).

The Macro-LY6E cluster was the main macrophage subset located in ESCC tumor tissues (Supplementary Fig. 1a), and the gene signature was associated with not only relatively high M2-like and immunosuppressive activity but also high antigen-presenting and phagocytic potential, which are generally associated with anti-tumor activity (Fig. 2d, e). It is worth noting that Macro-LY6E clusters and Macro-MKI67 showed considerable overlap in their gene expression profiles. We then further compared the differentially expressed genes and the functional signatures between the two groups, The results indicated that, compared to the Macro-MKI67 cluster, the Macro-LY6E cluster exhibited elevated signatures associated with M2-like polarization, immune inhibition, phagocytosis, and antigen presentation (Supplementary Fig. 1b, c). The further cluster similarity analysis showed that the Macro-LY6E cluster was highly similar to the C1QC^+^ TAMs in the ESCC myeloid cell cluster from pan-cancer datasets (Supplementary Fig. 1d) [18]. However, the Macro-LY6E cluster identified here showed higher expression of *SPP1*, *LY6E*, *SIRPA*, *IFI6*, and *HLA-DQB* than C1QC^+^ TAMs, suggesting that these are distinct macrophage subpopulations (Fig. 2f). Besides, TREM2^+^/SPP1^+^macrophages have been well-characterized across multiple cancer types, including ESCC [12, 13]. To further highlight distinct features between Macro-LY6E and TREM2^+^/SPP1^+^ subsets, TREM2^+^/ SPP1^+^ macrophages derived from the GSE160269 ESCC dataset were compared with Macro-LY6E (Supplementary Fig. 1e, f) [15]. The Macro-LY6E cluster identified in this study exhibited higher expression levels of *LY6E, CXCL9, IFI16*, and *HLA-DRB1* (Supplementary Fig. 1g). Furthermore, we compared functional signatures between the Macro-LY6E and TREM2^+^/SPP1^+^TAMs subsets. The analysis revealed that, compared to the TREM2^+^/SPP1^+^TAMs subset, the Macro-LY6E cluster displayed elevated signatures related to immunosuppression and antigen presentation, while the TREM2^+^/SPP1^+^TAMs subset exhibited stronger associations with M2 polarization and phagocytosis. (Supplementary Fig. 1h). To confirm the existence of LY6E^+^ macrophages in ESCC, we performed in situ multi-color immunofluorescence staining of tissue sections for their representative or functional markers including *CD68*, *LY6E*, *SIRPA*, and *SPP1*. Indeed, LY6E^+^ macrophages co-localized with SPP1^+^ and SIRPα^+^ cells in ESCC tissues (Fig. 2g). When investigating the enrichment pathway in LY6E^+^ macrophages by GSEA, interferon response pathways were significantly up-regulated in LY6E^+^ macrophages (Fig. 2h). To further validate the presence of LY6E^+^ macrophages, we collected and reanalyzed the macrophages from another ESCC datasets consisting of 7 patients with normal and tumor tissues, the macrophages were divided into 5 clusters (Fig. 2i and Supplementary Fig. 1i–l). Remarkably, Macro-C1 selectively expresses the characteristic genes of LY6E^+^ macrophages and is characterized by relatively high M2-like and immunosuppressive activity, as well as strong antigen-presenting and phagocytic potential, mirroring the traits of LY6E^+^ macrophages (Fig. 2j and Supplementary Fig. 1m). Collectively, LY6E^+^ macrophages with distinct characteristics were identified in human ESCC.

### LY6E^+^ macrophages abundance correlates with poor prognosis but well response to ICB in ESCC

**Fig. 3:**
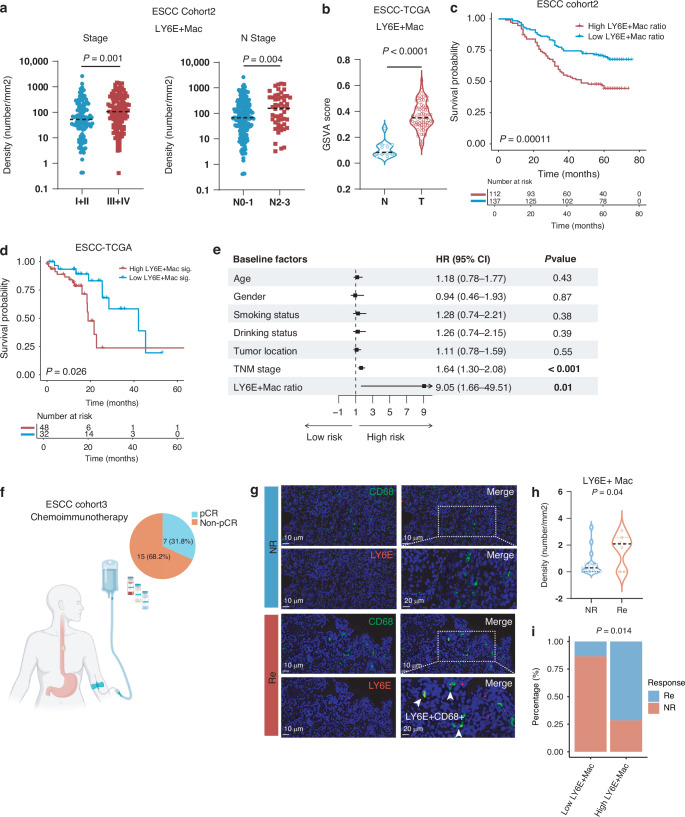
LY6E^+^ macrophages abundance correlates with poor prognosis but well response to ICB in ESCC. **a** LY6E^+^ macrophages density in ESCC tumors according to pathological stage (left) and node stage (right). *p*-value by Mann–Whitney test. **b** GSVA scores of Macro-LY6E in normal (N) and tumor (T) tissues from the TCGA ESCC dataset (*n* = 80). *p*-value by Mann–Whitney test. **c** Overall survival analysis of the ESCC validation cohort 2 (*n* = 249) stratified by the ratio of the Macro-LY6E. *p*-value was determined by the log-rank test. **d** Overall survival analysis of the TCGA ESCC cohort stratified by tumor expression of the Macro-LY6E signature (*n* = 80). *p*-value was determined by the log-rank test. **e** Forest plot depicting multivariate Cox regression analysis of clinical factors and LY6E^+^ macrophages in relation to overall survival in ESCC validation cohort 2. **f** Pathological evaluation of patients from the ESCC cohort 3 receiving neoadjuvant immunotherapy (*n* = 22). Detailed information is provided in Supplementary Table 1. **g** Representative images of in situ immunofluorescent staining of LY6E^+^ macrophages before immunotherapy in ESCC patients who reached pCR and non-pCR in ESCC cohort 3. **h** Density of Macro-LY6E in pre-treatment tumor samples of responding and non-responding ESCC patients receiving neoadjuvant immunotherapy (*n* = 22). *p*-value by Mann–Whitney test. **i** Correlation between immunotherapy response and the Macro-LY6E infiltration level in ESCC patients undergoing neoadjuvant anti-PD-1 therapy (*n* = 22). *p*-value by Fisher’s exact test.

To investigate the clinical significance of LY6E^+^ macrophages in ESCC, we conduct multiplexed immunohistochemistry regarding LY6E^+^ macrophages in our in-house ESCC cohort 2 (*n* = 249). The results showed the density of LY6E^+^ macrophages correlated positively with both tumor stage and node invasion (Fig. 3a). The GSVA score analysis demonstrated that LY6E^+^ macrophages were more enriched in ESCC tumor tissues than adjacent normal esophageal tissues (Fig. 3b). The overall survival analysis showed significant correlations between high expression of the LY6E^+^ macrophages gene signature and unfavorable prognosis, both in the TCGA ESCC cohort and our in-house ESCC cohort (Fig. 3c, d). Multivariate Cox regression (Fig. 3e) also identified LY6E^+^ macrophages to be an independent unfavorable factor for ESCC. Taken together, these results demonstrate that high infiltration of LY6E^+^ TAMs is an unfavorable prognostic biomarker in ESCC.

Subsequently, we investigated the relationship between response to ICB and the abundance of LY6E^+^ macrophages by performing in situ multi-color immunofluorescence staining of tissues from 22 ESCC patients who received neoadjuvant anti-PD-1 therapy (Fig. 3f). This analysis found a higher abundance of LY6E^+^ macrophages infiltration in the pre-treatment samples from responders than non-responders to neoadjuvant immunotherapy (Fig. 3g, h). Besides, an increased abundance of LY6E^+^macrophages correlates with an elevated response ratio in ESCC patients undergoing neoadjuvant anti-PD-1 therapy (Fig. 3i).

### High infiltration of LY6E^+^ macrophages is associated with exhausted CD8^+^ T cells

**Fig. 4:**
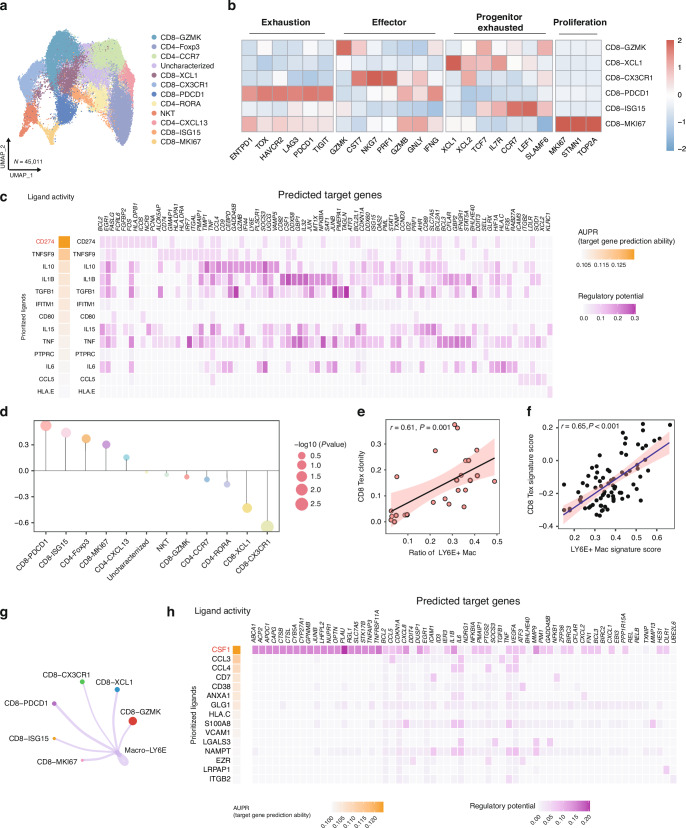
High infiltration of LY6E^+^ macrophages is associated with exhausted CD8^+^ T cells. **a** UMAP plot of various T cells subpopulations. **b** Heatmap of the expression profiles of the feature functional gene sets in the T cells subpopulations. **c** Heatmap showing the activity (left) and regulatory potential (right) of the ligands in LY6E^+^ macrophages cells driving the CD8^+^ T cells. **d** Relationship between the proportion of Macro-LY6E among total macrophages and the proportion of the indicated T cells subsets among total T cells. **e** Scatter plot showing the Pearson correlation between the proportion of Macro-LY6E among total macrophages and CD8-TER-EXH clonality in our ESCC scRNA-seq dataset (*n* = 27). **f** Pearson correlation between Macro-LY6E and CD8-TER-EXH signature scores in the TCGA ESCC cohort (*n* = 80). **g** The significant interaction numbers between Macro-LY6E and various CD8^+^ T cells subclusters. **h** Heatmap showing the activity (left) and regulatory potential (right) of the ligands in CD8-TER-EXH cells driving the phenotype of Macro-LY6E cells.

The predictive role of LY6E^+^ macrophages in ESCC patients receiving neoadjuvant immunotherapy indicated their involvement in adaptive immunity. Given the crucial role of macrophage-T cell interactions in immune response orchestration, we next focused on characterizing the interplay between LY6E^+^ macrophages and T cells. Using our ESCC scRNA-seq dataset, we conducted unsupervised clustering of T cells and identified 12 clusters: six CD8^+^ T cell, four CD4^+^ T cell, and one NKT cell cluster (Fig. 4a and Supplementary Fig. 2a–c). The CD8-GZMK cluster highly expressed *GZMK* and *CST7*. The CD8-PROG-EXH subset selectively expressed progenitor exhaustion markers, identifying it as progenitor exhausted CD8⁺ T cells (CD8^+^ Tpex)— a classification further supported by its elevated progenitor exhaustion signature score (Supplementary Fig. 2d). The CD8-CX3CR1 cluster was considered as terminal effector memory T cells (CD8^+^ Temra cells), due to its high expression of effector T cells markers. The CD8-TER-EXH cluster selectively expressed exhausted markers, such as *PDCD1*, *LAG3*, *HAVCR2*, and *TOX*, suggesting it to be terminal exhausted CD8^+^ T cells (CD8^+^ Tex) [19, 20]. This classification was further supported by elevated terminal-exhaustion signature scores in the CD8-TER-EXH subcluster (Supplementary Fig. 2d). CD8-ISG15 highly expressed the progenitor exhaustion markers. The CD8-PROLIF-EXH cluster highly expressed proliferating markers and terminal exhausted markers, such as *MKI67, STMN1, PDCD1*, *LAG3*, and *HAVCR2*, suggesting it to be proliferating exhausted CD8^+^ T cells [20] (Fig. 4b).

To elucidate the potential interactions between LY6E^+^ macrophages and CD8^+^ T cells, we first examined the regulatory effects of LY6E^+^ macrophages on CD8^+^ T cell populations. This analysis revealed significant enrichment of immunoinhibitory molecules in LY6E^+^ macrophages, particularly *CD274* (PD-L1), which exhibited potent immunosuppressive effects on CD8^+^ T cells (Fig. 4c). These findings were further supported by data from the GSE145370 dataset, where the homologous Macro-C1 population similarly mediated immunosuppression through CD274 (Supplementary Fig. 3a–d). Building on these observations, we subsequently investigated the correlation of LY6E^+^ macrophages with CD8^+^ T cells subclusters. Strikingly, LY6E^+^ macrophage abundance showed the strongest correlation with CD8^+^ Tex cells (Fig. 4d and Supplementary Fig. 2e). Single-cell sequencing of T cell receptors showed expansion (clone size ≥3) of several subsets, mainly CD8^+^ Tex cells and CD8^+^ effector memory T cells (Supplementary Fig. 2f, g). Therefore, we next analyzed the relationship between the expanded CD8^+^ Tex cells subset and LY6E^+^ macrophages in more detail. Evaluation of the clonality of CD8^+^ Tex cells with STARTRAC showed that CD8^+^ Tex clonality correlated positively with the relative frequency of LY6E^+^ macrophages in our ESCC dataset (r = 0.61, *p* = 0.001; Fig. 4e). The correlation between CD8^+^ Tex cells and LY6E^+^ macrophages was independently confirmed in TCGA ESCC datasets, showing strong positive correlation between the gene signature scores of LY6E^+^ macrophages and CD8^+^ Tex cells (r = 0.65, *p* < 0.001) (Fig. 4f). The GSE145370 dataset similarly demonstrated a positive relationship between Macro-C1 and CD8^+^ Tex cells (Supplementary Fig. 3e). These results demonstrated a positive correlation between LY6E^+^ macrophages and CD8^+^ Tex cells.

Additionally, LY6E^+^ macrophages exhibited preferential interactions with CD8^+^ Tex cells compared with other CD8^+^ T cells subpopulations (Fig. 4g). Our regulatory network analysis identified significant upregulation of macrophage-related cytokines (CSF-1, CCL3, CCL4) in CD8^+^ Tex cells, indicating their capacity to both recruit and phenotypically modulate LY6E^+^ macrophages (Fig. 4h). To further substantiate the specificity of this signaling axis, we then investigated the expression of CSF1R (receptor for CSF-1), CCR1 (receptor for CCL3), and CCR5 (receptor for CCL4) across the macrophage subclusters. As a result, *CSF1R* was highly expressed in the Macro-LY6E cluster, and *CCR1* showed the most pronounced upregulation in this population (Supplementary Fig. 2j). Cellular interactions between TAMs and exhausted CD8^+^ T cell subsets are pivotal in shaping the immunosuppressive tumor microenvironment. To investigate whether CD8^+^ Tpex cells exhibit similar interactions with Macro-LY6E, we then also compared the cellular communication between Macro-LY6E and CD8^+^ Tex cells versus CD8^+^ Tpex cells (Supplementary Fig. 2h, i). The results indicated that, in contrast to CD8^+^ Tpex cells, Macro-LY6E communicates with CD8^+^ Tex cells via inhibitory signaling axes such as LGALS9–HAVCR2, CD80/86–CTLA4, and NECTIN2–TIGIT. Furthermore, CD8^+^ Tex cells appear to recruit Macro-LY6E through ligands such as CCL3 and CSF1 [6]. In addition, in the GSE145370 dataset, the CD8^+^ Tex cells also showed both increased interactions with Macro-C1 and elevated expression of the same macrophage-attracting factors [6] (Supplementary Fig. 3f, g). These results suggest the specific role of the CD8^+^ Tex cells in attracting Macro-LY6E. Interestingly, the CD8^+^ Tex cells selectively exhibited high expression of both *CXCL13* and *ENTPD1* (Supplementary Fig. 2k), suggesting their potential tumor-reactive role [21, 22]. Correspondingly, the CD8^+^ Tex cells exhibited the most robust IFN-γ production among all T cell subsets (Fig. 4b), providing a mechanistic explanation for the observed interferon response pathway activation in LY6E^+^ macrophages. These results suggested the vital role of CD8^+^ Tex cells in attracting and polarizing LY6E^+^ macrophages.

### Formation of the possible niche of LY6E^+^ macrophages, CD8^+^ Tex cells and ICAM2^+^ tumor cells correlates with immunotherapy response

**Fig. 5:**
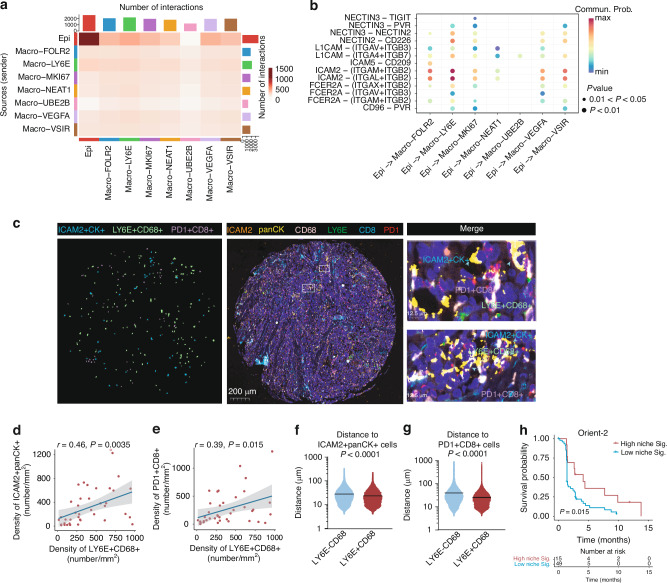
Formation of the possible niche of LY6E^+^ macrophages, CD8^+^ Tex cells and ICAM2^+^ tumor cells correlates with immunotherapy response. **a** Heatmap showing the significant interaction numbers between epithelial cells and various macrophages subclusters. **b** Summary of intercellular adhesion molecular interactions between epithelial cells and macrophages subpopulations. **c** Representative images of in situ immunofluorescent staining of CD68, LY6E, PD1, CD8, ICAM2, and panCK in ESCC samples (*n* = 39). Scale bars, 300 μm. **d** Scatter plot showing the Pearson correlation between the density of Macro-LY6E and ICAM2^+^ panCK^+^ cells in ESCC samples (*n* = 39). **e** Scatter plot showing the Pearson correlation between the density of Macro-LY6E and CD8^+^ Tex cells in ESCC samples (*n* = 39). **f** Distribution of distances between LY6E^+^ or LY6E^−^ macrophages and ICAM2^+^ panCK^+^ cells. *p*-value by Mann–Whitney test. **g** Distribution of distances between LY6E^+^ or LY6E^−^ macrophages and CD8^+^ Tex cells. *p*-value by Mann–Whitney test. **h** Progression-free survival of ESCC patients receiving Sintilimab in the ORIENT-2 studies stratified by low or high score of the niche gene signature (*n* = 64). *p*-value by log-rank test.

Considering the enrichment of LY6E^+^ macrophages in tumor tissues compared with the adjacent normal tissues, the tumor cells might play a vital role in attracting or retaining LY6E^+^ macrophages. Indeed, epithelial cells demonstrated the most frequent interactions with LY6E^+^ macrophages compared to other macrophage subpopulations (Fig. 5a). Further analysis revealed enhanced ICAM2-(ITGB2 + ITGAM/ITGAL)-mediated intercellular adhesion between epithelial cells and LY6E^+^ macrophages (Fig. 5b). Notably, in the GSE145370 dataset, Macro-C1 exhibited significantly elevated expression of ITGB2 and ITGAM relative to other macrophage subclusters (Supplementary Fig. 4a). These findings collectively suggest that tumor epithelial cells may preferentially retain LY6E^+^ macrophages through specific adhesion molecule interactions, particularly the ICAM2-(ITGB2 + ITGAM/ITGAL) axis.

Having characterized these functional interactions among LY6E^+^ macrophages, CD8^+^ Tex cells, and ICAM2^+^ tumor cells, we proceeded to investigate their spatial organization in human ESCC tissues by in situ multi-color immunofluorescence staining (Fig. 5c). Herein, the CD8^+^ Tex were determined by CD8^+^PD1^+^ T cells based on their selective and high-level expression of PDCD1 (Fig. 4b). Quantitative analysis revealed significant positive correlations between LY6E^+^ macrophages density with both PD1^+^CD8^+^ Tex cells and ICAM2^+^ tumor cells densities (Fig. 5d, e). Spatial mapping further demonstrated that LY6E^+^ macrophages were preferentially localized in closer proximity to both PD1^+^CD8^+^ Tex cells and ICAM2^+^ tumor cells compared to their LY6E^-^ counterparts (Fig. 5f, g). These results suggested that a functional niche comprising LY6E^+^ macrophages, CD8^+^ Tex cells, and ICAM2^+^ tumor cells within the TME.

**Fig. 6:**
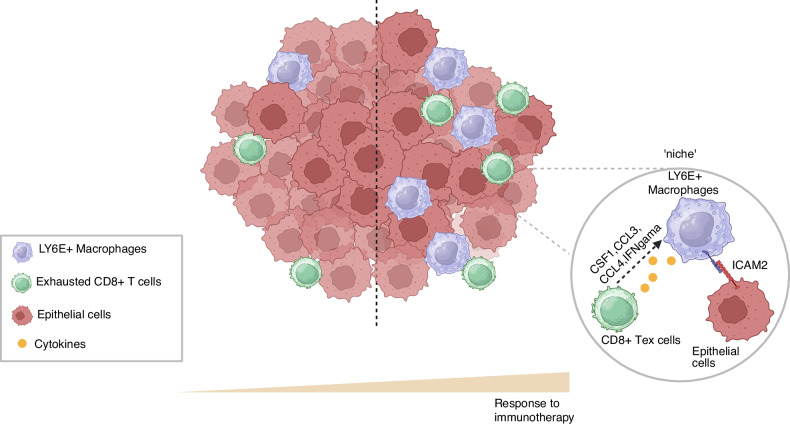
The niche formation by LY6E^+^ macrophages, CD8^+^ Tex cells, and ICAM2^+^ tumor cells within the TME of ESCC.

We next investigated the clinical implications of the existence of such a niche by constructing a combined ‘niche’ signature from the LY6E^+^ macrophage, CD8^+^ Tex cell, and ICAM2^+^ tumor cell gene signatures. The relationship between tumor expression of the niche signature and response to ICB treatment was then evaluated by comparing survival of cancer patients with high and low signature expression in various clinical studies. In the ESCC patients receiving Sintilimab (anti-PD-1 Ab) from the ORIENT-2 clinical trial [23], patients with high niche signature score correlated with longer progression-free survival (Fig. 5h). Besides, similar results were found in several other solid tumors in independent external datasets (Supplementary Fig. 4b–e) [24–26]. These results suggest the formation of a niche composed of LY6E^+^ macrophages, CD8^+^ Tex cells, and ICAM2^+^ tumor cells, which is associated with immunotherapy response (Fig. 6).

## Discussion

In this study, we identified a previously uncharacterized subset of intratumoral macrophages, LY6E^+^ macrophages, that possess M2-like and immunosuppressive activities but also exhibit antigen-presenting and phagocytic potential. LY6E^+^ macrophages were found to be enriched in tumor tissues and associated with an unfavorable prognosis in ESCC. The cellular interaction analysis showed that LY6E^+^ macrophages closely interacted with CD8^+^ Tex cells and ICAM2^+^ tumor cells. Moreover, spatial analysis of human ESCC tissues also showed the potential niche formation by LY6E^+^ macrophages, CD8^+^ Tex cells, and ICAM2^+^ tumor cells. Besides, the expression signature of this niche was found to be correlated with the response to ICB.

TAMs exhibit dualistic roles in ESCC progression and therapeutic response. On one hand, TAMs have been demonstrated to facilitate carcinogenesis and correlate with unfavorable clinical outcomes in ESCC. Pharmacological inhibition of the CCL2-CCR2 axis, by attenuating monocyte recruitment and subsequent TAMs accumulation, has shown potent anti-tumorigenic effects [27]. Conversely, CD68^+^HLA-DR^+^ M1-polarized macrophages have been identified as favorable prognostic indicators in ESCC [28]. The multifaceted role of TAMs makes their precise classification critically important. Recently, advances in multi-omics technologies have enabled the identification of distinct TAMs subpopulations in ESCC. For example, S100A9^+^ inflammatory macrophages localized in fibrotic niches have emerged as predictive biomarkers for treatment response in ESCC patients undergoing neoadjuvant chemo-immunotherapy [29]. APOC1^+^APOE^+^ macrophages were found to be accumulated in lymph node metastasis of ESCC patients, indicating their role in facilitating ESCC lymphatic dissemination [30]. Furthermore, Macro-SPP1, Macro-EREG, and Macro-STMN1 subpopulations show significant enrichment in non-responders versus responders following neoadjuvant immunochemotherapy for ESCC [31]. The characterization of these functionally diverse TAMs subsets paves the way for developing targeted macrophage-based therapeutic strategies.

Herein, through comprehensive scRNA-seq of ESCC, we report a previously uncharacterized subset of intratumoral macrophages, LY6E^+^ macrophages. Transcriptomic profiling revealed that LY6E^+^ macrophages exhibit molecular features overlapping with lipid-associated macrophages, including characteristic expression of SPP1, TREM2, FABP5, and MMP9 [32–34]. Notably, these macrophages simultaneously displayed significant enrichment of interferon signaling pathway genes, a hallmark feature of interferon-primed TAMs [35, 36]. Functionally, LY6E^+^ macrophages possess the immunosuppressive ability of lipid-associated macrophages and enhanced antigen-presenting and phagocytic abilities stimulated by interferon signaling [37]. This unique functional duality demonstrates the remarkable plasticity of TAMs subsets, suggesting that the distinctive LY6E^+^ macrophage phenotype emerges through coordinated interactions with tumor cells and CD8^+^ Tex cells-derived factors including IFN-γ and myeloid cells chemoattractants within the tumor niche.

Clinically, LY6E^+^ macrophages infiltration correlated with poor prognosis in ESCC, likely mediated through their potent immunosuppressive effects on CD8^+^ T cells. Paradoxically, elevated LY6E^+^ macrophages levels were associated with improved response to ICB therapy. In this study, we identified the PD-L1/PD-1 axis as the predominant immunosuppressive pathway mediating LY6E^+^ macrophages- CD8^+^ T cells interactions. PD-L1^+^ macrophages were thought to be an independent predictor of anti-PD-L1/PD-1 based immunotherapy in several studies [38, 39]. As a result, it may be the reason that LY6E^+^ macrophages possess the ability to predict immunotherapy response. Besides, CXCL13^+^CD8^+^T cells have been identified as a subset of tumor-reactive T cells and are associated with favorable responses to immune checkpoint blockade [21]. Additionally, *ENTPD1*, which encodes CD39, has also been recognized as a marker of tumor-reactive T cells [22]. In our data, CD8^+^ Tex cells selectively exhibited high expression of both *CXCL13* and *ENTPD1*, suggesting their potential tumor reactive role. The positive correlation between LY6E^+^ macrophages and tumor reactive CD8^+^ T cells might underpin the association of high LY6E^+^ macrophage abundance with ICB response.

Cellular interactions are critical for the phenotypic and functional regulation of immune cells. For example, in hepatocellular carcinoma, progenitor CD8^+^ T cells form a triad with CXCL13^+^ CD4^+^ T helper cells and dendritic cells, which regulate progenitor cell differentiation into PD-1^hi^ effector CD8^+^ T cells in response to PD-1 blockade [40]. In addition, tumor-specific CD8^+^ T cells in intratumoral immune triads interact with CD4^+^ T cells during the effector phase, thereby enhancing CD8^+^ T cells cytotoxicity and the elimination of tumor cells [41]. In ESCC, invasive epithelial cells collaborate with cancer-associated fibroblasts to create an immune-evasive niche that predicts clinical outcomes [42]. In this study, we discovered a novel potential niche within the TME comprising LY6E^+^ macrophages, CD8^+^ Tex cells, and ICAM2^+^ tumor cells that strongly correlates with ICB response. This niche appears to drive phenotypic reprogramming of LY6E^+^ macrophages and critically influences immunotherapeutic efficacy. This newly identified niche provides mechanistic insights into anti-tumor immunotherapy responses and offers potential predictive biomarkers for ICB efficacy.

In conclusion, we identified a unique intratumoral LY6E^+^ macrophages subset that is positively associated with response to ICB. Importantly, these LY6E^+^ macrophages form a spatially organized niche with CD8^+^ Tex cells and ICAM2^+^ tumor cells. These findings advance our understanding of how cellular interactions govern immune cell phenotypes and may guide personalized immunotherapy strategies. While these observations are compelling, the mechanistic underpinnings remain to be fully elucidated. Future studies employing co-culture systems with LY6E^+^ macrophages, naïve/activated T cells, and tumor cells will be essential to validate these cellular interactions. Additionally, the precise molecular mechanisms through which LY6E^+^ macrophages modulate immunotherapy responses warrant further investigation. Furtherly, although LY6E expression has been reported in monocytes [43, 44], the role of LY6E^+^monocytes within the tumor microenvironment remains poorly characterized. Here, we selected LY6E as the marker for the Macro-LY6E subcluster, in part because it is regulated by interferon—consistent with the activated interferon signaling pathway observed in Macro-LY6E compared with other macrophage clusters. However, as noted, whether LY6E acts as a functional mediator in Macro-LY6E macrophages and the underlying mechanisms involved remain to be fully elucidated. Further studies are therefore required to investigate the specific functions of LY6E in macrophages.

## Supplementary information

Supplementary information

Supplementary table1

Supplementary table2

Supplementary table3

## Data Availability

This study did not generate new unique reagents. ScRNA-sequencing data have been deposited at GSA (https://ngdc.cncb.ac.cn/gsa/) with the access number HRA008937 and are publicly available as of the date of publication.

## Code availability

The code utilized in this paper is available upon request. Further information and requests for resources connect with the Lead Contact, Zhengfei,Zhu (fuscczzf@163.com).
