# Supplementary material for: Identification of a distinct cluster of LY6E+ macrophages in esophageal squamous cell carcinoma: functional phenotype, spatial interaction, and prognostic significance
Source: Br J Cancer. 2026 Apr 29;135(3):382–93. doi: 10.1038/s41416-026-03456-4 (PMC13372808; doi:10.1038/s41416-026-03456-4)
Supplement: Supplementary file 1 — Supplementary information [file 41416_2026_3456_MOESM1_ESM.docx]

**Supplementary Figures**


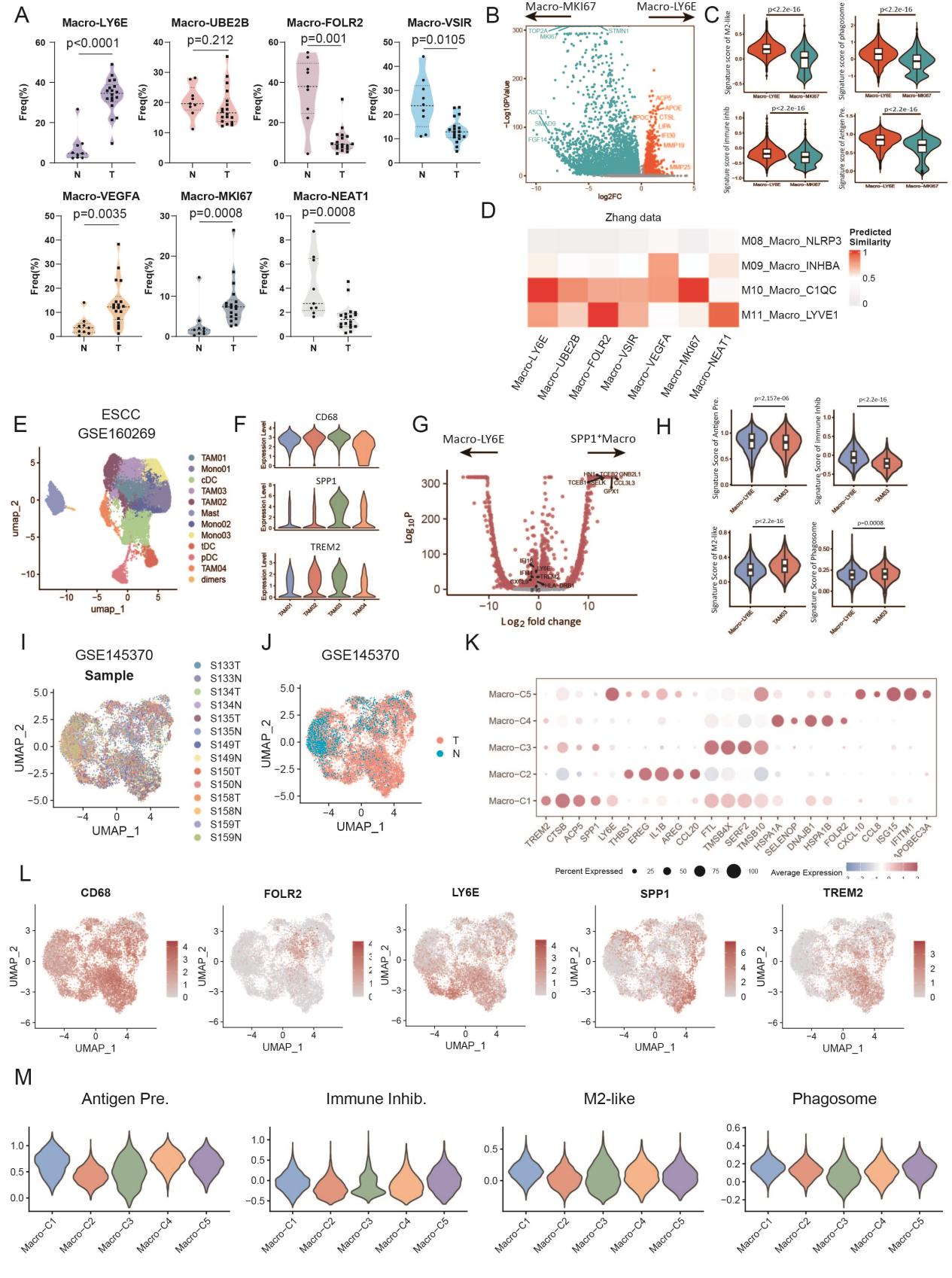


**Supplementary Fig 1. Identification of LY6E^+^ macrophages in human esophageal squamous cell carcinoma, related to Figure 2.**

(A)Frequencies of macrophage subpopulations relative to the macrophage in normal (N) and tumor (T) tissues.

(B)Volcano plot showing the differentially expressed genes in the Macro-LY6E cluster and Macro-MKI67 identified in this study.

1. Vlnplot of the scores of functional gene sets (antigen presentation ability, immune inhibiting potential, M2-like, and phagosome signature) in the Macro-LY6E cluster and Macro-MKI67 identified in this study.
2. Heatmap showing the similarity of the Macro-LY6E cluster identified in this study with the macrophage subclusters from the study by Zhang et al.

(E)UMAP plot of various myeloid subpopulations in the esophageal squamous cell carcinoma (ESCC) dataset (GSE160269).

(F) Vlnplot of the feature genes by the macrophages subpopulations in the ESCC dataset from GSE160269.

(G)Volcano plot showing the differentially expressed genes in the Macro-LY6E cluster and TAM03(SPP1^+^ macrophages) identified in the ESCC dataset from GSE160269.

(H)Vlnplot of the scores of functional gene sets (antigen presentation ability, immune inhibiting potential, M2-like, and phagosome signature) in the Macro-LY6E cluster and SPP1^+^ macrophage identified in the GSE160269 dataset.

(I)UMAP plot of the sample orientation of the macrophages in the ESCC dataset (GSE145370).

(J)UMAP plot of the macrophages’ tissue distribution in the ESCC dataset (GSE145370).

(K)Dotplot of expression profiles of the feature genes in the macrophage subpopulations from the GSE145370 dataset.

(L)Featureplot of the feature gene expression in the macrophage subpopulations from the GSE145370 dataset.

(M)Vlnplot of the scores of functional gene sets (antigen presentation ability, immune inhibiting potential, M2-like, and phagosome signature) across macrophage subpopulations in the ESCC dataset (GSE145370).


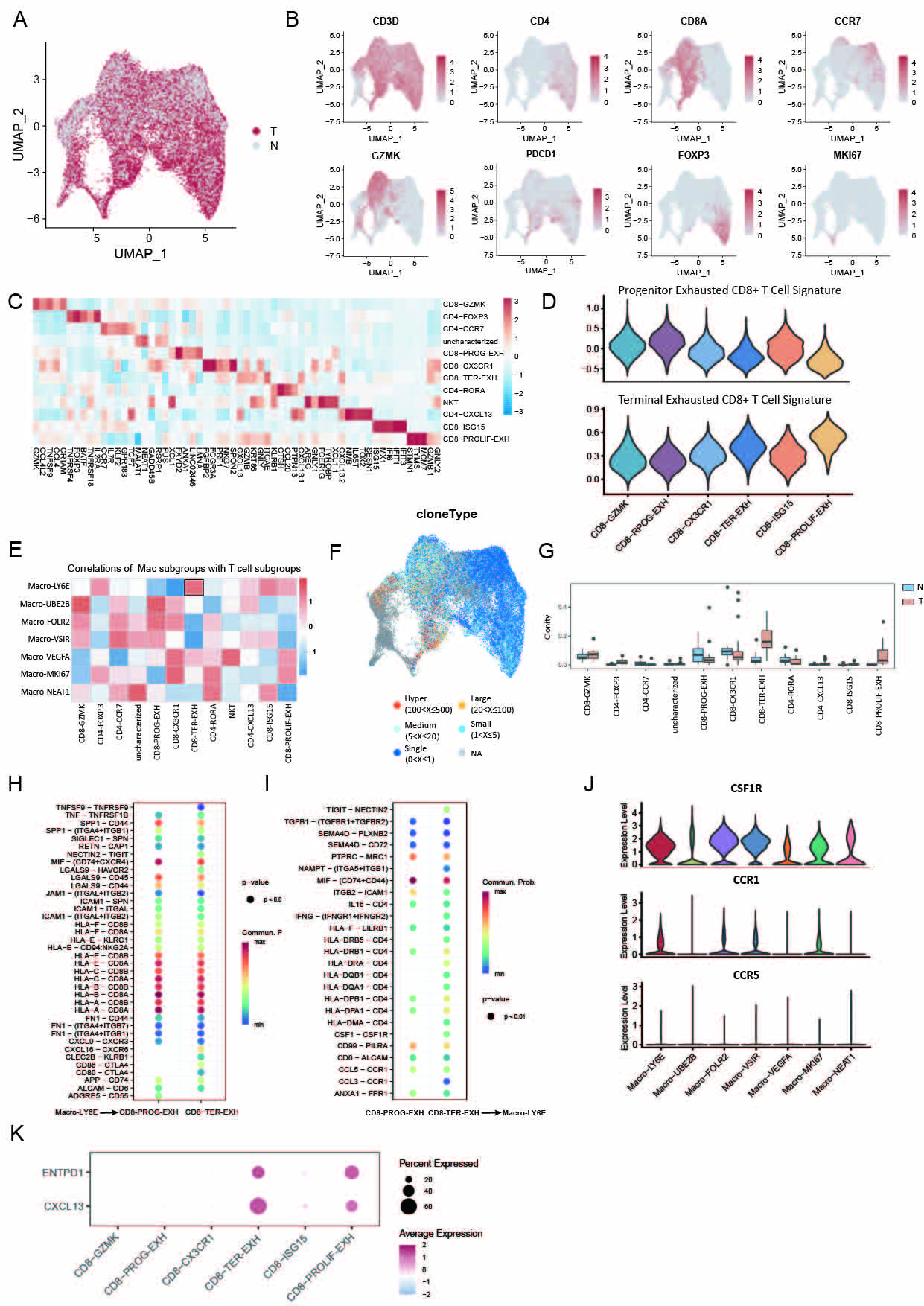


# Supplementary Fig 2. High infiltration of LY6E^+^ macrophages is associated with CD8^+^ Tex cells, related to Figure 4.

**(A)**UMAP plot of total T cells from the ESCC scRNA-seq datasets colored according to normal (N) or tumor (T) tissue origin.

**(B)**UMAP plots of feature genes expressed by the major T cell clusters.

**(C)**Heatmap of the top differentially expressed genes in the major T cell clusters.

**(D)**Vlnplot of the scores of functional gene sets (progenitor exhausted CD8^+^ T cell signature and terminal exhausted CD8^+^ T cell signature) across the CD8^+^ T cells subclusters identified in this study.

**(E)**Heatmap showing correlations between the relative abundances of macrophage subclusters (among total macrophages) and T cell subpopulations (among total T cells) in our in-house ESCC scRNA-seq datasets.

**(F)**UMAP plot showing T cells colored by clone size from the ESCC scRNA-seq datasets.

**(G)**Clonality of T cell subpopulations in normal (N) and tumor (T) tissues from the ESCC scRNA-seq dataset.

1. Summary of molecular interactions of Macro-LY6E with exhausted CD8^+^ T cells subclusters identified in this study.
2. Summary of molecular interactions of exhausted CD8^+^ T cells subclusters with Macro-LY6E identified in this study.
3. Vlnplot of the *CSF1R*,*CCR1*,and *CCR5* by the macrophage subpopulations in our in-house ESCC scRNA-seq datasets.
4. Vlnplot of *CXCL13* and *ENTPD1* by the macrophage subpopulations in our in-house ESCC scRNA-seq datasets.


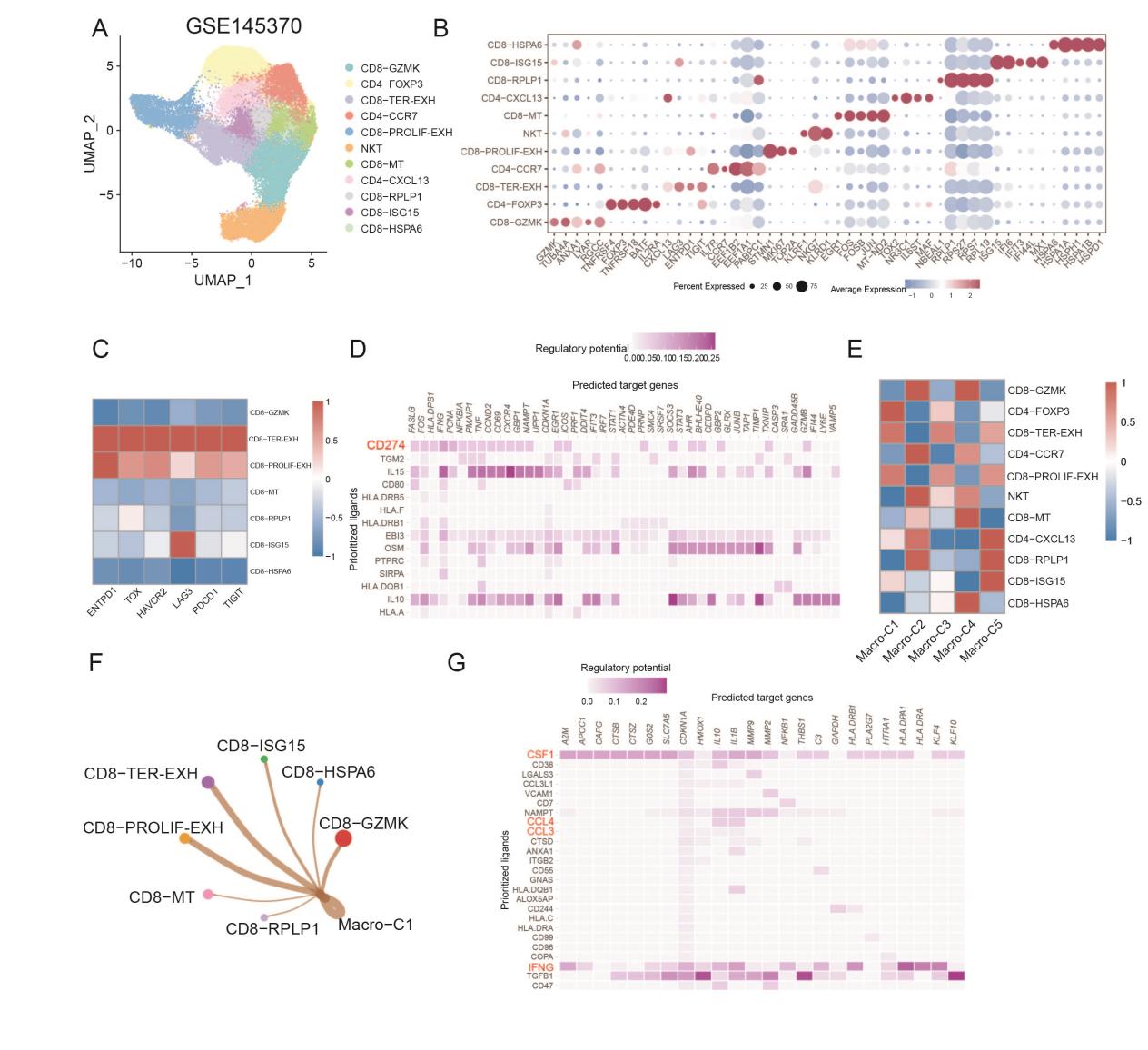


# Supplementary Fig 3. High infiltration of LY6E^+^ macrophages is associated with exhausted CD8^+^ T cells, related to Figure 4.

1. UMAP plot of total T cells from the ESCC scRNA-seq datasets (GSE145370).
2. Dotplot of expression profiles of the feature genes in the T cell subpopulations from the GSE145370 dataset.
3. Heatmap of the expression profiles of the exhaustion-related functional genes in the CD8^+^ T cells subpopulations in the GSE145370 dataset.
4. Heatmap of the regulatory potential of ligands derived from Macro-C1 macrophages that drive CD8+ T cell exhaustion in the ESCC dataset(GSE145370).
5. Heatmap showing correlations between the relative abundances of macrophage subclusters (among total macrophages) and T cell subpopulations (among total T cells) in ESCC scRNA-seq datasets (GSE145370).
6. The significant interaction numbers between Macro-C1 and various CD8^+^ T cells subclusters (GSE145370).
7. Heatmap of the regulatory potential of ligands derived from CD8-TER-EXH cells that modulate the phenotype of Macro-C1 macrophages (GSE145370).

**
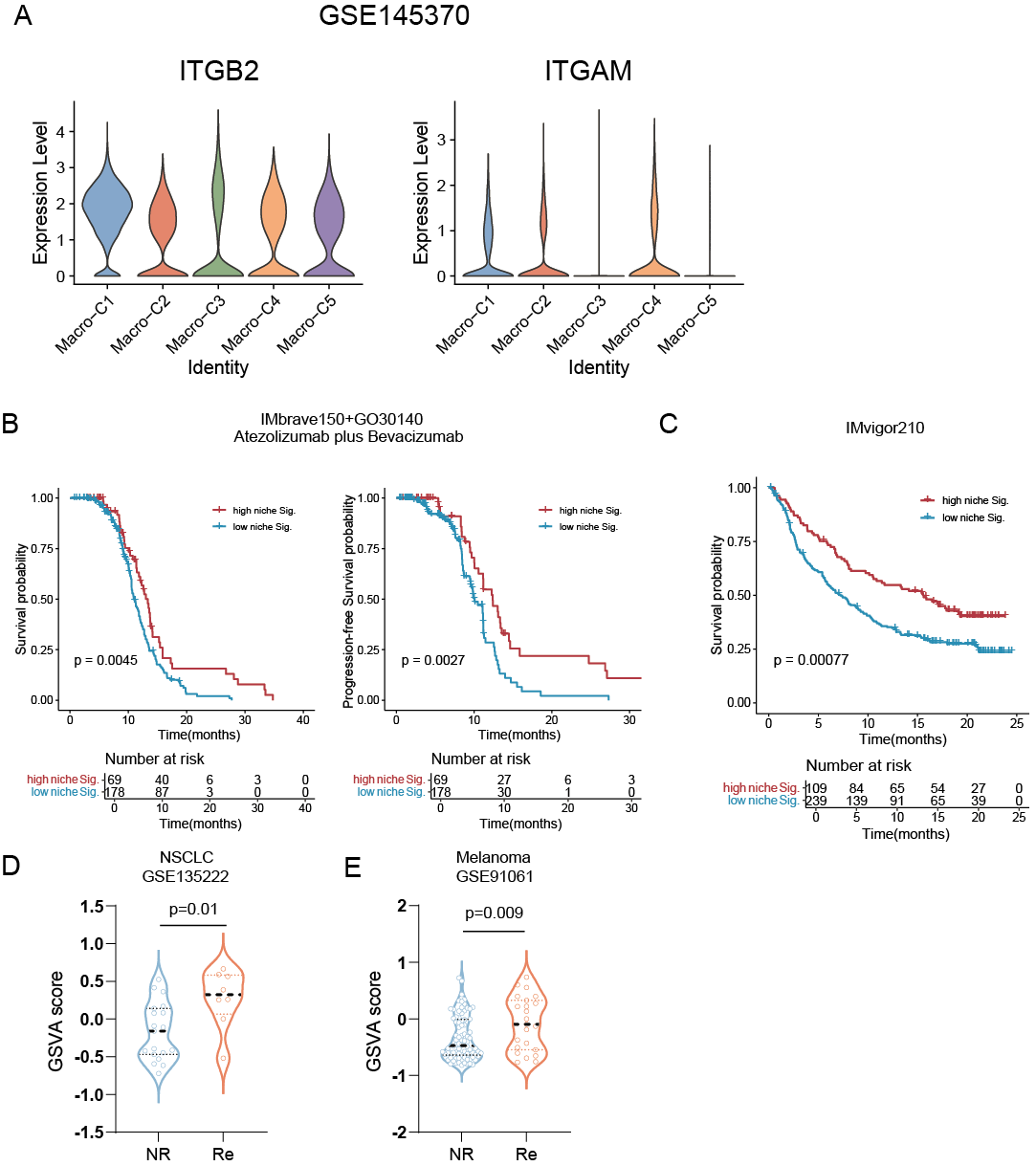
**

**Supplementary Fig 4. Formation of the possible niche of LY6E^+^ macrophages, exhausted CD8^+^ T cells, and ICAM2^+^ tumor cells correlates with immunotherapy response. Related to Figure 5.**

1. Vlnplot of the ITGB2 and ITGAM expression by the macrophage subpopulations from ESCC datasets (GSE145370).

(B) Overall survival (left) and progression-free survival (right) of atezolizumab- and bevacizumab-treated patients stratified by high or low expression of the niche gene signature (n=247). Data are from the GO30140 and IMbrave150 clinical studies.P- values were determined by the log-rank test.

(C) Overall survival analysis of metastatic urothelial carcinoma patients treated with atezolizumab in the IMvigor210 study stratified by the niche signature (n=298). P- value was determined by the log-rank test.

(D) GSVA scores of the niche signature in pre-treatment samples from NSCLC patients treated with anti-PD-1/PD-L1 antibodies in the GSE135222 dataset (n=27). P-value by the Mann-Whitney test.

(E) GSVA scores of the niche signature in melanoma samples receiving immunotherapy (GSE91061 dataset, n=105). P-value by the Mann-Whitney test.
