# Supplementary material for: Identification of a distinct cluster of LY6E+ macrophages in esophageal squamous cell carcinoma: functional phenotype, spatial interaction, and prognostic significance
Source: Br J Cancer. 2026 Apr 29;135(3):382–93. doi: 10.1038/s41416-026-03456-4 (PMC13372808; doi:10.1038/s41416-026-03456-4)
Supplement: Supplementary file 2 — Supplementary table1 [file 41416_2026_3456_MOESM2_ESM.docx]

**Supplementary Tables1| Clinical characteristics of patients in this study**

**Clinical characteristics of 18 patients in the scRNA-seq cohort**

| **Sample ID** | **tissue used for scRNAseq** | **HISTOLOGY** | **pT** | **pN** | **pM** | **pTNM** |
| --- | --- | --- | --- | --- | --- | --- |
| ESCC_P1 | primary tumor | Squamous cell carcinoma | 3 | 3 | 0 | IVA |
| ESCC_P2 | primary tumor | Squamous cell carcinoma | 1b | 0 | 0 | IB |
| ESCC_P3 | primary tumor | Squamous cell carcinoma | 3 | 2 | 0 | IIIB |
| ESCC_P4 | primary tumor | Squamous cell carcinoma | 3 | 2 | 0 | IIIB |
| ESCC_P5 | primary tumor | Squamous cell carcinoma | 3 | 0 | 0 | IIIA |
| ESCC_P6 | primary tumor | Squamous cell carcinoma | 1b | 1 | 0 | IIB |
| ESCC_P7 | primary tumor | Squamous cell carcinoma | 3 | 1 | 0 | IIIB |
| ESCC_P8 | primary tumor | Squamous cell carcinoma | 2 | 2 | 0 | IIIB |
| ESCC_P9 | primary tumor | Squamous cell carcinoma | 3 | 2 | 0 | IIIB |
| ESCC_P10 | primary tumor and adjuvant tissue | Squamous cell carcinoma | 3 | 2 | 0 | IIIB |
| ESCC_P11 | primary tumor and adjuvant tissue | Squamous cell carcinoma | 1b | 0 | 0 | IB |
| ESCC_P12 | primary tumor and adjuvant tissue | Squamous cell carcinoma | 3 | 0 | 0 | IIB |
| ESCC_P13 | primary tumor and adjuvant tissue | Squamous cell carcinoma | 3 | 1 | 0 | IIIB |
| ESCC_P14 | primary tumor and adjuvant tissue | Squamous cell carcinoma | 3 | 0 | 0 | IIB |
| ESCC_P15 | primary tumor and adjuvant tissue | Squamous cell carcinoma | 3 | 1 | 0 | IIIB |
| ESCC_P16 | primary tumor and adjuvant tissue | Squamous cell carcinoma | / | 0 | 0 | / |
| ESCC_P17 | primary tumor and adjuvant tissue | Squamous cell carcinoma | 3 | 0 | 0 | IIA |
| ESCC_P18 | primary tumor and adjuvant tissue | Squamous cell carcinoma | 3 | 2 | 0 | IIIB |
|  |  |  |  |  |  |  |

**Clinical characteristics of 249 patients in the ESCC validation cohort1**

| **Sample ID** | **tissue used for mIHC** | **HISTOLOGY** | **Age(year)** | **Gender** | **Tumor Size(cm)** |
| --- | --- | --- | --- | --- | --- |
| EC_A01 | primary tumor | Squamous cell carcinoma | 70 | Male | 2.40 |
| EC_A02 | primary tumor | Squamous cell carcinoma | 50 | Male | 1.50 |
| EC_A03 | primary tumor | Squamous cell carcinoma | 64 | Male | 2.00 |
| EC_A04 | primary tumor | Squamous cell carcinoma | 64 | Male | 7.00 |
| EC_A05 | primary tumor | Squamous cell carcinoma | 60 | Female | 3.20 |
| EC_A06 | primary tumor | Squamous cell carcinoma | 48 | Male | 5.50 |
| EC_A07 | primary tumor | Squamous cell carcinoma | 53 | Female | 3.00 |
| EC_A08 | primary tumor | Squamous cell carcinoma | 60 | Male | 5.00 |
| EC_A09 | primary tumor | Squamous cell carcinoma | 48 | Male | 6.00 |
| EC_A10 | primary tumor | Squamous cell carcinoma | 57 | Male | 2.70 |
| EC_A11 | primary tumor | Squamous cell carcinoma | 54 | Male | 1.50 |
| EC_A12 | primary tumor | Squamous cell carcinoma | 59 | Male | 4.50 |
| EC_A13 | primary tumor | Squamous cell carcinoma | 71 | Male | 3.00 |
| EC_A14 | primary tumor | Squamous cell carcinoma | 62 | Male | 3.00 |
| EC_A15 | primary tumor | Squamous cell carcinoma | 63 | Male | 8.00 |
| EC_A16 | primary tumor | Squamous cell carcinoma | 61 | Female | 2.80 |
| EC_A17 | primary tumor | Squamous cell carcinoma | 70 | Female | 6.50 |
| EC_A18 | primary tumor | Squamous cell carcinoma | 54 | Male | 2.50 |
| EC_A19 | primary tumor | Squamous cell carcinoma | 59 | Male | 3.50 |
| EC_A20 | primary tumor | Squamous cell carcinoma | 58 | Female | 4.30 |
| EC_A21 | primary tumor | Squamous cell carcinoma | 58 | Male | 4.00 |
| EC_A22 | primary tumor | Squamous cell carcinoma | 52 | Male | 2.00 |
| EC_A23 | primary tumor | Squamous cell carcinoma | 47 | Male | 3.00 |
| EC_A24 | primary tumor | Squamous cell carcinoma | 61 | Female | 5.50 |
| EC_A25 | primary tumor | Squamous cell carcinoma | 60 | Male | 2.50 |
| EC_A26 | primary tumor | Squamous cell carcinoma | 52 | Male | 3.20 |
| EC_A27 | primary tumor | Squamous cell carcinoma | 61 | Female | 2.60 |
| EC_A28 | primary tumor | Squamous cell carcinoma | 67 | Female | 4.00 |
| EC_A29 | primary tumor | Squamous cell carcinoma | 64 | Male | 0.50 |
| EC_A30 | primary tumor | Squamous cell carcinoma | 49 | Male | 3.00 |
| EC_A31 | primary tumor | Squamous cell carcinoma | 61 | Male | 2.00 |
| EC_A32 | primary tumor | Squamous cell carcinoma | 59 | Male | 5.50 |
| EC_A33 | primary tumor | Squamous cell carcinoma | 58 | Male | 3.20 |
| EC_A34 | primary tumor | Squamous cell carcinoma | 57 | Male | 7.50 |
| EC_A35 | primary tumor | Squamous cell carcinoma | 65 | Male | 2.70 |
| EC_A36 | primary tumor | Squamous cell carcinoma | 62 | Male | 3.80 |
| EC_A37 | primary tumor | Squamous cell carcinoma | 66 | Male | 2.00 |
| EC_A38 | primary tumor | Squamous cell carcinoma | 66 | Male | 2.40 |
| EC_A39 | primary tumor | Squamous cell carcinoma | 52 | Male | 4.00 |
| EC_A40 | primary tumor | Squamous cell carcinoma | 66 | Male | 4.50 |
| EC_A41 | primary tumor | Squamous cell carcinoma | 57 | Male | 6.00 |
| EC_A42 | primary tumor | Squamous cell carcinoma | 64 | Male | 3.20 |
| EC_A43 | primary tumor | Squamous cell carcinoma | 56 | Male | 3.00 |
| EC_A44 | primary tumor | Squamous cell carcinoma | 54 | Male | 7.50 |
| EC_A45 | primary tumor | Squamous cell carcinoma | 62 | Male | 1.50 |
| EC_A46 | primary tumor | Squamous cell carcinoma | 57 | Male | 4.50 |
| EC_A47 | primary tumor | Squamous cell carcinoma | 52 | Male | 2.50 |
| EC_A48 | primary tumor | Squamous cell carcinoma | 52 | Male | 1.50 |
| EC_A49 | primary tumor | Squamous cell carcinoma | 58 | Male | 4.50 |
| EC_A50 | primary tumor | Squamous cell carcinoma | 47 | Male | 3.80 |
| EC_A51 | primary tumor | Squamous cell carcinoma | 58 | Male | 4.60 |
| EC_A52 | primary tumor | Squamous cell carcinoma | 60 | Female | 2.50 |
| EC_A53 | primary tumor | Squamous cell carcinoma | 60 | Male | 3.00 |
| EC_A54 | primary tumor | Squamous cell carcinoma | 63 | Male | 2.50 |
| EC_A55 | primary tumor | Squamous cell carcinoma | 58 | Male | 5.00 |
| EC_A56 | primary tumor | Squamous cell carcinoma | 58 | Male | 2.50 |
| EC_A57 | primary tumor | Squamous cell carcinoma | 41 | Male | 4.50 |
| EC_A58 | primary tumor | Squamous cell carcinoma | 54 | Male | 1.00 |
| EC_A59 | primary tumor | Squamous cell carcinoma | 63 | Male | 0.90 |
| EC_A60 | primary tumor | Squamous cell carcinoma | 50 | Male | 2.00 |
| EC_A61 | primary tumor | Squamous cell carcinoma | 61 | Male | 0.70 |
| EC_A62 | primary tumor | Squamous cell carcinoma | 57 | Male | 3.00 |
| EC_A63 | primary tumor | Squamous cell carcinoma | 72 | Female | 3.00 |
| EC_A64 | primary tumor | Squamous cell carcinoma | 57 | Male | 1.50 |
| EC_A65 | primary tumor | Squamous cell carcinoma | 70 | Male | 2.00 |
| EC_A66 | primary tumor | Squamous cell carcinoma | 42 | Male | 6.00 |
| EC_A67 | primary tumor | Squamous cell carcinoma | 55 | Male | 1.60 |
| EC_A68 | primary tumor | Squamous cell carcinoma | 57 | Male | 1.30 |
| EC_A69 | primary tumor | Squamous cell carcinoma | 56 | Female | 9.00 |
| EC_A70 | primary tumor | Squamous cell carcinoma | 49 | Female | 3.70 |
| EC_A71 | primary tumor | Squamous cell carcinoma | 57 | Male | 3.00 |
| EC_A72 | primary tumor | Squamous cell carcinoma | 65 | Male | 5.00 |
| EC_A73 | primary tumor | Squamous cell carcinoma | 69 | Male | 4.50 |
| EC_A74 | primary tumor | Squamous cell carcinoma | 57 | Male | 2.00 |
| EC_A75 | primary tumor | Squamous cell carcinoma | 71 | Male | 2.50 |
| EC_A76 | primary tumor | Squamous cell carcinoma | 60 | Female | 1.30 |
| EC_A77 | primary tumor | Squamous cell carcinoma | 60 | Male | 2.60 |
| EC_A78 | primary tumor | Squamous cell carcinoma | 56 | Female | 4.00 |
| EC_A79 | primary tumor | Squamous cell carcinoma | 64 | Male | 3.00 |
| EC_A80 | primary tumor | Squamous cell carcinoma | 57 | Male | 3.30 |
| EC_A81 | primary tumor | Squamous cell carcinoma | 57 | Male | 4.50 |
| EC_A82 | primary tumor | Squamous cell carcinoma | 63 | Female | 2.20 |
| EC_A83 | primary tumor | Squamous cell carcinoma | 59 | Male | 3.50 |
| EC_A84 | primary tumor | Squamous cell carcinoma | 59 | Male | 3.50 |
| EC_A85 | primary tumor | Squamous cell carcinoma | 59 | Male | 2.50 |
| EC_A86 | primary tumor | Squamous cell carcinoma | 64 | Male | 2.00 |
| EC_A87 | primary tumor | Squamous cell carcinoma | 62 | Male | 3.00 |
| EC_A88 | primary tumor | Squamous cell carcinoma | 56 | Male | 4.00 |
| EC_A89 | primary tumor | Squamous cell carcinoma | 55 | Female | 3.70 |
| EC_A90 | primary tumor | Squamous cell carcinoma | 61 | Male | 2.10 |
| EC_A91 | primary tumor | Squamous cell carcinoma | 46 | Male | 2.90 |
| EC_A92 | primary tumor | Squamous cell carcinoma | 61 | Male | 4.00 |
| EC_A93 | primary tumor | Squamous cell carcinoma | 69 | Male | 1.00 |
| EC_A94 | primary tumor | Squamous cell carcinoma | 55 | Female | 4.00 |
| EC_A95 | primary tumor | Squamous cell carcinoma | 57 | Male | 2.00 |
| EC_A96 | primary tumor | Squamous cell carcinoma | 52 | Male | 2.00 |
| EC_A97 | primary tumor | Squamous cell carcinoma | 60 | Male | 4.00 |
| EC_A98 | primary tumor | Squamous cell carcinoma | 55 | Male | 4.50 |
| EC_A99 | primary tumor | Squamous cell carcinoma | 65 | Female | 2.00 |
| EC_A100 | primary tumor | Squamous cell carcinoma | 46 | Male | 5.00 |
| EC_A101 | primary tumor | Squamous cell carcinoma | 61 | Male | 2.50 |
| EC_A102 | primary tumor | Squamous cell carcinoma | 59 | Male | 4.00 |
| EC_A103 | primary tumor | Squamous cell carcinoma | 66 | Female | 3.50 |
| EC_A104 | primary tumor | Squamous cell carcinoma | 49 | Male | 2.30 |
| EC_A105 | primary tumor | Squamous cell carcinoma | 68 | Male | 5.00 |
| EC_A106 | primary tumor | Squamous cell carcinoma | 45 | Male | 3.40 |
| EC_A107 | primary tumor | Squamous cell carcinoma | 59 | Male | 1.50 |
| EC_A108 | primary tumor | Squamous cell carcinoma | 56 | Male | 3.00 |
| EC_A109 | primary tumor | Squamous cell carcinoma | 62 | Female | 3.60 |
| EC_A110 | primary tumor | Squamous cell carcinoma | 52 | Male | 3.70 |
| EC_A111 | primary tumor | Squamous cell carcinoma | 63 | Male | 2.50 |
| EC_A112 | primary tumor | Squamous cell carcinoma | 57 | Male | 6.50 |
| EC_A113 | primary tumor | Squamous cell carcinoma | 56 | Female | 4.50 |
| EC_A114 | primary tumor | Squamous cell carcinoma | 62 | Female | 3.50 |
| EC_A115 | primary tumor | Squamous cell carcinoma | 61 | Male | 3.00 |
| EC_A116 | primary tumor | Squamous cell carcinoma | 47 | Male | 8.00 |
| EC_A117 | primary tumor | Squamous cell carcinoma | 69 | Male | 4.00 |
| EC_A118 | primary tumor | Squamous cell carcinoma | 43 | Male | 3.00 |
| EC_A119 | primary tumor | Squamous cell carcinoma | 56 | Male | 2.50 |
| EC_A120 | primary tumor | Squamous cell carcinoma | 39 | Male | 2.00 |
| EC_A121 | primary tumor | Squamous cell carcinoma | 73 | Male | 9.00 |
| EC_A122 | primary tumor | Squamous cell carcinoma | 51 | Male | 2.70 |
| EC_A123 | primary tumor | Squamous cell carcinoma | 57 | Male | 0.60 |
| EC_A124 | primary tumor | Squamous cell carcinoma | 61 | Female | 2.00 |
| EC_A125 | primary tumor | Squamous cell carcinoma | 54 | Female | 1.50 |
| EC_A126 | primary tumor | Squamous cell carcinoma | 69 | Female | 4.00 |
| EC_A127 | primary tumor | Squamous cell carcinoma | 72 | Male | 2.50 |
| EC_A128 | primary tumor | Squamous cell carcinoma | 62 | Male | 2.00 |
| EC_A129 | primary tumor | Squamous cell carcinoma | 54 | Male | 4.50 |
| EC_A130 | primary tumor | Squamous cell carcinoma | 45 | Male | 2.80 |
| EC_A131 | primary tumor | Squamous cell carcinoma | 65 | Male | 2.00 |
| EC_A132 | primary tumor | Squamous cell carcinoma | 55 | Male | 3.60 |
| EC_A133 | primary tumor | Squamous cell carcinoma | 57 | Male | 6.00 |
| EC_A134 | primary tumor | Squamous cell carcinoma | 56 | Male | 1.00 |
| EC_A135 | primary tumor | Squamous cell carcinoma | 73 | Male | 2.70 |
| EC_A136 | primary tumor | Squamous cell carcinoma | 55 | Male | 1.20 |
| EC_A137 | primary tumor | Squamous cell carcinoma | 46 | Male | 2.00 |
| EC_A138 | primary tumor | Squamous cell carcinoma | 60 | Male | 2.00 |
| EC_A139 | primary tumor | Squamous cell carcinoma | 60 | Male | 3.40 |
| EC_A140 | primary tumor | Squamous cell carcinoma | 69 | Male | 5.00 |
| EC_A141 | primary tumor | Squamous cell carcinoma | 64 | Male | 2.20 |
| EC_A142 | primary tumor | Squamous cell carcinoma | 60 | Male | 2.20 |
| EC_A143 | primary tumor | Squamous cell carcinoma | 61 | Male | 3.00 |
| EC_A144 | primary tumor | Squamous cell carcinoma | 54 | Female | 2.00 |
| EC_A145 | primary tumor | Squamous cell carcinoma | 55 | Male | 2.50 |
| EC_A146 | primary tumor | Squamous cell carcinoma | 57 | Male | 4.50 |
| EC_A147 | primary tumor | Squamous cell carcinoma | 67 | Female | 2.50 |
| EC_A148 | primary tumor | Squamous cell carcinoma | 64 | Male | 3.00 |
| EC_A149 | primary tumor | Squamous cell carcinoma | 62 | Male | 2.50 |
| EC_A150 | primary tumor | Squamous cell carcinoma | 63 | Male | 1.50 |
| EC_A151 | primary tumor | Squamous cell carcinoma | 61 | Male | 4.50 |
| EC_A152 | primary tumor | Squamous cell carcinoma | 60 | Male | 4.00 |
| EC_A153 | primary tumor | Squamous cell carcinoma | 69 | Male | 3.00 |
| EC_A154 | primary tumor | Squamous cell carcinoma | 60 | Male | 1.50 |
| EC_A155 | primary tumor | Squamous cell carcinoma | 58 | Female | 3.50 |
| EC_A156 | primary tumor | Squamous cell carcinoma | 62 | Male | 3.00 |
| EC_A157 | primary tumor | Squamous cell carcinoma | 65 | Male | 6.00 |
| EC_A158 | primary tumor | Squamous cell carcinoma | 51 | Male | 4.00 |
| EC_A159 | primary tumor | Squamous cell carcinoma | 61 | Male | 0.70 |
| EC_A160 | primary tumor | Squamous cell carcinoma | 56 | Male | 4.00 |
| EC_A161 | primary tumor | Squamous cell carcinoma | 50 | Male | 2.00 |
| EC_A162 | primary tumor | Squamous cell carcinoma | 57 | Male | 2.50 |
| EC_A163 | primary tumor | Squamous cell carcinoma | 56 | Male | 3.50 |
| EC_A164 | primary tumor | Squamous cell carcinoma | 66 | Female | 3.50 |
| EC_A165 | primary tumor | Squamous cell carcinoma | 72 | Male | 3.50 |
| EC_A166 | primary tumor | Squamous cell carcinoma | 57 | Male | 3.50 |
| EC_A167 | primary tumor | Squamous cell carcinoma | 70 | Female | 4.00 |
| EC_A168 | primary tumor | Squamous cell carcinoma | 47 | Female | 2.50 |
| EC_A169 | primary tumor | Squamous cell carcinoma | 56 | Male | 6.20 |
| EC_A170 | primary tumor | Squamous cell carcinoma | 63 | Female | 4.00 |
| EC_A171 | primary tumor | Squamous cell carcinoma | 61 | Male | 4.50 |
| EC_A172 | primary tumor | Squamous cell carcinoma | 64 | Male | 2.50 |
| EC_A173 | primary tumor | Squamous cell carcinoma | 73 | Male | 3.50 |
| EC_A174 | primary tumor | Squamous cell carcinoma | 32 | Male | 1.80 |
| EC_A175 | primary tumor | Squamous cell carcinoma | 39 | Female | 3.70 |
| EC_A176 | primary tumor | Squamous cell carcinoma | 61 | Male | 5.00 |
| EC_A177 | primary tumor | Squamous cell carcinoma | 50 | Male | 2.00 |
| EC_A178 | primary tumor | Squamous cell carcinoma | 74 | Male | 4.00 |
| EC_A179 | primary tumor | Squamous cell carcinoma | 68 | Male | 3.00 |
| EC_A180 | primary tumor | Squamous cell carcinoma | 61 | Male | 2.00 |
| EC_A181 | primary tumor | Squamous cell carcinoma | 60 | Male | 2.00 |
| EC_A182 | primary tumor | Squamous cell carcinoma | 66 | Male | 2.50 |
| EC_A183 | primary tumor | Squamous cell carcinoma | 59 | Male | 3.00 |
| EC_A184 | primary tumor | Squamous cell carcinoma | 61 | Male | 3.00 |
| EC_A185 | primary tumor | Squamous cell carcinoma | 59 | Male | 3.50 |
| EC_A186 | primary tumor | Squamous cell carcinoma | 58 | Male | 7.80 |
| EC_A187 | primary tumor | Squamous cell carcinoma | 65 | Male | 3.70 |
| EC_A188 | primary tumor | Squamous cell carcinoma | 49 | Male | 2.10 |
| EC_A189 | primary tumor | Squamous cell carcinoma | 55 | Male | 4.00 |
| EC_A190 | primary tumor | Squamous cell carcinoma | 62 | Female | 3.00 |
| EC_A191 | primary tumor | Squamous cell carcinoma | 50 | Male | 3.00 |
| EC_A192 | primary tumor | Squamous cell carcinoma | 68 | Male | 3.70 |
| EC_A193 | primary tumor | Squamous cell carcinoma | 62 | Male | 4.60 |
| EC_A194 | primary tumor | Squamous cell carcinoma | 69 | Male | 3.50 |
| EC_A195 | primary tumor | Squamous cell carcinoma | 59 | Female | 2.50 |
| EC_A196 | primary tumor | Squamous cell carcinoma | / | Male | 4.30 |
| EC_A197 | primary tumor | Squamous cell carcinoma | 64 | Male | 4.00 |
| EC_A198 | primary tumor | Squamous cell carcinoma | 69 | Male | 4.50 |
| EC_A199 | primary tumor | Squamous cell carcinoma | 58 | Male | 3.00 |
| EC_A200 | primary tumor | Squamous cell carcinoma | 55 | Male | 2.00 |
| EC_A201 | primary tumor | Squamous cell carcinoma | 56 | Male | 4.80 |
| EC_A202 | primary tumor | Squamous cell carcinoma | 61 | Female | 2.00 |
| EC_A203 | primary tumor | Squamous cell carcinoma | 61 | Male | 4.30 |
| EC_A204 | primary tumor | Squamous cell carcinoma | 50 | Male | 1.00 |
| EC_A205 | primary tumor | Squamous cell carcinoma | 50 | Male | 2.20 |
| EC_A206 | primary tumor | Squamous cell carcinoma | 55 | Male | 2.00 |
| EC_A207 | primary tumor | Squamous cell carcinoma | 59 | Male | 1.20 |
| EC_A208 | primary tumor | Squamous cell carcinoma | 47 | Female | 1.50 |
| EC_A209 | primary tumor | Squamous cell carcinoma | 63 | Male | 2.50 |
| EC_A210 | primary tumor | Squamous cell carcinoma | 61 | Male | 4.50 |
| EC_A211 | primary tumor | Squamous cell carcinoma | 62 | Male | 3.00 |
| EC_A212 | primary tumor | Squamous cell carcinoma | 64 | Male | 0.80 |
| EC_A213 | primary tumor | Squamous cell carcinoma | 69 | Male | 3.00 |
| EC_A214 | primary tumor | Squamous cell carcinoma | 58 | Female | 3.00 |
| EC_A215 | primary tumor | Squamous cell carcinoma | 50 | Female | 4.80 |
| EC_A216 | primary tumor | Squamous cell carcinoma | 64 | Male | 4.00 |
| EC_A217 | primary tumor | Squamous cell carcinoma | 67 | Male | 1.20 |
| EC_A218 | primary tumor | Squamous cell carcinoma | 56 | Male | 4.00 |
| EC_A219 | primary tumor | Squamous cell carcinoma | 59 | Male | 3.50 |
| EC_A220 | primary tumor | Squamous cell carcinoma | 66 | Male | 1.00 |
| EC_A221 | primary tumor | Squamous cell carcinoma | 71 | Male | 3.20 |
| EC_A222 | primary tumor | Squamous cell carcinoma | 67 | Male | 2.00 |
| EC_A223 | primary tumor | Squamous cell carcinoma | 57 | Male | 2.50 |
| EC_A224 | primary tumor | Squamous cell carcinoma | 68 | Male | 2.00 |
| EC_A225 | primary tumor | Squamous cell carcinoma | 66 | Male | 8.00 |
| EC_A226 | primary tumor | Squamous cell carcinoma | 62 | Male | 2.50 |
| EC_A227 | primary tumor | Squamous cell carcinoma | 73 | Male | 2.50 |
| EC_A228 | primary tumor | Squamous cell carcinoma | 61 | Male | 2.00 |
| EC_A229 | primary tumor | Squamous cell carcinoma | 50 | Male | 2.80 |
| EC_A230 | primary tumor | Squamous cell carcinoma | 65 | Male | 2.00 |
| EC_A231 | primary tumor | Squamous cell carcinoma | 64 | Male | 2.00 |
| EC_A232 | primary tumor | Squamous cell carcinoma | 63 | Male | 2.00 |
| EC_A233 | primary tumor | Squamous cell carcinoma | 58 | Male | 1.50 |
| EC_A234 | primary tumor | Squamous cell carcinoma | 55 | Female | 5.00 |
| EC_A235 | primary tumor | Squamous cell carcinoma | 59 | Male | 4.30 |
| EC_A236 | primary tumor | Squamous cell carcinoma | 50 | Male | 2.00 |
| EC_A237 | primary tumor | Squamous cell carcinoma | 51 | Female | 3.50 |
| EC_A238 | primary tumor | Squamous cell carcinoma | 44 | Male | 1.00 |
| EC_A239 | primary tumor | Squamous cell carcinoma | 73 | Male | 3.00 |
| EC_A240 | primary tumor | Squamous cell carcinoma | 63 | Male | 3.00 |
| EC_A241 | primary tumor | Squamous cell carcinoma | 62 | Male | 1.00 |
| EC_A242 | primary tumor | Squamous cell carcinoma | 53 | Male | 3.50 |
| EC_A243 | primary tumor | Squamous cell carcinoma | 66 | Male | 3.50 |
| EC_A244 | primary tumor | Squamous cell carcinoma | 68 | Male | 1.50 |
| EC_A245 | primary tumor | Squamous cell carcinoma | 69 | Male | 2.00 |
| EC_A246 | primary tumor | Squamous cell carcinoma | 54 | Male | 2.00 |
| EC_A247 | primary tumor | Squamous cell carcinoma | 60 | Male | 3.50 |
| EC_A248 | primary tumor | Squamous cell carcinoma | 61 | Female | 2.20 |
| EC_A249 | primary tumor | Squamous cell carcinoma | 70 | Male | 2.50 |

**Clinical characteristics of 22 patients receiving neoadjuant immunotherapy**

| **Sample ID** | **HISTOLOGY** | **Age(year)** | **Sex** | **Pathogical Response Evaluation Grade** | **Pathogical Response Evaluation** |
| --- | --- | --- | --- | --- | --- |
| EC_IT01 | Squamous cell carcinoma | 66 | Male | 1 | pCR |
| EC_IT02 | Squamous cell carcinoma | 73 | Male | 1 | pCR |
| EC_IT03 | Squamous cell carcinoma | 57 | Male | 3 | non-pCR |
| EC_IT04 | Squamous cell carcinoma | 52 | Male | 1 | pCR |
| EC_IT05 | Squamous cell carcinoma | 42 | Male | 3 | non-pCR |
| EC_IT06 | Squamous cell carcinoma | 53 | Male | 1 | pCR |
| EC_IT07 | Squamous cell carcinoma | 49 | Male | 3 | non-pCR |
| EC_IT08 | Squamous cell carcinoma | 54 | Male | 2 | non-pCR |
| EC_IT09 | Squamous cell carcinoma | 67 | Female | 2 | non-pCR |
| EC_IT10 | Squamous cell carcinoma | 62 | Male | 1 | pCR |
| EC_IT11 | Squamous cell carcinoma | 62 | Male | 3 | non-pCR |
| EC_IT12 | Squamous cell carcinoma | 59 | Male | 3 | non-pCR |
| EC_IT13 | Squamous cell carcinoma | 68 | Female | 3 | non-pCR |
| EC_IT14 | Squamous cell carcinoma | 60 | Male | 3 | non-pCR |
| EC_IT15 | Squamous cell carcinoma | 66 | Male | 3 | non-pCR |
| EC_IT16 | Squamous cell carcinoma | 72 | Male | 1 | pCR |
| EC_IT17 | Squamous cell carcinoma | 57 | Male | 3 | non-pCR |
| EC_IT18 | Squamous cell carcinoma | 47 | Male | 3 | non-pCR |
| EC_IT19 | Squamous cell carcinoma | 61 | Male | 3 | non-pCR |
| EC_IT20 | Squamous cell carcinoma | 63 | Female | 2 | non-pCR |
| EC_IT21 | Squamous cell carcinoma | 76 | Male | 1 | pCR |
| EC_IT22 | Squamous cell carcinoma | 70 | Male | 2 | non-pCR |
