# Supplementary material for: Identification of a distinct cluster of LY6E+ macrophages in esophageal squamous cell carcinoma: functional phenotype, spatial interaction, and prognostic significance
Source: Br J Cancer. 2026 Apr 29;135(3):382–93. doi: 10.1038/s41416-026-03456-4 (PMC13372808; doi:10.1038/s41416-026-03456-4)
Supplement: Supplementary file 3 — Supplementary table2 [file 41416_2026_3456_MOESM3_ESM.docx]

**Supplementary Tables2| differently expressed genes in myeloid or T cells subclusters**

**Feature genes expressed by macrophages subsets of our in-house ESCC scRNA-seq**

| p_val | avg_log2FC | pct.1 | pct.2 | p_val_adj | cluster | gene |
| --- | --- | --- | --- | --- | --- | --- |
| 0 | 1.873713595 | 0.883 | 0.423 | 0 | Macro-LY6E | APOE |
| 0 | 1.725925976 | 0.902 | 0.42 | 0 | Macro-LY6E | APOC1 |
| 0 | 1.371180472 | 0.729 | 0.321 | 0 | Macro-LY6E | SPP1 |
| 0 | 1.3175339 | 0.836 | 0.333 | 0 | Macro-LY6E | TREM2 |
| 0 | 1.182398723 | 0.914 | 0.527 | 0 | Macro-LY6E | ACP5 |
| 0 | 1.128614494 | 0.878 | 0.468 | 0 | Macro-LY6E | IFI6 |
| 0 | 1.092338068 | 0.844 | 0.413 | 0 | Macro-LY6E | LIPA |
| 0 | 1.035935992 | 0.889 | 0.501 | 0 | Macro-LY6E | FABP5 |
| 0 | 0.99199716 | 0.81 | 0.319 | 0 | Macro-LY6E | PLA2G7 |
| 0 | 0.950231695 | 0.837 | 0.417 | 0 | Macro-LY6E | CD9 |
| 0 | 0.948907634 | 0.973 | 0.675 | 0 | Macro-LY6E | CTSD |
| 0 | 0.919065881 | 0.999 | 0.902 | 0 | Macro-LY6E | CTSB |
| 0 | 0.87999089 | 0.866 | 0.439 | 0 | Macro-LY6E | GPNMB |
| 0 | 0.835258474 | 0.891 | 0.503 | 0 | Macro-LY6E | LY6E |
| 0 | 0.817898295 | 0.982 | 0.654 | 0 | Macro-LY6E | CAPG |
| 0 | 0.813880769 | 0.793 | 0.405 | 0 | Macro-LY6E | ISG15 |
| 0 | 0.770560074 | 0.912 | 0.61 | 0 | Macro-LY6E | PLD3 |
| 0 | 0.763056986 | 0.945 | 0.625 | 0 | Macro-LY6E | CTSL |
| 0 | 0.692107778 | 0.819 | 0.377 | 0 | Macro-LY6E | GM2A |
| 0 | 0.689229258 | 0.97 | 0.723 | 0 | Macro-LY6E | PRDX1 |
| 0 | 0.66607391 | 0.448 | 0.129 | 0 | Macro-LY6E | ADAMDEC1 |
| 0 | 0.661216163 | 0.905 | 0.541 | 0 | Macro-LY6E | BCAP31 |
| 0 | 0.650470016 | 0.982 | 0.778 | 0 | Macro-LY6E | GRN |
| 0 | 0.641234374 | 0.543 | 0.19 | 0 | Macro-LY6E | GCHFR |
| 0 | 0.635293034 | 0.998 | 0.894 | 0 | Macro-LY6E | PSAP |
| 0 | 0.635224685 | 0.886 | 0.512 | 0 | Macro-LY6E | CTSA |
| 0 | 0.624960932 | 0.938 | 0.575 | 0 | Macro-LY6E | CD81 |
| 0 | 0.617687647 | 0.991 | 0.82 | 0 | Macro-LY6E | CD63 |
| 0 | 0.594801148 | 0.993 | 0.832 | 0 | Macro-LY6E | CD68 |
| 0 | 0.591125214 | 0.607 | 0.219 | 0 | Macro-LY6E | IL4I1 |
| 0 | 0.588020838 | 0.995 | 0.836 | 0 | Macro-LY6E | LAPTM5 |
| 0 | 0.569751072 | 0.648 | 0.248 | 0 | Macro-LY6E | DNASE2 |
| 0 | 0.560830911 | 0.752 | 0.365 | 0 | Macro-LY6E | SCPEP1 |
| 0 | 0.55290134 | 0.68 | 0.301 | 0 | Macro-LY6E | LGALS3BP |
| 0 | 0.536726561 | 0.685 | 0.289 | 0 | Macro-LY6E | CAMK1 |
| 0 | 0.525020069 | 0.813 | 0.41 | 0 | Macro-LY6E | SLC16A3 |
| 0 | 0.51726149 | 0.801 | 0.429 | 0 | Macro-LY6E | MYDGF |
| 0 | 0.509144392 | 0.88 | 0.508 | 0 | Macro-LY6E | LGALS9 |
| 0 | 0.497280698 | 0.814 | 0.424 | 0 | Macro-LY6E | SCARB2 |
| 0 | 0.489791321 | 0.339 | 0.077 | 0 | Macro-LY6E | MATK |
| 0 | 0.483945652 | 0.527 | 0.167 | 0 | Macro-LY6E | NR1H3 |
| 0 | 0.478378727 | 0.791 | 0.378 | 0 | Macro-LY6E | MMP14 |
| 0 | 0.476930519 | 0.634 | 0.264 | 0 | Macro-LY6E | CD40 |
| 0 | 0.476672917 | 0.62 | 0.236 | 0 | Macro-LY6E | MFSD12 |
| 0 | 0.425625249 | 0.646 | 0.254 | 0 | Macro-LY6E | SLC31A1 |
| 0 | 0.392707678 | 0.626 | 0.242 | 0 | Macro-LY6E | ACP2 |
| 0 | 0.385163036 | 0.464 | 0.122 | 0 | Macro-LY6E | HSD3B7 |
| 0 | 0.373563676 | 0.568 | 0.22 | 0 | Macro-LY6E | EVL |
| 0 | 0.362400212 | 0.497 | 0.161 | 0 | Macro-LY6E | CD276 |
| 0 | 0.355615058 | 0.349 | 0.088 | 0 | Macro-LY6E | RAB7B |
| 5.7362E-152 | 0.378684367 | 0.228 | 0.645 | 1.6048E-147 | Macro-UBE2B | UBE2B |
| 2.7356E-145 | 0.304396393 | 0.173 | 0.532 | 7.653E-141 | Macro-UBE2B | SF3B6 |
| 4.8389E-138 | 0.259890066 | 0.275 | 0.703 | 1.3537E-133 | Macro-UBE2B | DAD1 |
| 2.1812E-128 | 0.322277659 | 0.195 | 0.551 | 6.1022E-124 | Macro-UBE2B | FAM50A |
| 1.9854E-127 | 0.274600536 | 0.103 | 0.376 | 5.5543E-123 | Macro-UBE2B | VPS4B |
| 5.0625E-127 | 0.33060365 | 0.173 | 0.502 | 1.4163E-122 | Macro-UBE2B | SEC14L1 |
| 5.7234E-125 | 0.251972248 | 0.111 | 0.388 | 1.6012E-120 | Macro-UBE2B | CHMP2B |
| 2.1437E-123 | 0.575055008 | 0.205 | 0.565 | 5.9972E-119 | Macro-UBE2B | ANP32A |
| 1.9255E-121 | 0.256295942 | 0.211 | 0.567 | 5.3868E-117 | Macro-UBE2B | NDUFC1 |
| 1.3123E-119 | 0.268036565 | 0.23 | 0.601 | 3.6713E-115 | Macro-UBE2B | ISCU |
| 1.7943E-118 | 0.35714004 | 0.13 | 0.415 | 5.0197E-114 | Macro-UBE2B | FAM177A1 |
| 5.6496E-118 | 0.273361183 | 0.12 | 0.394 | 1.5805E-113 | Macro-UBE2B | SMIM29 |
| 1.2414E-117 | 0.255296624 | 0.266 | 0.666 | 3.473E-113 | Macro-UBE2B | DYNLRB1 |
| 3.0305E-117 | 0.251943649 | 0.127 | 0.405 | 8.4781E-113 | Macro-UBE2B | HSD17B4 |
| 8.7331E-117 | 0.338034331 | 0.126 | 0.403 | 2.4432E-112 | Macro-UBE2B | LAGE3 |
| 1.615E-115 | 0.322958946 | 0.311 | 0.732 | 4.5181E-111 | Macro-UBE2B | MAP1LC3B |
| 1.6707E-113 | 0.279418436 | 0.124 | 0.395 | 4.6741E-109 | Macro-UBE2B | MMP24OS |
| 2.4109E-112 | 0.29453654 | 0.234 | 0.587 | 6.7448E-108 | Macro-UBE2B | SMAP2 |
| 1.2355E-110 | 0.315522112 | 0.202 | 0.537 | 3.4564E-106 | Macro-UBE2B | ANAPC16 |
| 6.5828E-110 | 0.287240088 | 0.241 | 0.602 | 1.8416E-105 | Macro-UBE2B | CARD16 |
| 1.2295E-109 | 0.402111451 | 0.186 | 0.506 | 3.4398E-105 | Macro-UBE2B | GNPDA1 |
| 1.9652E-108 | 0.343309185 | 0.279 | 0.659 | 5.4978E-104 | Macro-UBE2B | EFHD2 |
| 9.2468E-108 | 0.276433838 | 0.18 | 0.49 | 2.5869E-103 | Macro-UBE2B | DSTN |
| 1.3246E-107 | 0.319952049 | 0.11 | 0.363 | 3.7058E-103 | Macro-UBE2B | IRF2 |
| 1.5443E-107 | 0.258923993 | 0.235 | 0.581 | 4.3203E-103 | Macro-UBE2B | UBE2L6 |
| 4.9273E-103 | 0.27135463 | 0.293 | 0.686 | 1.37847E-98 | Macro-UBE2B | ATP5MPL |
| 1.25645E-100 | 0.254149359 | 0.326 | 0.756 | 3.51504E-96 | Macro-UBE2B | DAZAP2 |
| 6.33702E-99 | 0.269391115 | 0.084 | 0.302 | 1.77284E-94 | Macro-UBE2B | MTHFS |
| 5.25943E-98 | 0.275970571 | 0.156 | 0.431 | 1.47138E-93 | Macro-UBE2B | NPL |
| 1.12721E-93 | 0.48989898 | 0.091 | 0.307 | 3.15349E-89 | Macro-UBE2B | CIR1 |
| 2.83276E-92 | 0.260153081 | 0.277 | 0.645 | 7.92492E-88 | Macro-UBE2B | C9orf16 |
| 5.36446E-91 | 0.46625805 | 0.152 | 0.413 | 1.50076E-86 | Macro-UBE2B | TAF10 |
| 1.58065E-89 | 0.391295847 | 0.313 | 0.716 | 4.42202E-85 | Macro-UBE2B | TRMT112 |
| 6.49673E-88 | 0.27170697 | 0.339 | 0.747 | 1.81753E-83 | Macro-UBE2B | TMEM258 |
| 4.32881E-87 | 0.314429921 | 0.34 | 0.747 | 1.21103E-82 | Macro-UBE2B | SNX3 |
| 4.90021E-87 | 0.300840374 | 0.265 | 0.617 | 1.37088E-82 | Macro-UBE2B | NDUFA3 |
| 1.17784E-86 | 0.30153971 | 0.308 | 0.687 | 3.29514E-82 | Macro-UBE2B | RPS27L |
| 4.54822E-86 | 0.366808754 | 0.299 | 0.679 | 1.27241E-81 | Macro-UBE2B | PRDX5 |
| 5.49082E-85 | 0.318927947 | 0.351 | 0.767 | 1.53611E-80 | Macro-UBE2B | CNBP |
| 2.13057E-84 | 0.282244749 | 0.332 | 0.734 | 5.96048E-80 | Macro-UBE2B | GMFG |
| 1.28411E-82 | 0.26637732 | 0.355 | 0.767 | 3.59243E-78 | Macro-UBE2B | SKP1 |
| 3.78632E-82 | 0.314967809 | 0.298 | 0.664 | 1.05926E-77 | Macro-UBE2B | ATP5ME |
| 5.24634E-82 | 0.968077355 | 0.257 | 0.599 | 1.46772E-77 | Macro-UBE2B | GABARAPL2 |
| 4.14786E-76 | 0.374082102 | 0.213 | 0.491 | 1.16041E-71 | Macro-UBE2B | NCF1 |
| 4.34986E-76 | 0.26482053 | 0.333 | 0.714 | 1.21692E-71 | Macro-UBE2B | POLR2L |
| 6.5754E-76 | 0.275676587 | 0.352 | 0.756 | 1.83953E-71 | Macro-UBE2B | SH3BGRL |
| 7.88684E-74 | 0.322487118 | 0.082 | 0.261 | 2.20642E-69 | Macro-UBE2B | CLEC4E |
| 3.82352E-73 | 0.419499202 | 0.093 | 0.279 | 1.06967E-68 | Macro-UBE2B | MARCKSL1 |
| 5.82117E-73 | 0.778696844 | 0.228 | 0.525 | 1.62853E-68 | Macro-UBE2B | CYSTM1 |
| 4.75458E-72 | 0.30219428 | 0.362 | 0.764 | 1.33014E-67 | Macro-UBE2B | BRK1 |
| 0 | 2.552651443 | 0.926 | 0.306 | 0 | Macro-FOLR2 | SELENOP |
| 0 | 2.506165361 | 0.872 | 0.296 | 0 | Macro-FOLR2 | F13A1 |
| 0 | 2.326341671 | 0.571 | 0.084 | 0 | Macro-FOLR2 | LYVE1 |
| 0 | 2.192268905 | 0.891 | 0.493 | 0 | Macro-FOLR2 | PLTP |
| 0 | 2.173755072 | 0.889 | 0.322 | 0 | Macro-FOLR2 | FOLR2 |
| 0 | 1.987046967 | 0.619 | 0.215 | 0 | Macro-FOLR2 | PDK4 |
| 0 | 1.93123413 | 0.893 | 0.556 | 0 | Macro-FOLR2 | EGR1 |
| 0 | 1.917682516 | 0.842 | 0.461 | 0 | Macro-FOLR2 | CFD |
| 0 | 1.880510275 | 0.958 | 0.746 | 0 | Macro-FOLR2 | JUN |
| 0 | 1.793119288 | 0.98 | 0.597 | 0 | Macro-FOLR2 | RNASE1 |
| 0 | 1.657534058 | 0.679 | 0.186 | 0 | Macro-FOLR2 | LILRB5 |
| 0 | 1.577762759 | 0.929 | 0.533 | 0 | Macro-FOLR2 | STAB1 |
| 0 | 1.5152349 | 0.904 | 0.655 | 0 | Macro-FOLR2 | FOSB |
| 0 | 1.494709306 | 0.908 | 0.676 | 0 | Macro-FOLR2 | IER2 |
| 0 | 1.472061297 | 0.505 | 0.195 | 0 | Macro-FOLR2 | MAN1A1 |
| 0 | 1.467640304 | 0.883 | 0.545 | 0 | Macro-FOLR2 | MRC1 |
| 0 | 1.464317705 | 0.429 | 0.034 | 0 | Macro-FOLR2 | MAMDC2 |
| 0 | 1.442074 | 0.614 | 0.214 | 0 | Macro-FOLR2 | EMB |
| 0 | 1.418193963 | 0.923 | 0.644 | 0 | Macro-FOLR2 | DAB2 |
| 0 | 1.409401801 | 0.921 | 0.619 | 0 | Macro-FOLR2 | CD163 |
| 0 | 1.374795385 | 0.973 | 0.823 | 0 | Macro-FOLR2 | FOS |
| 0 | 1.327576645 | 0.878 | 0.59 | 0 | Macro-FOLR2 | MS4A4A |
| 0 | 1.295488354 | 0.527 | 0.126 | 0 | Macro-FOLR2 | COLEC12 |
| 0 | 1.292314845 | 0.72 | 0.347 | 0 | Macro-FOLR2 | MAF |
| 0 | 1.272700738 | 0.385 | 0.097 | 0 | Macro-FOLR2 | CCL13 |
| 0 | 1.272165762 | 0.351 | 0.033 | 0 | Macro-FOLR2 | TTN |
| 0 | 1.221349497 | 0.783 | 0.392 | 0 | Macro-FOLR2 | SLC40A1 |
| 0 | 1.201589666 | 0.724 | 0.382 | 0 | Macro-FOLR2 | GPR34 |
| 0 | 1.17508422 | 0.555 | 0.146 | 0 | Macro-FOLR2 | LTC4S |
| 0 | 1.143697556 | 0.899 | 0.67 | 0 | Macro-FOLR2 | MAFB |
| 0 | 1.043414268 | 0.957 | 0.766 | 0 | Macro-FOLR2 | FCGRT |
| 0 | 1.035327898 | 0.822 | 0.512 | 0 | Macro-FOLR2 | SLCO2B1 |
| 0 | 0.849989273 | 0.968 | 0.728 | 0 | Macro-FOLR2 | MS4A6A |
| 0 | 0.847679117 | 0.295 | 0.041 | 0 | Macro-FOLR2 | EGFL7 |
| 0 | 0.827291118 | 0.996 | 0.789 | 0 | Macro-FOLR2 | C1QA |
| 0 | 0.824689557 | 0.363 | 0.082 | 0 | Macro-FOLR2 | IGF1 |
| 0 | 0.794548441 | 0.998 | 0.912 | 0 | Macro-FOLR2 | CST3 |
| 7.2382E-299 | 0.935584137 | 0.572 | 0.242 | 2.0249E-294 | Macro-FOLR2 | MTSS1 |
| 1.762E-293 | 0.979131223 | 0.653 | 0.351 | 4.9295E-289 | Macro-FOLR2 | AP2A2 |
| 3.944E-292 | 1.161696131 | 0.845 | 0.611 | 1.1034E-287 | Macro-FOLR2 | AHNAK |
| 1.4738E-291 | 0.865547725 | 0.419 | 0.127 | 4.123E-287 | Macro-FOLR2 | CD163L1 |
| 3.0082E-291 | 0.832240178 | 0.401 | 0.119 | 8.4157E-287 | Macro-FOLR2 | PDGFC |
| 3.4867E-289 | 0.911580333 | 0.933 | 0.729 | 9.7543E-285 | Macro-FOLR2 | DNAJB1 |
| 2.4711E-288 | 1.009037201 | 0.723 | 0.408 | 6.9131E-284 | Macro-FOLR2 | VSIG4 |
| 4.0863E-287 | 1.097005523 | 0.896 | 0.644 | 1.1432E-282 | Macro-FOLR2 | LGMN |
| 8.7907E-285 | 1.028014156 | 0.619 | 0.317 | 2.4593E-280 | Macro-FOLR2 | GAS6 |
| 8.1858E-280 | 0.783671416 | 0.358 | 0.098 | 2.2901E-275 | Macro-FOLR2 | WLS |
| 5.4175E-279 | 0.963000853 | 0.967 | 0.809 | 1.5156E-274 | Macro-FOLR2 | HSPA1A |
| 2.7407E-269 | 0.708517707 | 0.988 | 0.895 | 7.6673E-265 | Macro-FOLR2 | ITM2B |
| 9.7638E-268 | 0.925368998 | 0.806 | 0.587 | 2.7315E-263 | Macro-FOLR2 | BLVRB |
| 0 | 1.359024093 | 0.626 | 0.17 | 0 | Macro-VSIR | CLEC10A |
| 0 | 0.966955909 | 0.552 | 0.16 | 0 | Macro-VSIR | JAML |
| 7.3873E-276 | 1.177359913 | 0.319 | 0.068 | 2.0667E-271 | Macro-VSIR | FCN1 |
| 8.1518E-266 | 0.880294871 | 0.984 | 0.822 | 2.2806E-261 | Macro-VSIR | ZFP36 |
| 8.1501E-264 | 0.828268723 | 0.993 | 0.865 | 2.2801E-259 | Macro-VSIR | HLA-DPB1 |
| 5.7863E-261 | 0.844872979 | 1 | 0.914 | 1.6188E-256 | Macro-VSIR | CST3 |
| 1.4085E-257 | 0.759088147 | 0.816 | 0.485 | 3.9405E-253 | Macro-VSIR | VSIR |
| 2.3674E-254 | 0.754605609 | 0.999 | 0.923 | 6.6229E-250 | Macro-VSIR | HLA-DRA |
| 9.4456E-243 | 1.521268939 | 0.562 | 0.243 | 2.6425E-238 | Macro-VSIR | AREG |
| 4.5001E-239 | 1.11276011 | 0.817 | 0.501 | 1.2589E-234 | Macro-VSIR | GPR183 |
| 3.7655E-234 | 0.810050926 | 0.793 | 0.463 | 1.0534E-229 | Macro-VSIR | CPVL |
| 2.35E-229 | 0.525807895 | 1 | 0.957 | 6.5743E-225 | Macro-VSIR | RPL10 |
| 4.6064E-224 | 0.604074231 | 0.552 | 0.243 | 1.2887E-219 | Macro-VSIR | AOAH |
| 5.1586E-223 | 0.68649608 | 0.304 | 0.075 | 1.4432E-218 | Macro-VSIR | CFP |
| 3.8865E-221 | 0.744842635 | 0.998 | 0.896 | 1.0873E-216 | Macro-VSIR | HLA-DRB1 |
| 2.167E-218 | 0.841317728 | 0.968 | 0.793 | 6.0623E-214 | Macro-VSIR | JUNB |
| 3.2569E-216 | 0.754225682 | 0.531 | 0.223 | 9.1116E-212 | Macro-VSIR | LGALS2 |
| 6.6648E-206 | 0.89170166 | 0.84 | 0.542 | 1.8645E-201 | Macro-VSIR | IL1B |
| 9.6477E-206 | 0.60173043 | 0.874 | 0.549 | 2.6991E-201 | Macro-VSIR | FGL2 |
| 3.228E-204 | 0.481355661 | 0.321 | 0.093 | 9.0307E-200 | Macro-VSIR | CEACAM4 |
| 2.5439E-202 | 0.65743233 | 0.995 | 0.879 | 7.1167E-198 | Macro-VSIR | HLA-DPA1 |
| 9.2273E-198 | 0.754810711 | 0.768 | 0.44 | 2.5814E-193 | Macro-VSIR | FCGR2B |
| 3.7308E-197 | 0.769614197 | 0.641 | 0.331 | 1.0437E-192 | Macro-VSIR | NR4A3 |
| 6.1939E-196 | 0.72169651 | 0.972 | 0.762 | 1.7328E-191 | Macro-VSIR | LYZ |
| 1.9179E-192 | 0.50571126 | 0.994 | 0.905 | 5.3656E-188 | Macro-VSIR | RPS9 |
| 4.3482E-191 | 0.504878064 | 0.29 | 0.079 | 1.2165E-186 | Macro-VSIR | NRG1 |
| 1.8152E-190 | 0.949185873 | 0.826 | 0.558 | 5.0783E-186 | Macro-VSIR | DUSP2 |
| 4.5541E-186 | 0.664871007 | 0.895 | 0.6 | 1.274E-181 | Macro-VSIR | NR4A2 |
| 9.4233E-185 | 0.60058149 | 0.715 | 0.421 | 2.6363E-180 | Macro-VSIR | ALDH2 |
| 1.0113E-177 | 0.755224761 | 0.877 | 0.622 | 2.8292E-173 | Macro-VSIR | ZFP36L2 |
| 9.8488E-177 | 0.473994913 | 0.999 | 0.967 | 2.7553E-172 | Macro-VSIR | EEF1A1 |
| 1.3901E-173 | 0.742062887 | 0.9 | 0.651 | 3.8888E-169 | Macro-VSIR | TNFAIP3 |
| 2.779E-172 | 0.572553907 | 0.683 | 0.397 | 7.7745E-168 | Macro-VSIR | LILRB2 |
| 7.8281E-172 | 0.450559948 | 0.997 | 0.92 | 2.19E-167 | Macro-VSIR | RPS24 |
| 1.0421E-168 | 0.557872214 | 0.968 | 0.749 | 2.9155E-164 | Macro-VSIR | HLA-DMA |
| 4.9169E-168 | 0.740347329 | 0.683 | 0.378 | 1.3755E-163 | Macro-VSIR | RBKS |
| 2.5229E-167 | 0.590380215 | 0.768 | 0.505 | 7.0582E-163 | Macro-VSIR | IFNGR1 |
| 2.8927E-167 | 0.622016072 | 0.885 | 0.606 | 8.0926E-163 | Macro-VSIR | NFKBIZ |
| 9.2253E-167 | 0.615155864 | 0.839 | 0.568 | 2.5809E-162 | Macro-VSIR | C1orf162 |
| 1.6499E-162 | 0.708971373 | 0.961 | 0.774 | 4.6158E-158 | Macro-VSIR | HLA-DQB1 |
| 1.6643E-161 | 0.582664143 | 0.637 | 0.352 | 4.6559E-157 | Macro-VSIR | CSRNP1 |
| 1.0929E-160 | 0.669081978 | 0.861 | 0.6 | 3.0575E-156 | Macro-VSIR | NR4A1 |
| 3.5927E-158 | 0.682524015 | 0.957 | 0.765 | 1.0051E-153 | Macro-VSIR | HLA-DQA1 |
| 9.8217E-158 | 0.613063769 | 0.472 | 0.216 | 2.7477E-153 | Macro-VSIR | ARL5B |
| 2.3182E-157 | 0.529666775 | 0.533 | 0.262 | 6.4853E-153 | Macro-VSIR | GRASP |
| 4.4665E-157 | 0.427621121 | 0.997 | 0.922 | 1.2495E-152 | Macro-VSIR | RPL11 |
| 5.1537E-157 | 0.417532544 | 0.999 | 0.944 | 1.4418E-152 | Macro-VSIR | RPL13 |
| 8.5804E-153 | 0.703316994 | 0.976 | 0.856 | 2.4005E-148 | Macro-VSIR | NFKBIA |
| 5.3683E-152 | 0.480917529 | 0.585 | 0.316 | 1.5018E-147 | Macro-VSIR | STK17B |
| 4.1313E-150 | 0.564751004 | 0.981 | 0.851 | 1.1558E-145 | Macro-VSIR | DUSP1 |
| 0 | 3.6647118 | 0.361 | 0.052 | 0 | Macro-VEGFA | CXCL5 |
| 0 | 2.200327324 | 0.533 | 0.122 | 0 | Macro-VEGFA | INHBA |
| 0 | 2.190890977 | 0.765 | 0.327 | 0 | Macro-VEGFA | G0S2 |
| 0 | 1.965514078 | 0.981 | 0.819 | 0 | Macro-VEGFA | SOD2 |
| 0 | 1.825777032 | 0.906 | 0.546 | 0 | Macro-VEGFA | C15orf48 |
| 0 | 1.727798134 | 0.942 | 0.72 | 0 | Macro-VEGFA | PLAUR |
| 0 | 1.721590852 | 0.898 | 0.656 | 0 | Macro-VEGFA | CD44 |
| 0 | 1.594906052 | 0.999 | 0.997 | 0 | Macro-VEGFA | FTH1 |
| 0 | 1.489492903 | 0.308 | 0.045 | 0 | Macro-VEGFA | TNIP3 |
| 1.3225E-298 | 1.946445927 | 0.744 | 0.388 | 3.6999E-294 | Macro-VEGFA | SLC2A3 |
| 1.4544E-295 | 2.046671451 | 0.401 | 0.095 | 4.0687E-291 | Macro-VEGFA | TNFAIP6 |
| 9.0597E-275 | 2.747102482 | 0.502 | 0.16 | 2.5345E-270 | Macro-VEGFA | CCL20 |
| 1.5314E-257 | 2.131041524 | 0.692 | 0.34 | 4.2841E-253 | Macro-VEGFA | IL1RN |
| 6.8951E-253 | 1.301673779 | 0.61 | 0.271 | 1.929E-248 | Macro-VEGFA | TREM1 |
| 3.6847E-248 | 1.184884619 | 0.425 | 0.125 | 1.0308E-243 | Macro-VEGFA | AQP9 |
| 2.6663E-237 | 1.622371816 | 0.353 | 0.089 | 7.4593E-233 | Macro-VEGFA | HIF1A-AS2 |
| 2.8201E-236 | 2.325271702 | 0.863 | 0.626 | 7.8895E-232 | Macro-VEGFA | CXCL8 |
| 2.2314E-229 | 3.408329944 | 0.841 | 0.644 | 6.2426E-225 | Macro-VEGFA | TIMP1 |
| 2.3283E-220 | 0.977232239 | 0.304 | 0.068 | 6.5136E-216 | Macro-VEGFA | CD300E |
| 1.3469E-218 | 1.562481914 | 0.251 | 0.047 | 3.768E-214 | Macro-VEGFA | HILPDA |
| 4.5498E-217 | 1.532227059 | 0.49 | 0.195 | 1.2729E-212 | Macro-VEGFA | SLC25A37 |
| 6.0155E-216 | 1.569196267 | 0.712 | 0.372 | 1.6829E-211 | Macro-VEGFA | S100A8 |
| 2.6711E-207 | 1.792601447 | 0.788 | 0.569 | 7.4727E-203 | Macro-VEGFA | PLIN2 |
| 9.7585E-206 | 1.235329971 | 0.381 | 0.118 | 2.7301E-201 | Macro-VEGFA | EHD1 |
| 1.2127E-203 | 0.957623751 | 0.989 | 0.963 | 3.3927E-199 | Macro-VEGFA | SAT1 |
| 2.921E-202 | 1.22652414 | 0.736 | 0.466 | 8.1717E-198 | Macro-VEGFA | UPP1 |
| 7.2502E-200 | 1.037901608 | 0.767 | 0.466 | 2.0283E-195 | Macro-VEGFA | SLC11A1 |
| 1.1102E-193 | 1.072704715 | 0.393 | 0.132 | 3.1058E-189 | Macro-VEGFA | HK2 |
| 4.0852E-193 | 1.074425503 | 0.894 | 0.624 | 1.1429E-188 | Macro-VEGFA | BCL2A1 |
| 1.1625E-190 | 1.347763342 | 0.491 | 0.201 | 3.2521E-186 | Macro-VEGFA | ADM |
| 1.3371E-184 | 2.373755416 | 0.598 | 0.317 | 3.7406E-180 | Macro-VEGFA | PTGS2 |
| 4.2815E-178 | 1.153247082 | 0.932 | 0.838 | 1.1978E-173 | Macro-VEGFA | CSTB |
| 5.2926E-178 | 0.862301418 | 0.269 | 0.065 | 1.4807E-173 | Macro-VEGFA | SLAMF9 |
| 1.4142E-174 | 1.87542173 | 0.791 | 0.562 | 3.9563E-170 | Macro-VEGFA | IL1B |
| 9.8043E-165 | 0.793727418 | 0.973 | 0.938 | 2.7429E-160 | Macro-VEGFA | GAPDH |
| 2.9282E-163 | 1.071251284 | 0.52 | 0.254 | 8.192E-159 | Macro-VEGFA | NDRG1 |
| 4.1812E-147 | 0.970571774 | 0.854 | 0.676 | 1.1697E-142 | Macro-VEGFA | BTG1 |
| 1.7155E-143 | 1.067932828 | 0.693 | 0.453 | 4.7992E-139 | Macro-VEGFA | GK |
| 3.0392E-141 | 0.896716192 | 0.381 | 0.151 | 8.5026E-137 | Macro-VEGFA | IL3RA |
| 1.2957E-140 | 0.994706171 | 0.549 | 0.315 | 3.6248E-136 | Macro-VEGFA | TNIP1 |
| 1.9782E-139 | 1.367560634 | 0.43 | 0.177 | 5.5344E-135 | Macro-VEGFA | EREG |
| 3.3474E-139 | 1.268491939 | 0.672 | 0.435 | 9.3648E-135 | Macro-VEGFA | PPIF |
| 1.9153E-138 | 1.058534534 | 0.58 | 0.334 | 5.3583E-134 | Macro-VEGFA | BASP1 |
| 3.1421E-137 | 0.955836043 | 0.533 | 0.264 | 8.7904E-133 | Macro-VEGFA | VEGFA |
| 1.7903E-134 | 0.917906884 | 0.76 | 0.529 | 5.0084E-130 | Macro-VEGFA | FLNA |
| 2.4954E-133 | 0.856707854 | 0.289 | 0.093 | 6.9811E-129 | Macro-VEGFA | DUSP4 |
| 9.0739E-133 | 0.899329294 | 0.568 | 0.33 | 2.5385E-128 | Macro-VEGFA | ATP13A3 |
| 6.7184E-132 | 1.170062661 | 0.39 | 0.16 | 1.8795E-127 | Macro-VEGFA | VCAN |
| 2.1759E-128 | 0.988816377 | 0.858 | 0.701 | 6.0874E-124 | Macro-VEGFA | NAMPT |
| 6.2402E-128 | 0.949353437 | 0.539 | 0.295 | 1.7458E-123 | Macro-VEGFA | ANPEP |
| 0 | 3.462864924 | 0.836 | 0.382 | 0 | Macro-MKI67 | HIST1H4C |
| 0 | 2.897932707 | 0.931 | 0.292 | 0 | Macro-MKI67 | STMN1 |
| 0 | 2.431059852 | 0.96 | 0.644 | 0 | Macro-MKI67 | HMGN2 |
| 0 | 2.365603848 | 0.682 | 0.095 | 0 | Macro-MKI67 | PTTG1 |
| 0 | 2.322693309 | 0.662 | 0.014 | 0 | Macro-MKI67 | UBE2C |
| 0 | 2.260169625 | 0.768 | 0.018 | 0 | Macro-MKI67 | TYMS |
| 0 | 2.233374831 | 0.633 | 0.013 | 0 | Macro-MKI67 | TOP2A |
| 0 | 2.194971474 | 0.972 | 0.639 | 0 | Macro-MKI67 | H2AFZ |
| 0 | 2.181187888 | 0.983 | 0.682 | 0 | Macro-MKI67 | TUBB |
| 0 | 2.042017058 | 0.994 | 0.8 | 0 | Macro-MKI67 | TUBA1B |
| 0 | 1.965352447 | 0.679 | 0.013 | 0 | Macro-MKI67 | MKI67 |
| 0 | 1.933222974 | 0.855 | 0.351 | 0 | Macro-MKI67 | HMGB2 |
| 0 | 1.922950938 | 0.63 | 0.022 | 0 | Macro-MKI67 | CENPF |
| 0 | 1.896599316 | 0.647 | 0.028 | 0 | Macro-MKI67 | NUSAP1 |
| 0 | 1.826513774 | 0.968 | 0.758 | 0 | Macro-MKI67 | HMGB1 |
| 0 | 1.79255222 | 0.693 | 0.107 | 0 | Macro-MKI67 | SMC4 |
| 0 | 1.645460546 | 0.76 | 0.106 | 0 | Macro-MKI67 | CKS1B |
| 0 | 1.598510274 | 0.744 | 0.041 | 0 | Macro-MKI67 | TK1 |
| 0 | 1.565692003 | 0.657 | 0.034 | 0 | Macro-MKI67 | CDK1 |
| 0 | 1.45496615 | 0.599 | 0.017 | 0 | Macro-MKI67 | TPX2 |
| 0 | 1.420298191 | 0.718 | 0.214 | 0 | Macro-MKI67 | H2AFX |
| 0 | 1.402490485 | 0.695 | 0.186 | 0 | Macro-MKI67 | KPNA2 |
| 0 | 1.364681085 | 0.658 | 0.08 | 0 | Macro-MKI67 | LMNB1 |
| 0 | 1.363263784 | 0.925 | 0.602 | 0 | Macro-MKI67 | DEK |
| 0 | 1.349946478 | 0.538 | 0.011 | 0 | Macro-MKI67 | CDC20 |
| 0 | 1.34531067 | 0.487 | 0.022 | 0 | Macro-MKI67 | CCNB1 |
| 0 | 1.344272246 | 0.792 | 0.327 | 0 | Macro-MKI67 | DUT |
| 0 | 1.342253321 | 0.908 | 0.53 | 0 | Macro-MKI67 | H2AFV |
| 0 | 1.337601032 | 0.687 | 0.098 | 0 | Macro-MKI67 | CENPW |
| 0 | 1.327312471 | 0.59 | 0.013 | 0 | Macro-MKI67 | BIRC5 |
| 0 | 1.268302906 | 0.437 | 0.018 | 0 | Macro-MKI67 | HIST1H1B |
| 0 | 1.265487547 | 0.382 | 0.006 | 0 | Macro-MKI67 | HIST1H3B |
| 0 | 1.26185883 | 0.647 | 0.116 | 0 | Macro-MKI67 | HMGB3 |
| 0 | 1.253241385 | 0.524 | 0.014 | 0 | Macro-MKI67 | KIFC1 |
| 0 | 1.246580161 | 0.671 | 0.076 | 0 | Macro-MKI67 | MCM7 |
| 0 | 1.237546082 | 0.928 | 0.614 | 0 | Macro-MKI67 | RAN |
| 0 | 1.237230793 | 0.648 | 0.017 | 0 | Macro-MKI67 | PCLAF |
| 0 | 1.232236817 | 0.732 | 0.192 | 0 | Macro-MKI67 | TMPO |
| 0 | 1.232207253 | 0.581 | 0.021 | 0 | Macro-MKI67 | CDKN3 |
| 0 | 1.228269961 | 0.631 | 0.014 | 0 | Macro-MKI67 | CENPM |
| 0 | 1.210482552 | 0.823 | 0.352 | 0 | Macro-MKI67 | RANBP1 |
| 0 | 1.168130934 | 0.704 | 0.132 | 0 | Macro-MKI67 | DTYMK |
| 0 | 1.164493127 | 0.641 | 0.09 | 0 | Macro-MKI67 | SMC2 |
| 0 | 1.152736289 | 0.651 | 0.131 | 0 | Macro-MKI67 | PCNA |
| 0 | 1.151881789 | 0.547 | 0.023 | 0 | Macro-MKI67 | ASF1B |
| 0 | 1.144440389 | 0.589 | 0.048 | 0 | Macro-MKI67 | ATAD2 |
| 0 | 1.114514846 | 0.54 | 0.058 | 0 | Macro-MKI67 | GGH |
| 0 | 1.114308769 | 0.548 | 0.015 | 0 | Macro-MKI67 | CCNB2 |
| 0 | 1.092760614 | 0.765 | 0.241 | 0 | Macro-MKI67 | RPA3 |
| 0 | 1.072710567 | 0.611 | 0.076 | 0 | Macro-MKI67 | TMEM106C |
| 3.0238E-159 | 3.195122915 | 1 | 0.929 | 8.4595E-155 | Macro-NEAT1 | MALAT1 |
| 1.213E-151 | 3.417168287 | 0.99 | 0.85 | 3.3935E-147 | Macro-NEAT1 | NEAT1 |
| 2.98127E-89 | 2.187105725 | 0.886 | 0.754 | 8.34041E-85 | Macro-NEAT1 | MCL1 |
| 7.76928E-68 | 2.447400473 | 0.759 | 0.544 | 2.17353E-63 | Macro-NEAT1 | CCNL1 |
| 4.84557E-66 | 2.069914258 | 0.466 | 0.154 | 1.3556E-61 | Macro-NEAT1 | AC016831.7 |
| 7.30281E-61 | 2.094359659 | 0.869 | 0.685 | 2.04303E-56 | Macro-NEAT1 | TNFAIP3 |
| 5.94767E-56 | 2.088139742 | 0.766 | 0.618 | 1.66392E-51 | Macro-NEAT1 | REL |
| 5.9146E-55 | 1.775515802 | 0.831 | 0.716 | 1.65467E-50 | Macro-NEAT1 | NAMPT |
| 7.50744E-54 | 2.012150218 | 0.814 | 0.644 | 2.10028E-49 | Macro-NEAT1 | NFKBIZ |
| 1.56687E-52 | 2.100733859 | 0.834 | 0.806 | 4.38348E-48 | Macro-NEAT1 | HNRNPA2B1 |
| 1.72908E-52 | 1.775519152 | 0.855 | 0.728 | 4.83727E-48 | Macro-NEAT1 | CD83 |
| 1.6575E-51 | 2.180327272 | 0.669 | 0.463 | 4.63702E-47 | Macro-NEAT1 | RSRP1 |
| 6.57783E-50 | 1.912633432 | 0.724 | 0.604 | 1.84021E-45 | Macro-NEAT1 | FUS |
| 6.34849E-49 | 1.295707444 | 0.914 | 0.836 | 1.77605E-44 | Macro-NEAT1 | SOD2 |
| 1.01673E-45 | 1.867176601 | 0.717 | 0.602 | 2.84439E-41 | Macro-NEAT1 | WSB1 |
| 4.7121E-43 | 1.859807543 | 0.669 | 0.463 | 1.31826E-38 | Macro-NEAT1 | KDM6B |
| 7.01697E-43 | 2.096132443 | 0.576 | 0.342 | 1.96307E-38 | Macro-NEAT1 | NFKBID |
| 3.92905E-40 | 1.723868032 | 0.79 | 0.636 | 1.09919E-35 | Macro-NEAT1 | NR4A1 |
| 4.66656E-40 | 2.131091742 | 0.717 | 0.602 | 1.30552E-35 | Macro-NEAT1 | ICAM1 |
| 6.10069E-40 | 2.245802042 | 0.566 | 0.352 | 1.70673E-35 | Macro-NEAT1 | NFKB1 |
| 2.47936E-37 | 2.344820719 | 0.555 | 0.357 | 6.93627E-33 | Macro-NEAT1 | NABP1 |
| 4.0063E-37 | 1.651822044 | 0.769 | 0.642 | 1.1208E-32 | Macro-NEAT1 | NR4A2 |
| 1.05411E-35 | 1.260643215 | 0.862 | 0.758 | 2.94899E-31 | Macro-NEAT1 | PPP1R15A |
| 3.6342E-35 | 2.144613102 | 0.603 | 0.45 | 1.0167E-30 | Macro-NEAT1 | RASGEF1B |
| 5.59885E-35 | 2.066155516 | 0.503 | 0.291 | 1.56633E-30 | Macro-NEAT1 | IFRD1 |
| 1.0425E-33 | 2.030706335 | 0.776 | 0.789 | 2.91651E-29 | Macro-NEAT1 | SQSTM1 |
| 1.11072E-33 | 2.202167459 | 0.569 | 0.389 | 3.10736E-29 | Macro-NEAT1 | ABL2 |
| 3.50473E-32 | 1.678508999 | 0.638 | 0.539 | 9.80482E-28 | Macro-NEAT1 | RBM39 |
| 3.76943E-32 | 1.336269128 | 0.807 | 0.695 | 1.05454E-27 | Macro-NEAT1 | FOSB |
| 1.08886E-31 | 1.998471319 | 0.548 | 0.381 | 3.0462E-27 | Macro-NEAT1 | ZNF267 |
| 4.55934E-31 | 1.551745044 | 0.659 | 0.585 | 1.27552E-26 | Macro-NEAT1 | AKAP13 |
| 2.34181E-30 | 2.172610289 | 0.552 | 0.377 | 6.55144E-26 | Macro-NEAT1 | PPP1R10 |
| 2.96995E-30 | 1.868002552 | 0.359 | 0.158 | 8.30873E-26 | Macro-NEAT1 | AC007384.1 |
| 9.11009E-30 | 1.506014864 | 0.283 | 0.098 | 2.54864E-25 | Macro-NEAT1 | DCN |
| 4.38063E-29 | 2.367177801 | 0.255 | 0.083 | 1.22552E-24 | Macro-NEAT1 | TTN |
| 8.64791E-29 | 2.349397725 | 0.69 | 0.607 | 2.41934E-24 | Macro-NEAT1 | HSPH1 |
| 2.93558E-28 | 1.055139955 | 0.841 | 0.844 | 8.21257E-24 | Macro-NEAT1 | MT-ND4L |
| 3.97683E-28 | 2.001482558 | 0.428 | 0.225 | 1.11256E-23 | Macro-NEAT1 | AC020916.1 |
| 1.6847E-26 | 1.972401693 | 0.683 | 0.672 | 4.71311E-22 | Macro-NEAT1 | HNRNPU |
| 6.61536E-26 | 0.849220415 | 0.952 | 0.954 | 1.85071E-21 | Macro-NEAT1 | MT-CO1 |
| 8.17442E-25 | 1.656135618 | 0.621 | 0.564 | 2.28688E-20 | Macro-NEAT1 | CFLAR |
| 1.26622E-24 | 2.133691311 | 0.603 | 0.564 | 3.54239E-20 | Macro-NEAT1 | DNAJB6 |
| 2.5143E-24 | 1.069991013 | 0.762 | 0.781 | 7.034E-20 | Macro-NEAT1 | DDX5 |
| 6.69025E-24 | 1.463055014 | 0.776 | 0.785 | 1.87167E-19 | Macro-NEAT1 | KLF6 |
| 1.33536E-23 | 0.88920015 | 0.872 | 0.898 | 3.7358E-19 | Macro-NEAT1 | MT-CYB |
| 1.84628E-23 | 1.945239777 | 0.559 | 0.465 | 5.16515E-19 | Macro-NEAT1 | B4GALT1 |
| 7.53461E-23 | 1.630662275 | 0.693 | 0.622 | 2.10788E-18 | Macro-NEAT1 | BTG2 |
| 1.13862E-22 | 1.430313921 | 0.641 | 0.614 | 3.18539E-18 | Macro-NEAT1 | ZEB2 |
| 6.50346E-22 | 1.820224918 | 0.583 | 0.525 | 1.81941E-17 | Macro-NEAT1 | ABCA1 |
| 1.19137E-21 | 1.520450075 | 0.61 | 0.528 | 3.33298E-17 | Macro-NEAT1 | TNFAIP2 |

**Feature genes expressed by T cells subsets of our in house ESCC scRNA-seq**

| p_val | avg_log2FC | pct.1 | pct.2 | p_val_adj | cluster | gene |
| --- | --- | --- | --- | --- | --- | --- |
| 0 | 2.528110086 | 0.9 | 0.183 | 0 | CD8-GZMK | GZMK |
| 0 | 2.17203966 | 0.539 | 0.173 | 0 | CD8-GZMK | CCL4L2 |
| 0 | 1.723291235 | 0.525 | 0.152 | 0 | CD8-GZMK | TNFSF9 |
| 0 | 1.664316736 | 0.883 | 0.38 | 0 | CD8-GZMK | CCL4 |
| 0 | 1.288091005 | 0.345 | 0.077 | 0 | CD8-GZMK | CRTAM |
| 0 | 1.257129055 | 0.462 | 0.187 | 0 | CD8-GZMK | ITM2C |
| 0 | 1.217512099 | 0.72 | 0.278 | 0 | CD8-GZMK | CD8A |
| 0 | 1.191549717 | 0.935 | 0.579 | 0 | CD8-GZMK | CST7 |
| 0 | 1.126174035 | 0.915 | 0.689 | 0 | CD8-GZMK | DUSP2 |
| 0 | 1.088522894 | 0.578 | 0.222 | 0 | CD8-GZMK | CD8B |
| 0 | 1.035678643 | 0.976 | 0.489 | 0 | CD8-GZMK | CCL5 |
| 0 | 1.01886247 | 0.444 | 0.206 | 0 | CD8-GZMK | CMC1 |
| 0 | 0.879340179 | 0.693 | 0.441 | 0 | CD8-GZMK | HLA-DPB1 |
| 0 | 0.871937989 | 0.682 | 0.402 | 0 | CD8-GZMK | HLA-DRB1 |
| 0 | 0.863194619 | 0.467 | 0.263 | 0 | CD8-GZMK | HLA-DRA |
| 0 | 0.837132001 | 0.543 | 0.277 | 0 | CD8-GZMK | GZMH |
| 0 | 0.826218343 | 0.536 | 0.381 | 0 | CD8-GZMK | CLDND1 |
| 0 | 0.810262115 | 0.429 | 0.202 | 0 | CD8-GZMK | HLA-DQA1 |
| 0 | 0.805759165 | 0.953 | 0.836 | 0 | CD8-GZMK | CD74 |
| 0 | 0.797753757 | 0.625 | 0.39 | 0 | CD8-GZMK | GZMM |
| 0 | 0.782350265 | 0.702 | 0.465 | 0 | CD8-GZMK | HLA-DPA1 |
| 0 | 0.768434763 | 0.748 | 0.558 | 0 | CD8-GZMK | COTL1 |
| 0 | 0.754109682 | 0.537 | 0.33 | 0 | CD8-GZMK | APOBEC3G |
| 0 | 0.747999177 | 0.443 | 0.254 | 0 | CD8-GZMK | SH2D1A |
| 0 | 0.741601477 | 0.894 | 0.414 | 0 | CD8-GZMK | NKG7 |
| 0 | 0.704472959 | 0.444 | 0.251 | 0 | CD8-GZMK | HLA-DRB5 |
| 0 | 0.685449512 | 0.266 | 0.105 | 0 | CD8-GZMK | KLRG1 |
| 0 | 0.680396632 | 0.287 | 0.106 | 0 | CD8-GZMK | PLEK |
| 0 | 0.677009246 | 0.945 | 0.842 | 0 | CD8-GZMK | CXCR4 |
| 0 | 0.64664393 | 0.289 | 0.121 | 0 | CD8-GZMK | AOAH |
| 0 | 2.82571662 | 0.655 | 0.112 | 0 | CD4-Foxp3 | TNFRSF4 |
| 0 | 2.647684796 | 0.687 | 0.033 | 0 | CD4-Foxp3 | FOXP3 |
| 0 | 2.169829934 | 0.816 | 0.278 | 0 | CD4-Foxp3 | BATF |
| 0 | 1.944417245 | 0.652 | 0.178 | 0 | CD4-Foxp3 | TNFRSF18 |
| 0 | 1.913859288 | 0.493 | 0.056 | 0 | CD4-Foxp3 | IL2RA |
| 0 | 1.663814351 | 0.76 | 0.231 | 0 | CD4-Foxp3 | CTLA4 |
| 0 | 1.644031354 | 0.826 | 0.359 | 0 | CD4-Foxp3 | LTB |
| 0 | 1.633582847 | 0.456 | 0.068 | 0 | CD4-Foxp3 | LAIR2 |
| 0 | 1.599450853 | 0.743 | 0.301 | 0 | CD4-Foxp3 | CARD16 |
| 0 | 1.475561062 | 0.859 | 0.562 | 0 | CD4-Foxp3 | SAT1 |
| 0 | 1.421668427 | 0.517 | 0.091 | 0 | CD4-Foxp3 | TBC1D4 |
| 0 | 1.372730207 | 0.322 | 0.012 | 0 | CD4-Foxp3 | IL1R2 |
| 0 | 1.359400658 | 0.641 | 0.228 | 0 | CD4-Foxp3 | DNPH1 |
| 0 | 1.339866739 | 0.751 | 0.307 | 0 | CD4-Foxp3 | TIGIT |
| 0 | 1.322698755 | 0.364 | 0.066 | 0 | CD4-Foxp3 | MAGEH1 |
| 0 | 1.309293678 | 0.401 | 0.071 | 0 | CD4-Foxp3 | LINC01943 |
| 0 | 1.285567044 | 0.987 | 0.881 | 0 | CD4-Foxp3 | IL32 |
| 0 | 1.273164729 | 0.73 | 0.337 | 0 | CD4-Foxp3 | DUSP4 |
| 0 | 1.231212263 | 0.449 | 0.086 | 0 | CD4-Foxp3 | GK |
| 0 | 1.21523966 | 0.532 | 0.13 | 0 | CD4-Foxp3 | STAM |
| 0 | 1.211181136 | 0.596 | 0.25 | 0 | CD4-Foxp3 | PIM2 |
| 0 | 1.193556098 | 0.315 | 0.023 | 0 | CD4-Foxp3 | RTKN2 |
| 0 | 1.180513829 | 0.318 | 0.01 | 0 | CD4-Foxp3 | CCR8 |
| 0 | 1.179352764 | 0.632 | 0.281 | 0 | CD4-Foxp3 | CORO1B |
| 0 | 1.16009829 | 0.514 | 0.171 | 0 | CD4-Foxp3 | MIR4435-2HG |
| 0 | 1.15842348 | 0.376 | 0.059 | 0 | CD4-Foxp3 | LAYN |
| 0 | 1.149351787 | 0.306 | 0.085 | 0 | CD4-Foxp3 | HPGD |
| 0 | 1.147521298 | 0.73 | 0.332 | 0 | CD4-Foxp3 | CD27 |
| 0 | 1.114430561 | 0.639 | 0.277 | 0 | CD4-Foxp3 | ICOS |
| 0 | 1.106235418 | 0.581 | 0.311 | 0 | CD4-Foxp3 | SYNGR2 |
| 0 | 1.517293463 | 0.533 | 0.146 | 0 | CD4-CCR7 | CCR7 |
| 0 | 1.183731068 | 0.917 | 0.534 | 0 | CD4-CCR7 | IL7R |
| 0 | 1.116730727 | 0.516 | 0.255 | 0 | CD4-CCR7 | KLF2 |
| 0 | 1.040537791 | 0.669 | 0.341 | 0 | CD4-CCR7 | GPR183 |
| 0 | 0.988377088 | 0.411 | 0.113 | 0 | CD4-CCR7 | TCF7 |
| 0 | 0.960748446 | 0.732 | 0.492 | 0 | CD4-CCR7 | SLC2A3 |
| 0 | 0.923855662 | 0.963 | 0.831 | 0 | CD4-CCR7 | EEF1B2 |
| 0 | 0.919251927 | 0.29 | 0.059 | 0 | CD4-CCR7 | CD40LG |
| 0 | 0.906075461 | 0.961 | 0.846 | 0 | CD4-CCR7 | PABPC1 |
| 0 | 0.901746901 | 0.997 | 0.954 | 0 | CD4-CCR7 | RPS3A |
| 0 | 0.891512105 | 1 | 0.971 | 0 | CD4-CCR7 | RPL32 |
| 0 | 0.860412592 | 0.998 | 0.969 | 0 | CD4-CCR7 | RPS8 |
| 0 | 0.838317016 | 0.999 | 0.965 | 0 | CD4-CCR7 | RPL34 |
| 0 | 0.829059668 | 0.998 | 0.959 | 0 | CD4-CCR7 | RPS13 |
| 0 | 0.827343618 | 0.347 | 0.107 | 0 | CD4-CCR7 | LEF1 |
| 0 | 0.821967041 | 1 | 0.977 | 0 | CD4-CCR7 | RPS12 |
| 0 | 0.820231082 | 0.823 | 0.654 | 0 | CD4-CCR7 | EEF1G |
| 0 | 0.803547653 | 0.996 | 0.949 | 0 | CD4-CCR7 | RPS6 |
| 0 | 0.802160681 | 0.997 | 0.963 | 0 | CD4-CCR7 | TPT1 |
| 0 | 0.800840307 | 0.967 | 0.846 | 0 | CD4-CCR7 | JUNB |
| 0 | 0.78285907 | 0.995 | 0.95 | 0 | CD4-CCR7 | RPL3 |
| 0 | 0.775299245 | 0.998 | 0.966 | 0 | CD4-CCR7 | RPS18 |
| 0 | 0.770501318 | 1 | 0.989 | 0 | CD4-CCR7 | EEF1A1 |
| 0 | 0.769061874 | 0.99 | 0.93 | 0 | CD4-CCR7 | RPL5 |
| 0 | 0.76880605 | 0.999 | 0.97 | 0 | CD4-CCR7 | RPL11 |
| 0 | 0.766891331 | 0.99 | 0.928 | 0 | CD4-CCR7 | RPS5 |
| 0 | 0.762678482 | 0.968 | 0.866 | 0 | CD4-CCR7 | RPL22 |
| 0 | 0.749782765 | 0.981 | 0.894 | 0 | CD4-CCR7 | RPL9 |
| 0 | 0.742649183 | 0.999 | 0.968 | 0 | CD4-CCR7 | RPL39 |
| 0 | 0.732605992 | 0.629 | 0.37 | 0 | CD4-CCR7 | PTGER4 |
| 0 | 1.364276334 | 0.96 | 0.998 | 0 | uncharaterized | MALAT1 |
| 1.5776E-263 | 0.749936819 | 0.95 | 0.996 | 4.4136E-259 | uncharaterized | MT-CO1 |
| 7.8781E-202 | 0.663898975 | 0.938 | 0.994 | 2.204E-197 | uncharaterized | MT-CO2 |
| 1.3518E-199 | 0.77565033 | 0.861 | 0.938 | 3.7819E-195 | uncharaterized | MT-CYB |
| 6.2519E-171 | 0.723946452 | 0.865 | 0.94 | 1.749E-166 | uncharaterized | MT-CO3 |
| 2.5992E-170 | 0.760653761 | 0.832 | 0.936 | 7.2715E-166 | uncharaterized | MT-ND4L |
| 1.9084E-137 | 0.305705802 | 0.213 | 0.496 | 5.3389E-133 | uncharaterized | TUBB4B |
| 7.4824E-121 | 0.776713924 | 0.752 | 0.862 | 2.0933E-116 | uncharaterized | MT-ND1 |
| 9.8199E-118 | 0.717783115 | 0.788 | 0.909 | 2.7472E-113 | uncharaterized | MT-ND3 |
| 3.00271E-93 | 0.610271449 | 0.853 | 0.958 | 8.40037E-89 | uncharaterized | MT-ATP6 |
| 3.6651E-92 | 0.782763667 | 0.702 | 0.81 | 1.02535E-87 | uncharaterized | MT-ND2 |
| 1.12686E-70 | 0.718394256 | 0.68 | 0.796 | 3.15251E-66 | uncharaterized | MT-ND5 |
| 5.8019E-60 | 0.618654666 | 0.722 | 0.865 | 1.62314E-55 | uncharaterized | MT-ND4 |
| 3.07714E-52 | 1.595603639 | 0.498 | 0.544 | 8.6086E-48 | uncharaterized | NEAT1 |
| 1.34165E-46 | 0.306720548 | 0.454 | 0.707 | 3.75341E-42 | uncharaterized | CD69 |
| 3.04451E-41 | 0.567311883 | 0.323 | 0.542 | 8.51733E-37 | uncharaterized | IER2 |
| 1.97473E-40 | 0.42331074 | 0.243 | 0.422 | 5.52449E-36 | uncharaterized | PDE4B |
| 1.30335E-38 | 0.282866289 | 0.191 | 0.34 | 3.64624E-34 | uncharaterized | STAT4 |
| 2.64204E-38 | 0.252880912 | 0.197 | 0.348 | 7.39137E-34 | uncharaterized | NOP58 |
| 4.48698E-38 | 0.255475698 | 0.196 | 0.347 | 1.25528E-33 | uncharaterized | GPBP1 |
| 7.76091E-38 | 0.356342188 | 0.268 | 0.455 | 2.17119E-33 | uncharaterized | RNF19A |
| 8.21052E-38 | 0.303372155 | 0.198 | 0.337 | 2.29697E-33 | uncharaterized | CTLA4 |
| 1.15109E-37 | 0.305575094 | 0.143 | 0.263 | 3.22029E-33 | uncharaterized | RNF125 |
| 2.83482E-36 | 0.260586413 | 0.217 | 0.365 | 7.93068E-32 | uncharaterized | PRDM1 |
| 3.05828E-36 | 0.287347277 | 0.209 | 0.366 | 8.55585E-32 | uncharaterized | CLK1 |
| 3.87514E-36 | 0.256715955 | 0.294 | 0.497 | 1.08411E-31 | uncharaterized | HSP90B1 |
| 3.84249E-35 | 0.31998257 | 0.309 | 0.524 | 1.07497E-30 | uncharaterized | STAT3 |
| 7.18325E-34 | 0.269036802 | 0.202 | 0.349 | 2.00959E-29 | uncharaterized | CANX |
| 7.29216E-34 | 0.255369022 | 0.167 | 0.293 | 2.04005E-29 | uncharaterized | PTPN1 |
| 1.43746E-33 | 0.267733569 | 0.223 | 0.379 | 4.02144E-29 | uncharaterized | ADAR |
| 0 | 1.565880988 | 0.413 | 0.101 | 0 | CD8-XCL1 | XCL1 |
| 0 | 1.237429315 | 0.28 | 0.026 | 0 | CD8-XCL1 | FXYD2 |
| 0 | 1.175079081 | 0.885 | 0.513 | 0 | CD8-XCL1 | ANXA1 |
| 0 | 1.122650145 | 0.327 | 0.099 | 0 | CD8-XCL1 | LINC02446 |
| 0 | 1.059192258 | 0.896 | 0.687 | 0 | CD8-XCL1 | S100A10 |
| 0 | 1.023510524 | 0.545 | 0.193 | 0 | CD8-XCL1 | HOPX |
| 0 | 1.012727862 | 0.757 | 0.327 | 0 | CD8-XCL1 | CD8A |
| 0 | 1.011230964 | 0.643 | 0.258 | 0 | CD8-XCL1 | CD8B |
| 0 | 0.916005561 | 0.991 | 0.546 | 0 | CD8-XCL1 | CCL5 |
| 0 | 0.881969046 | 0.394 | 0.145 | 0 | CD8-XCL1 | XCL2 |
| 0 | 0.851818933 | 0.98 | 0.871 | 0 | CD8-XCL1 | VIM |
| 0 | 0.712129246 | 0.98 | 0.889 | 0 | CD8-XCL1 | ZFP36L2 |
| 0 | 0.695879142 | 0.493 | 0.202 | 0 | CD8-XCL1 | KLRD1 |
| 0 | 0.658603187 | 0.683 | 0.342 | 0 | CD8-XCL1 | CTSW |
| 0 | 0.653984271 | 0.935 | 0.767 | 0 | CD8-XCL1 | IFITM2 |
| 0 | 0.477323405 | 0.693 | 0.329 | 0 | CD8-XCL1 | GZMB |
| 8.7616E-303 | 0.782900088 | 0.929 | 0.788 | 2.4512E-298 | CD8-XCL1 | S100A6 |
| 1.3256E-300 | 1.115510503 | 0.593 | 0.333 | 3.7086E-296 | CD8-XCL1 | LMNA |
| 2.527E-295 | 0.797687525 | 0.472 | 0.219 | 7.0695E-291 | CD8-XCL1 | CAPG |
| 1.6313E-261 | 0.638829474 | 0.356 | 0.142 | 4.5638E-257 | CD8-XCL1 | MATK |
| 7.864E-258 | 0.547293951 | 0.775 | 0.451 | 2.2E-253 | CD8-XCL1 | CCL4 |
| 1.8744E-255 | 0.724513842 | 0.613 | 0.359 | 5.2438E-251 | CD8-XCL1 | CD63 |
| 7.6404E-254 | 0.605204788 | 0.958 | 0.861 | 2.1375E-249 | CD8-XCL1 | ZFP36 |
| 6.3806E-253 | 0.905767472 | 0.653 | 0.404 | 1.785E-248 | CD8-XCL1 | LGALS1 |
| 6.8076E-249 | 0.790391231 | 0.254 | 0.084 | 1.9045E-244 | CD8-XCL1 | KLRC1 |
| 1.0598E-244 | 0.758366096 | 0.406 | 0.187 | 2.9649E-240 | CD8-XCL1 | GLUL |
| 5.0537E-242 | 0.730021788 | 0.635 | 0.385 | 1.4138E-237 | CD8-XCL1 | MYADM |
| 4.5762E-235 | 0.540298597 | 0.893 | 0.686 | 1.2802E-230 | CD8-XCL1 | HCST |
| 2.8219E-231 | 0.531052998 | 0.964 | 0.853 | 7.8946E-227 | CD8-XCL1 | CXCR4 |
| 5.592E-225 | 0.639472978 | 0.594 | 0.355 | 1.5644E-220 | CD8-XCL1 | PARP8 |
| 0 | 3.209152671 | 0.733 | 0.014 | 0 | CD8-CX3CR1 | FGFBP2 |
| 0 | 2.745800502 | 0.604 | 0.021 | 0 | CD8-CX3CR1 | FCGR3A |
| 0 | 2.473165775 | 0.937 | 0.377 | 0 | CD8-CX3CR1 | PRF1 |
| 0 | 2.447479697 | 0.996 | 0.467 | 0 | CD8-CX3CR1 | NKG7 |
| 0 | 2.259723204 | 0.566 | 0.099 | 0 | CD8-CX3CR1 | SPON2 |
| 0 | 2.120762181 | 0.518 | 0.098 | 0 | CD8-CX3CR1 | TYROBP |
| 0 | 2.086099004 | 0.814 | 0.178 | 0 | CD8-CX3CR1 | KLRD1 |
| 0 | 2.013896375 | 0.857 | 0.188 | 0 | CD8-CX3CR1 | GNLY |
| 0 | 1.975611024 | 0.903 | 0.28 | 0 | CD8-CX3CR1 | GZMH |
| 0 | 1.639894492 | 0.928 | 0.441 | 0 | CD8-CX3CR1 | CCL4 |
| 0 | 1.625632502 | 0.488 | 0.049 | 0 | CD8-CX3CR1 | ADGRG1 |
| 0 | 1.610838762 | 0.815 | 0.334 | 0 | CD8-CX3CR1 | CTSW |
| 0 | 1.58942926 | 0.935 | 0.312 | 0 | CD8-CX3CR1 | GZMB |
| 0 | 1.586105823 | 0.304 | 0.096 | 0 | CD8-CX3CR1 | CCL3 |
| 0 | 1.573393119 | 0.363 | 0.016 | 0 | CD8-CX3CR1 | KLRF1 |
| 0 | 1.542579614 | 0.383 | 0.008 | 0 | CD8-CX3CR1 | CX3CR1 |
| 0 | 1.47972008 | 0.976 | 0.622 | 0 | CD8-CX3CR1 | CST7 |
| 0 | 1.474841648 | 0.382 | 0.011 | 0 | CD8-CX3CR1 | S1PR5 |
| 0 | 1.454304731 | 0.44 | 0.134 | 0 | CD8-CX3CR1 | CLIC3 |
| 0 | 1.440873845 | 0.545 | 0.107 | 0 | CD8-CX3CR1 | PLEK |
| 0 | 1.281906271 | 0.416 | 0.072 | 0 | CD8-CX3CR1 | PLAC8 |
| 0 | 1.280728058 | 0.538 | 0.208 | 0 | CD8-CX3CR1 | EFHD2 |
| 0 | 1.267612395 | 0.325 | 0.01 | 0 | CD8-CX3CR1 | PRSS23 |
| 0 | 1.214573256 | 0.604 | 0.191 | 0 | CD8-CX3CR1 | HOPX |
| 0 | 1.201164421 | 0.524 | 0.163 | 0 | CD8-CX3CR1 | ZEB2 |
| 0 | 1.141964471 | 0.785 | 0.417 | 0 | CD8-CX3CR1 | LITAF |
| 0 | 1.136791323 | 0.746 | 0.44 | 0 | CD8-CX3CR1 | FLNA |
| 0 | 1.135938426 | 0.828 | 0.549 | 0 | CD8-CX3CR1 | ITGB2 |
| 0 | 1.086173285 | 0.332 | 0.037 | 0 | CD8-CX3CR1 | FCRL6 |
| 0 | 1.057032427 | 0.301 | 0.046 | 0 | CD8-CX3CR1 | TTC38 |
| 0 | 2.445340032 | 0.638 | 0.059 | 0 | CD8-LAG3 | CXCL13 |
| 0 | 2.23056857 | 0.946 | 0.319 | 0 | CD8-LAG3 | GZMB |
| 0 | 1.962791665 | 0.349 | 0.037 | 0 | CD8-LAG3 | KRT86 |
| 0 | 1.640844972 | 0.6 | 0.215 | 0 | CD8-LAG3 | GNLY |
| 0 | 1.627250764 | 0.739 | 0.232 | 0 | CD8-LAG3 | ITGAE |
| 0 | 1.575417783 | 0.724 | 0.226 | 0 | CD8-LAG3 | LAG3 |
| 0 | 1.523483974 | 0.88 | 0.462 | 0 | CD8-LAG3 | GZMA |
| 0 | 1.497681063 | 0.918 | 0.325 | 0 | CD8-LAG3 | CD8A |
| 0 | 1.423744265 | 0.38 | 0.08 | 0 | CD8-LAG3 | CSF1 |
| 0 | 1.422563651 | 0.416 | 0.065 | 0 | CD8-LAG3 | PLPP1 |
| 0 | 1.399609403 | 0.374 | 0.047 | 0 | CD8-LAG3 | KIR2DL4 |
| 0 | 1.345470594 | 0.461 | 0.086 | 0 | CD8-LAG3 | HAVCR2 |
| 0 | 1.33104461 | 0.848 | 0.488 | 0 | CD8-LAG3 | ALOX5AP |
| 0 | 1.326479739 | 0.376 | 0.079 | 0 | CD8-LAG3 | KLRC1 |
| 0 | 1.322281233 | 0.601 | 0.2 | 0 | CD8-LAG3 | ACP5 |
| 0 | 1.287341597 | 0.965 | 0.558 | 0 | CD8-LAG3 | CCL5 |
| 0 | 1.261928501 | 0.541 | 0.144 | 0 | CD8-LAG3 | LINC01871 |
| 0 | 1.241941455 | 0.629 | 0.307 | 0 | CD8-LAG3 | RGS2 |
| 0 | 1.225910858 | 0.283 | 0.076 | 0 | CD8-LAG3 | SOX4 |
| 0 | 1.221629449 | 0.625 | 0.226 | 0 | CD8-LAG3 | CXCR6 |
| 0 | 1.215624217 | 0.397 | 0.084 | 0 | CD8-LAG3 | GALNT2 |
| 0 | 1.199868075 | 0.966 | 0.711 | 0 | CD8-LAG3 | CD7 |
| 0 | 1.186472635 | 0.772 | 0.258 | 0 | CD8-LAG3 | CD8B |
| 0 | 1.176743714 | 0.669 | 0.283 | 0 | CD8-LAG3 | RBPJ |
| 0 | 1.170201586 | 0.457 | 0.118 | 0 | CD8-LAG3 | ENTPD1 |
| 0 | 1.161930878 | 0.905 | 0.594 | 0 | CD8-LAG3 | RGS1 |
| 0 | 1.145415403 | 0.372 | 0.063 | 0 | CD8-LAG3 | GPR25 |
| 0 | 1.126084338 | 0.285 | 0.023 | 0 | CD8-LAG3 | MYO1E |
| 0 | 1.10839927 | 0.529 | 0.17 | 0 | CD8-LAG3 | PTMS |
| 0 | 1.082929822 | 0.69 | 0.356 | 0 | CD8-LAG3 | PHLDA1 |
| 0 | 1.633421568 | 0.723 | 0.241 | 0 | CD4-RORA | KLRB1 |
| 0 | 1.273095422 | 0.422 | 0.059 | 0 | CD4-RORA | CTSH |
| 0 | 1.185551351 | 0.268 | 0.049 | 0 | CD4-RORA | CCL20 |
| 0 | 1.008516624 | 0.301 | 0.03 | 0 | CD4-RORA | PTPN13 |
| 0 | 0.911122397 | 0.337 | 0.072 | 0 | CD4-RORA | FURIN |
| 0 | 0.889683048 | 0.355 | 0.076 | 0 | CD4-RORA | ADAM19 |
| 4.0432E-279 | 0.77420113 | 0.298 | 0.068 | 1.1311E-274 | CD4-RORA | GNA15 |
| 1.2669E-251 | 0.799251328 | 0.329 | 0.086 | 3.5442E-247 | CD4-RORA | CD40LG |
| 5.933E-247 | 0.823854093 | 0.465 | 0.159 | 1.6598E-242 | CD4-RORA | TNFRSF25 |
| 1.7669E-230 | 0.771848846 | 0.979 | 0.787 | 4.943E-226 | CD4-RORA | S100A4 |
| 3.2695E-221 | 0.980932241 | 0.569 | 0.24 | 9.1468E-217 | CD4-RORA | CXCR6 |
| 4.4993E-218 | 0.832930748 | 0.875 | 0.558 | 1.2587E-213 | CD4-RORA | SPOCK2 |
| 8.2247E-218 | 0.840310599 | 0.399 | 0.131 | 2.3009E-213 | CD4-RORA | CCR6 |
| 1.8693E-206 | 0.6364886 | 0.281 | 0.076 | 5.2296E-202 | CD4-RORA | ARL3 |
| 9.0278E-206 | 0.834612957 | 0.605 | 0.272 | 2.5256E-201 | CD4-RORA | PDE4D |
| 2.0729E-204 | 2.339926028 | 0.301 | 0.089 | 5.7991E-200 | CD4-RORA | CXCL13 |
| 5.1786E-204 | 0.857252563 | 0.685 | 0.35 | 1.4488E-199 | CD4-RORA | RORA |
| 1.0586E-181 | 0.72933412 | 0.269 | 0.076 | 2.9614E-177 | CD4-RORA | GPR25 |
| 5.0408E-181 | 0.660006786 | 0.473 | 0.186 | 1.4102E-176 | CD4-RORA | CD4 |
| 5.5194E-177 | 0.822293158 | 0.454 | 0.183 | 1.5441E-172 | CD4-RORA | PDCD1 |
| 1.8363E-163 | 0.588723845 | 0.312 | 0.102 | 5.1371E-159 | CD4-RORA | RUNX2 |
| 4.7067E-162 | 0.789257153 | 0.595 | 0.297 | 1.3167E-157 | CD4-RORA | RBPJ |
| 5.6324E-162 | 0.672591954 | 0.409 | 0.158 | 1.5757E-157 | CD4-RORA | JAML |
| 8.31E-158 | 0.684775762 | 0.932 | 0.773 | 2.3248E-153 | CD4-RORA | LAPTM5 |
| 2.4346E-154 | 0.742083421 | 0.513 | 0.236 | 6.8111E-150 | CD4-RORA | SNX9 |
| 1.2242E-148 | 0.65079935 | 0.416 | 0.169 | 3.4247E-144 | CD4-RORA | MAF |
| 1.7191E-139 | 0.733311931 | 0.662 | 0.388 | 4.8092E-135 | CD4-RORA | OSTF1 |
| 1.0175E-136 | 0.673237948 | 0.89 | 0.656 | 2.8465E-132 | CD4-RORA | S100A11 |
| 5.435E-132 | 0.772443694 | 0.844 | 0.606 | 1.5205E-127 | CD4-RORA | RGS1 |
| 8.5989E-131 | 0.67361175 | 0.568 | 0.295 | 2.4056E-126 | CD4-RORA | CD82 |
| 0 | 3.394792263 | 0.863 | 0.269 | 0 | NKT | AREG |
| 0 | 2.791404737 | 0.734 | 0.221 | 0 | NKT | GNLY |
| 0 | 2.723530697 | 0.774 | 0.039 | 0 | NKT | FCER1G |
| 0 | 2.687944053 | 0.916 | 0.099 | 0 | NKT | TYROBP |
| 0 | 2.668937098 | 0.632 | 0.108 | 0 | NKT | XCL1 |
| 0 | 2.281309318 | 0.634 | 0.148 | 0 | NKT | XCL2 |
| 0 | 2.275049075 | 0.593 | 0.035 | 0 | NKT | TRDC |
| 0 | 2.213705437 | 0.61 | 0.078 | 0 | NKT | KLRC1 |
| 0 | 1.839485147 | 0.482 | 0.041 | 0 | NKT | KRT86 |
| 0 | 1.727226921 | 0.379 | 0.013 | 0 | NKT | KRT81 |
| 0 | 1.680549577 | 0.677 | 0.289 | 0 | NKT | NR4A1 |
| 0 | 1.673279046 | 0.523 | 0.043 | 0 | NKT | TMIGD2 |
| 0 | 1.584591234 | 0.679 | 0.233 | 0 | NKT | MAP3K8 |
| 0 | 1.581113744 | 0.739 | 0.24 | 0 | NKT | KLRB1 |
| 0 | 1.569419682 | 0.443 | 0.053 | 0 | NKT | KIR2DL4 |
| 0 | 1.547078694 | 0.689 | 0.209 | 0 | NKT | KLRD1 |
| 0 | 1.495906882 | 0.782 | 0.355 | 0 | NKT | CTSW |
| 0 | 1.338669415 | 0.399 | 0.018 | 0 | NKT | SH2D1B |
| 0 | 1.317079348 | 0.522 | 0.122 | 0 | NKT | SLC16A3 |
| 0 | 1.31449289 | 0.624 | 0.207 | 0 | NKT | HOPX |
| 0 | 1.287888026 | 0.587 | 0.215 | 0 | NKT | IFITM3 |
| 0 | 1.28047047 | 0.673 | 0.279 | 0 | NKT | BHLHE40 |
| 0 | 1.260219529 | 0.494 | 0.091 | 0 | NKT | TXK |
| 0 | 1.180080007 | 0.386 | 0.08 | 0 | NKT | MAFF |
| 0 | 1.157599863 | 0.509 | 0.146 | 0 | NKT | MATK |
| 0 | 1.153372625 | 0.404 | 0.047 | 0 | NKT | LAT2 |
| 0 | 1.153357889 | 0.618 | 0.226 | 0 | NKT | CAPG |
| 0 | 1.149550245 | 0.287 | 0.007 | 0 | NKT | B3GNT7 |
| 0 | 1.081070189 | 0.76 | 0.366 | 0 | NKT | CD63 |
| 0 | 1.044754097 | 0.258 | 0.003 | 0 | NKT | ADGRG3 |
| 0 | 3.635666431 | 0.609 | 0.083 | 0 | CD4-CXCL13 | CXCL13 |
| 0 | 1.989451171 | 0.397 | 0.045 | 0 | CD4-CXCL13 | NMB |
| 0 | 1.809058146 | 0.759 | 0.262 | 0 | CD4-CXCL13 | IL6ST |
| 0 | 1.613302201 | 0.502 | 0.075 | 0 | CD4-CXCL13 | TOX2 |
| 0 | 1.547391598 | 0.897 | 0.426 | 0 | CD4-CXCL13 | NR3C1 |
| 0 | 1.476552757 | 0.368 | 0.032 | 0 | CD4-CXCL13 | PTPN13 |
| 0 | 1.450490011 | 0.471 | 0.048 | 0 | CD4-CXCL13 | SMCO4 |
| 0 | 1.363416673 | 0.478 | 0.067 | 0 | CD4-CXCL13 | ICA1 |
| 0 | 1.282912133 | 0.546 | 0.149 | 0 | CD4-CXCL13 | TCF7 |
| 0 | 1.246401517 | 0.542 | 0.117 | 0 | CD4-CXCL13 | TNFSF8 |
| 0 | 1.216332051 | 0.346 | 0.014 | 0 | CD4-CXCL13 | DRAIC |
| 0 | 1.199795643 | 0.459 | 0.08 | 0 | CD4-CXCL13 | TSHZ2 |
| 0 | 1.192332256 | 0.316 | 0.023 | 0 | CD4-CXCL13 | CD200 |
| 0 | 1.001816235 | 0.292 | 0.032 | 0 | CD4-CXCL13 | ARMH1 |
| 0 | 0.962367486 | 0.261 | 0.015 | 0 | CD4-CXCL13 | CPM |
| 0 | 0.952888822 | 0.329 | 0.048 | 0 | CD4-CXCL13 | BTLA |
| 0 | 0.873371025 | 0.271 | 0.039 | 0 | CD4-CXCL13 | AC012645.3 |
| 0 | 0.833884631 | 0.26 | 0.021 | 0 | CD4-CXCL13 | AC004585.1 |
| 6.1818E-274 | 1.034289137 | 0.297 | 0.055 | 1.7294E-269 | CD4-CXCL13 | NUDT16 |
| 1.9286E-272 | 1.083807113 | 0.577 | 0.181 | 5.3955E-268 | CD4-CXCL13 | TOX |
| 1.5053E-265 | 1.11887838 | 0.486 | 0.134 | 4.2112E-261 | CD4-CXCL13 | SESN3 |
| 1.7665E-264 | 1.57567071 | 0.438 | 0.117 | 4.942E-260 | CD4-CXCL13 | SESN1 |
| 3.6653E-258 | 0.906690332 | 0.298 | 0.056 | 1.0254E-253 | CD4-CXCL13 | IGFBP4 |
| 1.0148E-242 | 1.435633884 | 0.797 | 0.407 | 2.839E-238 | CD4-CXCL13 | FKBP5 |
| 2.864E-242 | 1.034029631 | 0.374 | 0.088 | 8.0123E-238 | CD4-CXCL13 | CD40LG |
| 6.5878E-240 | 0.892672522 | 0.407 | 0.103 | 1.843E-235 | CD4-CXCL13 | FBLN7 |
| 1.2746E-234 | 0.975258956 | 0.418 | 0.11 | 3.5659E-230 | CD4-CXCL13 | MAGEH1 |
| 5.8671E-229 | 0.794615637 | 0.331 | 0.073 | 1.6414E-224 | CD4-CXCL13 | IL6R |
| 9.786E-224 | 1.083995173 | 0.552 | 0.184 | 2.7377E-219 | CD4-CXCL13 | PDCD1 |
| 3.4385E-215 | 0.892466084 | 0.383 | 0.1 | 9.6195E-211 | CD4-CXCL13 | TIAM1 |
| 0 | 2.719801661 | 0.929 | 0.326 | 0 | CD8-ISG15 | ISG15 |
| 0 | 2.4674848 | 0.844 | 0.206 | 0 | CD8-ISG15 | MX1 |
| 0 | 2.422978999 | 0.876 | 0.265 | 0 | CD8-ISG15 | IFI6 |
| 0 | 2.266298789 | 0.605 | 0.027 | 0 | CD8-ISG15 | IFIT1 |
| 0 | 2.002151835 | 0.467 | 0.026 | 0 | CD8-ISG15 | IFIT3 |
| 0 | 1.971089445 | 0.386 | 0.025 | 0 | CD8-ISG15 | IFIT2 |
| 0 | 1.883317177 | 0.598 | 0.074 | 0 | CD8-ISG15 | IFI44L |
| 0 | 1.875411835 | 0.649 | 0.12 | 0 | CD8-ISG15 | OAS1 |
| 0 | 1.873628785 | 0.519 | 0.03 | 0 | CD8-ISG15 | RSAD2 |
| 0 | 1.85055455 | 0.647 | 0.121 | 0 | CD8-ISG15 | MX2 |
| 0 | 1.815402518 | 0.954 | 0.629 | 0 | CD8-ISG15 | ISG20 |
| 0 | 1.809039224 | 0.622 | 0.165 | 0 | CD8-ISG15 | OASL |
| 0 | 1.726497399 | 0.739 | 0.225 | 0 | CD8-ISG15 | IRF7 |
| 0 | 1.720918901 | 0.963 | 0.588 | 0 | CD8-ISG15 | LY6E |
| 0 | 1.696426724 | 1 | 0.872 | 0 | CD8-ISG15 | IFITM1 |
| 0 | 1.567184125 | 0.541 | 0.118 | 0 | CD8-ISG15 | HERC5 |
| 0 | 1.558572115 | 0.679 | 0.176 | 0 | CD8-ISG15 | XAF1 |
| 0 | 1.552263419 | 0.669 | 0.18 | 0 | CD8-ISG15 | EIF2AK2 |
| 0 | 1.546199323 | 0.549 | 0.129 | 0 | CD8-ISG15 | PLSCR1 |
| 0 | 1.493782879 | 0.454 | 0.069 | 0 | CD8-ISG15 | OAS3 |
| 0 | 1.454014782 | 0.448 | 0.035 | 0 | CD8-ISG15 | CMPK2 |
| 0 | 1.250209772 | 0.371 | 0.039 | 0 | CD8-ISG15 | USP18 |
| 0 | 1.07006835 | 0.27 | 0.017 | 0 | CD8-ISG15 | LAMP3 |
| 1.1574E-285 | 1.524811895 | 0.618 | 0.18 | 3.238E-281 | CD8-ISG15 | IFI35 |
| 4.9004E-280 | 1.524244338 | 0.678 | 0.217 | 1.3709E-275 | CD8-ISG15 | EPSTI1 |
| 5.7256E-270 | 1.399541558 | 0.568 | 0.152 | 1.6018E-265 | CD8-ISG15 | SAMD9L |
| 9.5436E-261 | 1.614169342 | 0.776 | 0.324 | 2.6699E-256 | CD8-ISG15 | STAT1 |
| 1.7434E-255 | 1.248693408 | 0.463 | 0.107 | 4.8774E-251 | CD8-ISG15 | LGALS9 |
| 8.2753E-249 | 1.265841778 | 0.445 | 0.101 | 2.3151E-244 | CD8-ISG15 | IFI44 |
| 1.0831E-237 | 1.524978836 | 0.791 | 0.372 | 3.0301E-233 | CD8-ISG15 | TRIM22 |
| 0 | 2.794562059 | 0.891 | 0.147 | 0 | CD8-MKI67 | STMN1 |
| 0 | 2.657650095 | 0.787 | 0.01 | 0 | CD8-MKI67 | TYMS |
| 0 | 1.913354044 | 0.795 | 0.063 | 0 | CD8-MKI67 | MCM7 |
| 0 | 1.800856171 | 0.799 | 0.098 | 0 | CD8-MKI67 | MCM5 |
| 0 | 1.665675807 | 0.833 | 0.282 | 0 | CD8-MKI67 | DUT |
| 0 | 1.517772891 | 0.501 | 0.012 | 0 | CD8-MKI67 | TK1 |
| 0 | 1.468840875 | 0.681 | 0.098 | 0 | CD8-MKI67 | PCNA |
| 0 | 1.464557396 | 0.642 | 0.017 | 0 | CD8-MKI67 | MCM2 |
| 0 | 1.458361319 | 0.518 | 0.007 | 0 | CD8-MKI67 | PCLAF |
| 0 | 1.45753128 | 0.473 | 0.016 | 0 | CD8-MKI67 | MKI67 |
| 0 | 1.426483846 | 0.727 | 0.107 | 0 | CD8-MKI67 | MCM3 |
| 0 | 1.320782184 | 0.548 | 0.011 | 0 | CD8-MKI67 | GINS2 |
| 0 | 1.306079727 | 0.842 | 0.259 | 0 | CD8-MKI67 | RANBP1 |
| 0 | 1.268585248 | 0.551 | 0.023 | 0 | CD8-MKI67 | MCM4 |
| 0 | 1.264658382 | 0.521 | 0.01 | 0 | CD8-MKI67 | CLSPN |
| 0 | 1.18240021 | 0.634 | 0.095 | 0 | CD8-MKI67 | MCM6 |
| 0 | 1.175361016 | 0.608 | 0.095 | 0 | CD8-MKI67 | TMEM106C |
| 0 | 1.17494395 | 0.543 | 0.048 | 0 | CD8-MKI67 | HELLS |
| 0 | 1.123888835 | 0.509 | 0.035 | 0 | CD8-MKI67 | FEN1 |
| 0 | 1.123103642 | 0.465 | 0.018 | 0 | CD8-MKI67 | DHFR |
| 0 | 1.118126382 | 0.682 | 0.152 | 0 | CD8-MKI67 | CENPX |
| 0 | 1.107178931 | 0.416 | 0.002 | 0 | CD8-MKI67 | UHRF1 |
| 0 | 1.093893705 | 0.421 | 0.009 | 0 | CD8-MKI67 | ASF1B |
| 0 | 1.088160355 | 0.468 | 0.005 | 0 | CD8-MKI67 | CDT1 |
| 0 | 1.086542892 | 0.367 | 0.028 | 0 | CD8-MKI67 | NUSAP1 |
| 0 | 1.032097627 | 0.396 | 0.008 | 0 | CD8-MKI67 | ZWINT |
| 0 | 1.017596368 | 0.468 | 0.037 | 0 | CD8-MKI67 | SMC2 |
| 0 | 1.002367048 | 0.444 | 0.058 | 0 | CD8-MKI67 | CENPM |
| 0 | 0.995193098 | 0.262 | 0.016 | 0 | CD8-MKI67 | TOP2A |
| 0 | 0.951882687 | 0.282 | 0.027 | 0 | CD8-MKI67 | CENPF |

**Differently expessed genes between Macro-LY6E and Macro-C1QC**

| gene | p_val | avg_log2FC | pct.1 | pct.2 | p_val_adj |
| --- | --- | --- | --- | --- | --- |
| ATP5F1E | 0 | -4.067411812 | 0 | 0.994 | 0 |
| RACK1 | 0 | -3.260251455 | 0 | 0.977 | 0 |
| CCL3L1 | 5.51E-228 | -3.126524643 | 0 | 0.58 | 1.67E-223 |
| ATP5MC2 | 0 | -2.860624269 | 0 | 0.952 | 0 |
| ATP5MG | 0 | -2.812087889 | 0 | 0.966 | 0 |
| ATP5MC3 | 0 | -2.671243071 | 0 | 0.923 | 0 |
| ELOB | 0 | -2.572350218 | 0 | 0.947 | 0 |
| ATP5F1B | 0 | -2.319717484 | 0 | 0.902 | 0 |
| MTRNR2L12 | 4.98E-20 | -2.289402045 | 0.051 | 0.152 | 1.51E-15 |
| ATP5MF | 0 | -2.289326372 | 0 | 0.912 | 0 |
| ATP5MD | 0 | -2.192105886 | 0 | 0.911 | 0 |
| SELENOP | 5.49E-126 | -2.134813667 | 0 | 0.379 | 1.67E-121 |
| FYB1 | 0 | -2.045419573 | 0 | 0.843 | 0 |
| ATP5PD | 0 | -2.035561484 | 0 | 0.907 | 0 |
| SPP1 | 1.36E-103 | -2.019923525 | 0.412 | 0.729 | 4.12E-99 |
| SELENOH | 0 | -1.995400383 | 0 | 0.885 | 0 |
| ATP5F1D | 0 | -1.985681629 | 0 | 0.879 | 0 |
| JPT1 | 0 | -1.930998835 | 0 | 0.836 | 0 |
| CHI3L1 | 1.04E-32 | -1.887042342 | 0.08 | 0.241 | 3.15E-28 |
| SELENOW | 0 | -1.847147464 | 0 | 0.832 | 0 |
| ATP5PF | 0 | -1.844152669 | 0 | 0.866 | 0 |
| ATP5MPL | 0 | -1.792890354 | 0 | 0.846 | 0 |
| CALHM6 | 0 | -1.787930823 | 0 | 0.714 | 0 |
| SEM1 | 0 | -1.674322474 | 0 | 0.849 | 0 |
| ATP5F1C | 0 | -1.673447177 | 0 | 0.822 | 0 |
| ATP5ME | 0 | -1.641580688 | 0 | 0.816 | 0 |
| RPL41 | 5.15E-265 | -1.619536737 | 0.937 | 0.986 | 1.57E-260 |
| VSIR | 0 | -1.608570687 | 0 | 0.724 | 0 |
| APOC1 | 4.91E-133 | -1.579201161 | 0.663 | 0.902 | 1.49E-128 |
| ELOC | 0 | -1.555874848 | 0 | 0.793 | 0 |
| SMIM25 | 1.15E-280 | -1.550482221 | 0 | 0.664 | 3.50E-276 |
| ATP5PB | 0 | -1.534171964 | 0 | 0.787 | 0 |
| LIPA | 2.55E-207 | -1.524185781 | 0.482 | 0.844 | 7.75E-203 |
| ATP5IF1 | 0 | -1.494279719 | 0 | 0.799 | 0 |
| TRIR | 0 | -1.491066384 | 0 | 0.809 | 0 |
| ADA2 | 0 | -1.48276339 | 0 | 0.754 | 0 |
| REX1BD | 0 | -1.473672455 | 0 | 0.801 | 0 |
| ATP5F1A | 0 | -1.466591575 | 0 | 0.759 | 0 |
| RAB5IF | 0 | -1.459805338 | 0 | 0.742 | 0 |
| LGMN | 5.27E-157 | -1.456743658 | 0.639 | 0.881 | 1.60E-152 |
| CXCL1 | 3.11E-46 | -1.424550873 | 0.191 | 0.415 | 9.44E-42 |
| APOE | 6.24E-117 | -1.410964052 | 0.608 | 0.883 | 1.89E-112 |
| SELENOK | 0 | -1.336420741 | 0 | 0.717 | 0 |
| GPNMB | 1.23E-178 | -1.330927672 | 0.504 | 0.866 | 3.73E-174 |
| CD81 | 1.30E-214 | -1.326681543 | 0.692 | 0.938 | 3.95E-210 |
| FBP1 | 9.27E-19 | -1.282181296 | 0.51 | 0.632 | 2.82E-14 |
| MMP12 | 1.54E-45 | -1.273778747 | 0.232 | 0.463 | 4.69E-41 |
| A2M | 1.04E-167 | -1.259214545 | 0.359 | 0.783 | 3.17E-163 |
| TREM2 | 1.06E-142 | -1.257781882 | 0.535 | 0.836 | 3.22E-138 |
| SQOR | 1.50E-296 | -1.25540544 | 0 | 0.687 | 4.55E-292 |
| SLC40A1 | 6.55E-79 | -1.248186842 | 0.277 | 0.572 | 1.99E-74 |
| SELENOT | 0 | -1.236113429 | 0 | 0.729 | 0 |
| SELENOF | 0 | -1.214433511 | 0 | 0.715 | 0 |
| CD9 | 4.16E-143 | -1.187441267 | 0.534 | 0.837 | 1.26E-138 |
| COPS9 | 3.81E-302 | -1.167778231 | 0 | 0.695 | 1.16E-297 |
| SELENOS | 2.26E-284 | -1.164565303 | 0 | 0.669 | 6.86E-280 |
| LSP1 | 1.98E-133 | -1.127873076 | 0.54 | 0.828 | 6.02E-129 |
| FABP5 | 4.51E-118 | -1.126488052 | 0.648 | 0.889 | 1.37E-113 |
| CTSZ | 1.54E-188 | -1.124350018 | 0.911 | 0.977 | 4.67E-184 |
| CXCL3 | 2.36E-60 | -1.120217796 | 0.401 | 0.645 | 7.16E-56 |
| SEPT2 | 5.71E-302 | -1.115589115 | 0 | 0.695 | 1.74E-297 |
| RTRAF | 1.35E-297 | -1.100263062 | 0 | 0.688 | 4.12E-293 |
| DAB2 | 1.12E-182 | -1.096297905 | 0.562 | 0.886 | 3.41E-178 |
| CTSB | 1.93E-214 | -1.090983329 | 0.987 | 0.999 | 5.87E-210 |
| SMIM26 | 8.03E-290 | -1.088025037 | 0 | 0.677 | 2.44E-285 |
| NECTIN2 | 1.34E-267 | -1.082655773 | 0 | 0.644 | 4.08E-263 |
| ATP5MC1 | 6.49E-255 | -1.072695753 | 0 | 0.624 | 1.97E-250 |
| PLD3 | 1.95E-139 | -1.070341453 | 0.768 | 0.912 | 5.93E-135 |
| SEPT7 | 7.83E-281 | -1.057281578 | 0 | 0.664 | 2.38E-276 |
| GSN | 2.27E-165 | -1.047351012 | 0.85 | 0.963 | 6.89E-161 |
| MARCKS | 6.04E-93 | -1.012930857 | 0.63 | 0.811 | 1.84E-88 |
| PLTP | 1.92E-104 | -0.998665246 | 0.368 | 0.725 | 5.83E-100 |
| STMP1 | 5.73E-261 | -0.988232158 | 0 | 0.634 | 1.74E-256 |
| GRN | 8.13E-183 | -0.978538869 | 0.943 | 0.982 | 2.47E-178 |
| 1-Mar | 8.48E-234 | -0.971473071 | 0 | 0.59 | 2.58E-229 |
| NUPR1 | 1.86E-67 | -0.970432884 | 0.163 | 0.453 | 5.66E-63 |
| MESD | 5.59E-251 | -0.965212821 | 0 | 0.618 | 1.70E-246 |
| MSR1 | 6.95E-141 | -0.959270204 | 0.424 | 0.794 | 2.11E-136 |
| TIMP2 | 1.10E-157 | -0.954814212 | 0.517 | 0.843 | 3.35E-153 |
| C1QB | 4.13E-98 | -0.946096375 | 0.77 | 0.924 | 1.26E-93 |
| SMIM37 | 3.30E-236 | -0.945374787 | 0 | 0.594 | 1.00E-231 |
| HLA-DRB5 | 1.61E-61 | -0.93402166 | 0.883 | 0.929 | 4.89E-57 |
| CXCL2 | 2.57E-50 | -0.929227049 | 0.496 | 0.699 | 7.82E-46 |
| CSTB | 8.44E-79 | -0.901236233 | 0.94 | 0.981 | 2.56E-74 |
| ANXA2 | 8.20E-156 | -0.89009697 | 0.947 | 0.98 | 2.49E-151 |
| SEPT9 | 4.57E-226 | -0.88947705 | 0 | 0.577 | 1.39E-221 |
| RNASE1 | 2.24E-44 | -0.880264989 | 0.51 | 0.744 | 6.81E-40 |
| MMP24OS | 9.15E-212 | -0.871384935 | 0 | 0.552 | 2.78E-207 |
| ACP5 | 1.69E-66 | -0.869113984 | 0.801 | 0.914 | 5.13E-62 |
| NOP53 | 9.60E-225 | -0.86184153 | 0 | 0.574 | 2.92E-220 |
| GNAS | 5.05E-125 | -0.856137789 | 0.75 | 0.912 | 1.53E-120 |
| NBDY | 5.06E-229 | -0.855984616 | 0 | 0.582 | 1.54E-224 |
| AL138963.3 | 5.32E-107 | -0.853849909 | 0 | 0.334 | 1.62E-102 |
| PLA2G7 | 4.67E-74 | -0.853700171 | 0.614 | 0.81 | 1.42E-69 |
| METTL26 | 2.28E-220 | -0.85287232 | 0 | 0.567 | 6.94E-216 |
| RTF2 | 3.54E-238 | -0.85097851 | 0 | 0.597 | 1.08E-233 |
| CYTOR | 1.47E-183 | -0.848535764 | 0 | 0.5 | 4.48E-179 |
| CCL3 | 1.32E-77 | -0.845650659 | 0.486 | 0.793 | 4.01E-73 |
| BEX3 | 1.64E-127 | -0.845166349 | 0 | 0.383 | 4.98E-123 |
| CYBC1 | 2.64E-224 | -0.841872079 | 0 | 0.574 | 8.03E-220 |
| GM2A | 2.43E-119 | -0.838200876 | 0.552 | 0.819 | 7.39E-115 |
| ADAP2 | 1.65E-118 | -0.833289132 | 0.442 | 0.761 | 5.02E-114 |
| RCAN1 | 1.82E-72 | -0.829048232 | 0.153 | 0.448 | 5.52E-68 |
| CAPG | 9.07E-164 | -0.82327482 | 0.952 | 0.982 | 2.76E-159 |
| CCL4L2 | 2.00E-96 | -0.821673094 | 0.335 | 0.683 | 6.09E-92 |
| FTL | 3.13E-172 | -0.819647996 | 1 | 1 | 9.52E-168 |
| MX1 | 5.41E-52 | -0.814414655 | 0.356 | 0.602 | 1.64E-47 |
| PSAP | 1.96E-136 | -0.813452349 | 0.983 | 0.998 | 5.97E-132 |
| NAXE | 9.36E-216 | -0.809190379 | 0 | 0.559 | 2.84E-211 |
| ZYX | 6.41E-139 | -0.807288285 | 0.736 | 0.902 | 1.95E-134 |
| C1QC | 2.38E-70 | -0.798628038 | 0.797 | 0.93 | 7.22E-66 |
| BCAP31 | 4.63E-141 | -0.795730394 | 0.77 | 0.905 | 1.41E-136 |
| GNAI2 | 4.28E-118 | -0.787645417 | 0.789 | 0.931 | 1.30E-113 |
| AHNAK | 1.20E-84 | -0.786191629 | 0.487 | 0.762 | 3.64E-80 |
| MS4A7 | 2.27E-88 | -0.783580914 | 0.863 | 0.945 | 6.90E-84 |
| WASHC4 | 4.77E-200 | -0.78283157 | 0 | 0.531 | 1.45E-195 |
| STAB1 | 4.59E-56 | -0.782321395 | 0.484 | 0.696 | 1.39E-51 |
| FCGRT | 6.26E-109 | -0.777244389 | 0.879 | 0.954 | 1.90E-104 |
| SLCO2B1 | 3.69E-99 | -0.775321275 | 0.49 | 0.78 | 1.12E-94 |
| SCARB2 | 4.99E-117 | -0.773194073 | 0.545 | 0.814 | 1.52E-112 |
| TUBA1B | 4.23E-80 | -0.772978407 | 0.893 | 0.945 | 1.29E-75 |
| ATP2B1-AS1 | 1.22E-126 | -0.771220695 | 0 | 0.381 | 3.71E-122 |
| HINT1 | 2.19E-148 | -0.767786649 | 0.804 | 0.92 | 6.65E-144 |
| ASAH1 | 2.48E-111 | -0.766329309 | 0.828 | 0.931 | 7.53E-107 |
| RTL8C | 4.11E-202 | -0.764578018 | 0 | 0.535 | 1.25E-197 |
| FOLR2 | 1.50E-40 | -0.762983658 | 0.229 | 0.448 | 4.56E-36 |
| MGST3 | 2.07E-122 | -0.758518942 | 0.728 | 0.877 | 6.29E-118 |
| MT-CO3 | 3.84E-77 | -0.75837807 | 0.972 | 0.988 | 1.17E-72 |
| CCL4 | 3.29E-60 | -0.756790812 | 0.521 | 0.765 | 9.99E-56 |
| LGALS3 | 1.35E-85 | -0.756373163 | 0.923 | 0.977 | 4.09E-81 |
| ANXA11 | 6.32E-118 | -0.755913346 | 0.66 | 0.848 | 1.92E-113 |
| ARL4C | 8.02E-47 | -0.755865676 | 0.481 | 0.681 | 2.44E-42 |
| GCHFR | 1.93E-73 | -0.754938445 | 0.243 | 0.543 | 5.86E-69 |
| SMIM29 | 1.34E-194 | -0.752313752 | 0 | 0.521 | 4.07E-190 |
| LINC02345 | 1.68E-100 | -0.747935622 | 0 | 0.318 | 5.09E-96 |
| VAT1 | 4.00E-108 | -0.742726113 | 0.323 | 0.678 | 1.22E-103 |
| RNF130 | 3.53E-144 | -0.740276481 | 0.849 | 0.945 | 1.07E-139 |
| CTSL | 3.21E-73 | -0.739712468 | 0.826 | 0.945 | 9.75E-69 |
| GRK2 | 7.20E-185 | -0.737092324 | 0 | 0.503 | 2.19E-180 |
| CD63 | 5.22E-143 | -0.730019497 | 0.97 | 0.991 | 1.59E-138 |
| BTG2 | 1.07E-58 | -0.729268792 | 0.465 | 0.72 | 3.26E-54 |
| CAMK1 | 1.07E-78 | -0.727322016 | 0.419 | 0.685 | 3.25E-74 |
| PLEK | 4.30E-78 | -0.725560171 | 0.655 | 0.871 | 1.31E-73 |
| AC020656.1 | 2.74E-120 | -0.725518974 | 0 | 0.366 | 8.33E-116 |
| CD99 | 2.94E-118 | -0.724196789 | 0.714 | 0.899 | 8.94E-114 |
| CREG1 | 4.86E-107 | -0.724130044 | 0.81 | 0.928 | 1.48E-102 |
| TMEM176B | 4.53E-70 | -0.720242248 | 0.827 | 0.912 | 1.38E-65 |
| LGALS9 | 4.07E-106 | -0.71750669 | 0.729 | 0.88 | 1.24E-101 |
| C1QA | 4.12E-74 | -0.716777532 | 0.789 | 0.936 | 1.25E-69 |
| MCRIP1 | 3.59E-188 | -0.71561443 | 0 | 0.509 | 1.09E-183 |
| SEPT6 | 2.99E-182 | -0.710640295 | 0 | 0.497 | 9.08E-178 |
| CENPX | 2.18E-174 | -0.70822865 | 0 | 0.482 | 6.61E-170 |
| SMIM30 | 3.34E-177 | -0.706934826 | 0 | 0.488 | 1.01E-172 |
| P4HB | 7.73E-117 | -0.706844638 | 0.792 | 0.913 | 2.35E-112 |
| MCUB | 1.47E-180 | -0.706396251 | 0 | 0.494 | 4.46E-176 |
| RPS18 | 1.03E-108 | -0.706377587 | 0.977 | 0.993 | 3.14E-104 |
| UFD1 | 2.56E-186 | -0.705512594 | 0 | 0.505 | 7.78E-182 |
| EGR1 | 2.82E-29 | -0.70534921 | 0.48 | 0.638 | 8.57E-25 |
| NRP1 | 3.43E-95 | -0.704994288 | 0.346 | 0.681 | 1.04E-90 |
| ZFP36 | 1.53E-66 | -0.704726744 | 0.802 | 0.937 | 4.65E-62 |
| TMEM35B | 5.01E-175 | -0.704365526 | 0 | 0.483 | 1.52E-170 |
| CTSA | 9.41E-94 | -0.702639781 | 0.777 | 0.886 | 2.86E-89 |
| ATP6AP2 | 4.45E-108 | -0.699113904 | 0.707 | 0.869 | 1.35E-103 |
| C9orf16 | 3.75E-105 | -0.697931777 | 0.63 | 0.841 | 1.14E-100 |
| APLP2 | 9.61E-92 | -0.696356084 | 0.676 | 0.868 | 2.92E-87 |
| SCPEP1 | 7.94E-91 | -0.695120599 | 0.538 | 0.752 | 2.41E-86 |
| TTYH3 | 7.37E-105 | -0.694562803 | 0.364 | 0.707 | 2.24E-100 |
| GPR34 | 8.10E-84 | -0.692166981 | 0.262 | 0.587 | 2.46E-79 |
| IFIT1 | 2.69E-31 | -0.691009379 | 0.127 | 0.302 | 8.17E-27 |
| PRDX1 | 3.28E-106 | -0.688807139 | 0.946 | 0.97 | 9.96E-102 |
| NDUFAF8 | 3.13E-164 | -0.687358961 | 0 | 0.462 | 9.52E-160 |
| FERMT3 | 4.53E-110 | -0.685037247 | 0.582 | 0.813 | 1.38E-105 |
| PKM | 2.40E-103 | -0.684406003 | 0.907 | 0.964 | 7.29E-99 |
| TPM4 | 7.23E-95 | -0.683412251 | 0.849 | 0.937 | 2.20E-90 |
| IFI6 | 3.12E-29 | -0.682200752 | 0.787 | 0.878 | 9.47E-25 |
| ATF3 | 3.54E-51 | -0.681368232 | 0.571 | 0.781 | 1.08E-46 |
| PEBP1 | 2.11E-109 | -0.678703096 | 0.547 | 0.793 | 6.42E-105 |
| MT-CO2 | 1.57E-88 | -0.678189615 | 0.975 | 0.991 | 4.76E-84 |
| SIGLEC1 | 6.68E-85 | -0.675933693 | 0.256 | 0.592 | 2.03E-80 |
| WASF2 | 2.43E-109 | -0.675817937 | 0.474 | 0.781 | 7.37E-105 |
| DBI | 2.10E-91 | -0.674951124 | 0.888 | 0.953 | 6.37E-87 |
| ATP6V0D1 | 5.16E-112 | -0.674738053 | 0.698 | 0.862 | 1.57E-107 |
| CYFIP1 | 2.29E-95 | -0.673451128 | 0.354 | 0.672 | 6.94E-91 |
| SLC15A3 | 6.47E-83 | -0.67326571 | 0.396 | 0.688 | 1.96E-78 |
| IER2 | 3.02E-49 | -0.673158849 | 0.631 | 0.813 | 9.16E-45 |
| M6PR | 4.04E-100 | -0.672718135 | 0.605 | 0.816 | 1.23E-95 |
| 2-Mar | 7.10E-158 | -0.6685307 | 0 | 0.449 | 2.16E-153 |
| PGK1 | 3.21E-89 | -0.665701096 | 0.818 | 0.91 | 9.74E-85 |
| MYH9 | 6.19E-83 | -0.665270509 | 0.506 | 0.762 | 1.88E-78 |
| PLAU | 4.29E-36 | -0.665174831 | 0.377 | 0.576 | 1.30E-31 |
| ADAMDEC1 | 1.03E-41 | -0.66482879 | 0.234 | 0.448 | 3.13E-37 |
| MIR4435-2HG | 3.58E-147 | -0.663993403 | 0 | 0.426 | 1.09E-142 |
| ITGB2 | 7.72E-101 | -0.663521679 | 0.919 | 0.967 | 2.35E-96 |
| VIM | 7.41E-85 | -0.663249266 | 0.995 | 0.998 | 2.25E-80 |
| PPDPF | 8.90E-79 | -0.662410817 | 0.843 | 0.918 | 2.71E-74 |
| LAPTM5 | 6.65E-143 | -0.661392642 | 0.983 | 0.995 | 2.02E-138 |
| MT-CO1 | 1.29E-75 | -0.661331513 | 0.976 | 0.995 | 3.92E-71 |
| PTMS | 2.47E-86 | -0.660958533 | 0.665 | 0.852 | 7.51E-82 |
| CPM | 7.30E-70 | -0.656014011 | 0.416 | 0.694 | 2.22E-65 |
| ARHGAP18 | 9.70E-94 | -0.655396293 | 0.686 | 0.878 | 2.95E-89 |
| RPN2 | 9.09E-101 | -0.655240244 | 0.599 | 0.817 | 2.76E-96 |
| AC100810.1 | 7.71E-166 | -0.654340375 | 0 | 0.465 | 2.34E-161 |
| CFL1 | 2.41E-169 | -0.653792887 | 0.989 | 0.996 | 7.33E-165 |
| AL133415.1 | 5.81E-92 | -0.653608883 | 0 | 0.295 | 1.77E-87 |
| SNX6 | 2.63E-97 | -0.650053891 | 0.583 | 0.833 | 7.99E-93 |
| MSN | 8.99E-88 | -0.650038207 | 0.667 | 0.853 | 2.73E-83 |
| MFSD1 | 4.31E-107 | -0.648687699 | 0.734 | 0.892 | 1.31E-102 |
| COX5A | 8.22E-107 | -0.646985944 | 0.838 | 0.912 | 2.50E-102 |
| HLA-DQA2 | 7.01E-19 | -0.646083616 | 0.609 | 0.655 | 2.13E-14 |
| HEXA | 1.32E-80 | -0.645749779 | 0.602 | 0.808 | 4.00E-76 |
| UQCR11 | 1.03E-105 | -0.645419172 | 0.847 | 0.951 | 3.14E-101 |
| AKR1B1 | 5.64E-78 | -0.64452686 | 0.476 | 0.727 | 1.71E-73 |
| ATP6V0B | 1.72E-116 | -0.642552966 | 0.908 | 0.961 | 5.23E-112 |
| FOSB | 6.23E-33 | -0.641898611 | 0.63 | 0.787 | 1.89E-28 |
| ENO1 | 4.70E-84 | -0.641892865 | 0.888 | 0.953 | 1.43E-79 |
| ALCAM | 6.47E-75 | -0.640429996 | 0.376 | 0.662 | 1.96E-70 |
| MERTK | 4.89E-79 | -0.639659698 | 0.278 | 0.589 | 1.49E-74 |
| GPI | 5.27E-86 | -0.638240515 | 0.555 | 0.778 | 1.60E-81 |
| CD84 | 5.81E-86 | -0.638111058 | 0.399 | 0.705 | 1.77E-81 |
| CAPZB | 2.84E-125 | -0.635902896 | 0.903 | 0.961 | 8.62E-121 |
| LRMDA | 7.55E-165 | -0.635853987 | 0 | 0.463 | 2.29E-160 |
| CHIT1 | 3.87E-31 | -0.634504169 | 0.012 | 0.134 | 1.18E-26 |
| CCDC88A | 2.92E-89 | -0.634149755 | 0.479 | 0.747 | 8.88E-85 |
| DDOST | 2.54E-85 | -0.633907601 | 0.532 | 0.739 | 7.72E-81 |
| IGSF6 | 4.08E-44 | -0.631812133 | 0.811 | 0.857 | 1.24E-39 |
| CALM3 | 4.60E-83 | -0.631722806 | 0.735 | 0.878 | 1.40E-78 |
| ACTG1 | 8.07E-90 | -0.631136797 | 0.979 | 0.991 | 2.45E-85 |
| LY6E | 5.79E-46 | -0.630556246 | 0.761 | 0.891 | 1.76E-41 |
| LRP1 | 4.99E-83 | -0.628546291 | 0.464 | 0.738 | 1.52E-78 |
| ATP6V1F | 1.71E-116 | -0.627253631 | 0.942 | 0.974 | 5.21E-112 |
| CANX | 4.90E-90 | -0.626576963 | 0.748 | 0.886 | 1.49E-85 |
| LAMP1 | 8.67E-87 | -0.626386245 | 0.684 | 0.86 | 2.63E-82 |
| WASHC1 | 1.03E-152 | -0.625737774 | 0 | 0.438 | 3.12E-148 |
| TCEAL9 | 2.32E-131 | -0.625056319 | 0 | 0.391 | 7.06E-127 |
| CCL18 | 1.12E-28 | -0.622195127 | 0.222 | 0.416 | 3.40E-24 |
| ACTR3 | 2.17E-94 | -0.620314432 | 0.713 | 0.866 | 6.59E-90 |
| IL6 | 2.40E-18 | -0.620150346 | 0.114 | 0.228 | 7.30E-14 |
| DUSP2 | 7.62E-33 | -0.619402654 | 0.544 | 0.719 | 2.32E-28 |
| RNASET2 | 1.41E-73 | -0.619348642 | 0.855 | 0.927 | 4.29E-69 |
| GNB4 | 4.41E-81 | -0.618331939 | 0.426 | 0.709 | 1.34E-76 |
| LMNA | 3.69E-53 | -0.618230347 | 0.517 | 0.745 | 1.12E-48 |
| AD000671.2 | 4.48E-142 | -0.617515243 | 0 | 0.415 | 1.36E-137 |
| ANXA5 | 1.69E-132 | -0.616473907 | 0.948 | 0.985 | 5.14E-128 |
| LAIR1 | 8.74E-87 | -0.615791508 | 0.664 | 0.855 | 2.66E-82 |
| QKI | 2.63E-72 | -0.614104673 | 0.394 | 0.675 | 7.99E-68 |
| RGCC | 4.53E-24 | -0.613312266 | 0.401 | 0.554 | 1.38E-19 |
| PAXX | 1.14E-160 | -0.611601049 | 0 | 0.455 | 3.46E-156 |
| TMSB4X | 5.72E-129 | -0.610972435 | 1 | 0.999 | 1.74E-124 |
| CALM1 | 3.21E-88 | -0.610182218 | 0.954 | 0.986 | 9.76E-84 |
| CLTA | 6.03E-109 | -0.609600715 | 0.814 | 0.914 | 1.83E-104 |
| TGOLN2 | 1.75E-90 | -0.608146958 | 0.505 | 0.775 | 5.33E-86 |
| LIMS1 | 1.72E-81 | -0.607594736 | 0.752 | 0.9 | 5.23E-77 |
| COMT | 1.99E-95 | -0.605037162 | 0.738 | 0.88 | 6.04E-91 |
| ITGB1 | 1.22E-83 | -0.604598897 | 0.559 | 0.792 | 3.69E-79 |
| NIPSNAP2 | 4.16E-164 | -0.603592261 | 0 | 0.462 | 1.26E-159 |
| FUCA1 | 8.06E-58 | -0.603241945 | 0.253 | 0.526 | 2.45E-53 |
| VDAC1 | 5.82E-84 | -0.600606388 | 0.646 | 0.823 | 1.77E-79 |
| MS4A4A | 1.85E-49 | -0.600416963 | 0.671 | 0.812 | 5.62E-45 |
| OTULINL | 2.02E-138 | -0.599837417 | 0 | 0.407 | 6.13E-134 |
| CD68 | 2.94E-110 | -0.598433362 | 0.985 | 0.993 | 8.93E-106 |
| CLTC | 5.79E-85 | -0.59695327 | 0.419 | 0.717 | 1.76E-80 |
| MINOS1 | 1.33E-89 | -0.596872999 | 0.701 | 0.874 | 4.04E-85 |
| PGAM1 | 3.78E-60 | -0.595888677 | 0.687 | 0.81 | 1.15E-55 |
| GAPDH | 6.14E-77 | -0.595662593 | 0.995 | 0.999 | 1.87E-72 |
| RASSF4 | 1.75E-62 | -0.594622062 | 0.609 | 0.8 | 5.30E-58 |
| AKR1A1 | 1.36E-88 | -0.594367811 | 0.748 | 0.871 | 4.12E-84 |
| TSPO | 1.91E-65 | -0.594189174 | 0.878 | 0.925 | 5.81E-61 |
| AC015912.3 | 5.50E-84 | -0.592446737 | 0 | 0.274 | 1.67E-79 |
| TMEM219 | 2.61E-100 | -0.592201633 | 0.698 | 0.859 | 7.93E-96 |
| WASHC3 | 2.00E-156 | -0.592073324 | 0 | 0.446 | 6.06E-152 |
| MT-CYB | 9.42E-41 | -0.591675628 | 0.951 | 0.976 | 2.86E-36 |
| HLA-E | 5.12E-101 | -0.590855448 | 0.966 | 0.982 | 1.55E-96 |
| GNPDA1 | 1.33E-76 | -0.589788742 | 0.362 | 0.644 | 4.04E-72 |
| NORAD | 3.55E-137 | -0.589100606 | 0 | 0.405 | 1.08E-132 |
| NDUFB2 | 1.79E-94 | -0.588678516 | 0.84 | 0.936 | 5.43E-90 |
| AP1B1 | 3.86E-68 | -0.588652452 | 0.506 | 0.726 | 1.17E-63 |
| GRB2 | 1.45E-93 | -0.588368152 | 0.844 | 0.93 | 4.42E-89 |
| TMEM123 | 3.63E-69 | -0.587357284 | 0.469 | 0.72 | 1.10E-64 |
| TPM3 | 1.92E-105 | -0.587210933 | 0.93 | 0.972 | 5.83E-101 |
| MFSD12 | 1.38E-70 | -0.586034061 | 0.339 | 0.62 | 4.19E-66 |
| BRI3 | 2.62E-45 | -0.585825985 | 0.803 | 0.877 | 7.97E-41 |
| HEXB | 8.46E-81 | -0.585811235 | 0.706 | 0.844 | 2.57E-76 |
| SAMHD1 | 3.98E-78 | -0.584754081 | 0.758 | 0.883 | 1.21E-73 |
| LDHB | 4.07E-73 | -0.584364284 | 0.486 | 0.714 | 1.24E-68 |
| ISG15 | 5.63E-17 | -0.584011285 | 0.658 | 0.793 | 1.71E-12 |
| DPP7 | 3.68E-92 | -0.583405982 | 0.645 | 0.832 | 1.12E-87 |
| LGALS1 | 2.17E-81 | -0.583128458 | 0.971 | 0.989 | 6.58E-77 |
| SPRED1 | 2.82E-61 | -0.582199673 | 0.279 | 0.551 | 8.56E-57 |
| CD59 | 3.19E-53 | -0.580434801 | 0.449 | 0.672 | 9.71E-49 |
| SLC7A8 | 3.82E-94 | -0.580052055 | 0.132 | 0.485 | 1.16E-89 |
| PRDM1 | 1.40E-34 | -0.579936247 | 0.272 | 0.47 | 4.26E-30 |
| RNF213 | 1.12E-66 | -0.579176704 | 0.606 | 0.83 | 3.40E-62 |
| EML4 | 5.10E-72 | -0.578553993 | 0.554 | 0.78 | 1.55E-67 |
| MRC1 | 9.59E-21 | -0.577919942 | 0.594 | 0.695 | 2.91E-16 |
| IDH1 | 9.34E-85 | -0.577764025 | 0.324 | 0.648 | 2.84E-80 |
| SLC25A5 | 1.85E-75 | -0.577048064 | 0.853 | 0.921 | 5.62E-71 |
| UQCRFS1 | 4.28E-83 | -0.577003973 | 0.517 | 0.738 | 1.30E-78 |
| CTSS | 2.86E-85 | -0.576963596 | 0.938 | 0.974 | 8.69E-81 |
| MTDH | 4.45E-93 | -0.574270766 | 0.759 | 0.896 | 1.35E-88 |
| GUK1 | 2.79E-97 | -0.573423368 | 0.817 | 0.922 | 8.48E-93 |
| LHFPL2 | 5.45E-75 | -0.572851117 | 0.361 | 0.666 | 1.66E-70 |
| HLA-DQB1 | 2.16E-30 | -0.572789118 | 0.936 | 0.955 | 6.56E-26 |
| CCL2 | 0.000621587 | -0.572430432 | 0.175 | 0.222 | 1 |
| TNFAIP2 | 2.82E-37 | -0.570754799 | 0.439 | 0.654 | 8.58E-33 |
| PRDX3 | 4.98E-81 | -0.569883203 | 0.563 | 0.768 | 1.51E-76 |
| STAT1 | 1.57E-42 | -0.569286777 | 0.643 | 0.795 | 4.79E-38 |
| FLNA | 2.53E-44 | -0.56920853 | 0.567 | 0.737 | 7.68E-40 |
| ABHD12 | 9.41E-71 | -0.567818508 | 0.504 | 0.738 | 2.86E-66 |
| ARPC2 | 2.41E-121 | -0.566984956 | 0.964 | 0.98 | 7.32E-117 |
| MCRIP2 | 1.03E-120 | -0.56638591 | 0 | 0.367 | 3.13E-116 |
| HLA-A | 7.14E-64 | -0.566295345 | 0.986 | 0.993 | 2.17E-59 |
| PLEC | 1.51E-58 | -0.565677883 | 0.367 | 0.639 | 4.59E-54 |
| TNS3 | 2.45E-74 | -0.563567243 | 0.272 | 0.585 | 7.44E-70 |
| ERP29 | 1.76E-78 | -0.563453464 | 0.613 | 0.792 | 5.33E-74 |
| ACTR2 | 2.86E-76 | -0.561989598 | 0.707 | 0.855 | 8.68E-72 |
| APH1A | 1.10E-88 | -0.561482482 | 0.494 | 0.757 | 3.35E-84 |
| DNPH1 | 5.47E-75 | -0.56073689 | 0.509 | 0.751 | 1.66E-70 |
| ZNF331 | 1.34E-37 | -0.555108923 | 0.476 | 0.677 | 4.08E-33 |
| IQGAP2 | 3.88E-65 | -0.553385373 | 0.361 | 0.633 | 1.18E-60 |
| CAP1 | 8.50E-85 | -0.553115251 | 0.805 | 0.915 | 2.58E-80 |
| ADAR | 2.31E-63 | -0.553011164 | 0.4 | 0.66 | 7.01E-59 |
| TYMP | 1.88E-71 | -0.552036566 | 0.943 | 0.984 | 5.70E-67 |
| COX4I1 | 1.76E-123 | -0.551940281 | 0.962 | 0.981 | 5.35E-119 |
| LILRB4 | 1.00E-43 | -0.550507173 | 0.754 | 0.836 | 3.04E-39 |
| CD14 | 2.22E-29 | -0.549538181 | 0.887 | 0.939 | 6.76E-25 |
| HSP90B1 | 7.52E-64 | -0.547586678 | 0.858 | 0.924 | 2.29E-59 |
| AP2S1 | 2.38E-87 | -0.546931677 | 0.898 | 0.955 | 7.24E-83 |
| MDH1 | 1.18E-68 | -0.546817306 | 0.505 | 0.716 | 3.58E-64 |
| ME2 | 6.01E-74 | -0.546528709 | 0.281 | 0.592 | 1.83E-69 |
| EVL | 1.10E-66 | -0.546375382 | 0.287 | 0.568 | 3.35E-62 |
| AP2M1 | 8.50E-82 | -0.545447588 | 0.657 | 0.836 | 2.58E-77 |
| BMP2K | 7.81E-70 | -0.544983155 | 0.244 | 0.543 | 2.37E-65 |
| NPM1 | 1.29E-74 | -0.544978901 | 0.849 | 0.923 | 3.92E-70 |
| PFKL | 5.20E-83 | -0.544497112 | 0.434 | 0.713 | 1.58E-78 |
| TLN1 | 8.05E-62 | -0.544433612 | 0.593 | 0.782 | 2.45E-57 |
| NDUFB9 | 1.21E-62 | -0.54425211 | 0.648 | 0.797 | 3.67E-58 |
| WASHC2C | 1.06E-134 | -0.542839044 | 0 | 0.399 | 3.22E-130 |
| ATP6V0E1 | 4.44E-92 | -0.542087627 | 0.897 | 0.944 | 1.35E-87 |
| MMP14 | 1.52E-53 | -0.541854119 | 0.571 | 0.791 | 4.61E-49 |
| SELENOM | 4.42E-114 | -0.541236869 | 0 | 0.351 | 1.34E-109 |
| ARPC5 | 7.23E-95 | -0.541035369 | 0.89 | 0.94 | 2.20E-90 |
| IQGAP1 | 2.05E-64 | -0.540003987 | 0.601 | 0.801 | 6.23E-60 |
| LPL | 7.45E-32 | -0.538677287 | 0.102 | 0.274 | 2.26E-27 |
| MAF | 3.86E-59 | -0.538257021 | 0.254 | 0.525 | 1.17E-54 |
| ABCA1 | 5.83E-50 | -0.537532575 | 0.569 | 0.757 | 1.77E-45 |
| S100A11 | 1.26E-104 | -0.537531638 | 0.997 | 0.999 | 3.81E-100 |
| GCLC | 1.56E-67 | -0.537289587 | 0.111 | 0.391 | 4.74E-63 |
| CD83 | 5.85E-71 | -0.537245256 | 0.555 | 0.851 | 1.78E-66 |
| SLC31A1 | 1.39E-70 | -0.535620767 | 0.369 | 0.646 | 4.23E-66 |
| WDR1 | 5.68E-69 | -0.533013553 | 0.611 | 0.784 | 1.72E-64 |
| NR1H3 | 6.95E-42 | -0.532156285 | 0.3 | 0.527 | 2.11E-37 |
| PCBD1 | 5.41E-75 | -0.531577554 | 0.48 | 0.727 | 1.64E-70 |
| NR4A2 | 6.12E-35 | -0.530703799 | 0.555 | 0.767 | 1.86E-30 |
| CHCHD10 | 2.05E-61 | -0.530659126 | 0.781 | 0.87 | 6.24E-57 |
| NDUFA4 | 3.12E-83 | -0.529967618 | 0.879 | 0.932 | 9.48E-79 |
| NFKBIA | 1.74E-34 | -0.527356078 | 0.829 | 0.948 | 5.29E-30 |
| DMAC1 | 6.43E-134 | -0.527276451 | 0 | 0.397 | 1.95E-129 |
| NCOA4 | 2.26E-59 | -0.527051676 | 0.484 | 0.711 | 6.86E-55 |
| CALR | 1.25E-43 | -0.526658104 | 0.904 | 0.944 | 3.81E-39 |
| LGALS3BP | 1.45E-48 | -0.526568467 | 0.46 | 0.68 | 4.41E-44 |
| FABP3 | 1.51E-66 | -0.525811517 | 0.077 | 0.347 | 4.59E-62 |
| LAMTOR1 | 5.05E-79 | -0.525298699 | 0.705 | 0.859 | 1.54E-74 |
| NCKAP1L | 8.53E-73 | -0.524314402 | 0.405 | 0.685 | 2.59E-68 |
| JUNB | 3.31E-40 | -0.523317532 | 0.805 | 0.942 | 1.01E-35 |
| ARHGAP45 | 7.40E-127 | -0.522501555 | 0 | 0.381 | 2.25E-122 |
| IFIT2 | 4.74E-16 | -0.522279655 | 0.199 | 0.331 | 1.44E-11 |
| ZNF706 | 7.91E-72 | -0.522066655 | 0.728 | 0.864 | 2.40E-67 |
| TWF2 | 2.90E-67 | -0.52159758 | 0.582 | 0.776 | 8.81E-63 |
| RNH1 | 6.66E-72 | -0.521020695 | 0.773 | 0.865 | 2.02E-67 |
| C2 | 6.84E-53 | -0.520779493 | 0.244 | 0.5 | 2.08E-48 |
| RAP1B | 5.34E-68 | -0.519300011 | 0.611 | 0.795 | 1.62E-63 |
| CORO1B | 7.04E-73 | -0.518935262 | 0.61 | 0.79 | 2.14E-68 |
| OLFML3 | 2.69E-27 | -0.518631892 | 0.197 | 0.366 | 8.16E-23 |
| HM13 | 7.14E-61 | -0.518179011 | 0.698 | 0.819 | 2.17E-56 |
| CYP27A1 | 4.04E-34 | -0.517626149 | 0.107 | 0.289 | 1.23E-29 |
| NAGK | 9.93E-63 | -0.517242283 | 0.52 | 0.753 | 3.02E-58 |
| NDUFC1 | 1.42E-71 | -0.517016046 | 0.458 | 0.733 | 4.30E-67 |
| ITM2B | 5.62E-46 | -0.517014758 | 0.976 | 0.985 | 1.71E-41 |
| CALM2 | 2.34E-73 | -0.516577766 | 0.842 | 0.911 | 7.12E-69 |
| SERF2 | 2.18E-99 | -0.515928993 | 0.995 | 0.999 | 6.64E-95 |
| OST4 | 2.00E-74 | -0.515811463 | 0.84 | 0.933 | 6.09E-70 |
| IGF2R | 1.92E-66 | -0.515304095 | 0.295 | 0.58 | 5.85E-62 |
| BUD23 | 4.88E-135 | -0.513831091 | 0 | 0.4 | 1.48E-130 |
| NOP10 | 7.51E-59 | -0.512929817 | 0.818 | 0.902 | 2.28E-54 |
| SERBP1 | 5.66E-74 | -0.51141983 | 0.577 | 0.779 | 1.72E-69 |
| NME2 | 3.27E-66 | -0.511286065 | 0.926 | 0.958 | 9.94E-62 |
| COX8A | 2.74E-82 | -0.510854957 | 0.91 | 0.96 | 8.31E-78 |
| PRRC2C | 9.33E-64 | -0.510725797 | 0.554 | 0.772 | 2.84E-59 |
| HK3 | 5.05E-60 | -0.510610571 | 0.216 | 0.486 | 1.54E-55 |
| SPTAN1 | 1.23E-60 | -0.510588108 | 0.233 | 0.512 | 3.74E-56 |
| CD164 | 1.09E-67 | -0.510449119 | 0.681 | 0.833 | 3.32E-63 |
| NEAT1 | 5.88E-30 | -0.509796113 | 0.9 | 0.962 | 1.79E-25 |
| VEGFB | 3.92E-65 | -0.509450102 | 0.149 | 0.429 | 1.19E-60 |
| GLMP | 1.89E-61 | -0.507317754 | 0.439 | 0.672 | 5.74E-57 |
| MPP1 | 2.48E-57 | -0.50464866 | 0.59 | 0.775 | 7.53E-53 |
| PDIA6 | 2.41E-47 | -0.50382271 | 0.609 | 0.766 | 7.31E-43 |
| MYL6 | 3.28E-91 | -0.503520002 | 0.993 | 0.998 | 9.96E-87 |
| NFKBIZ | 2.80E-23 | -0.50348394 | 0.597 | 0.724 | 8.50E-19 |
| RPL3 | 3.85E-73 | -0.502896436 | 0.94 | 0.971 | 1.17E-68 |
| SNX9 | 3.11E-65 | -0.502509103 | 0.342 | 0.641 | 9.46E-61 |
| GLUL | 1.41E-45 | -0.502443909 | 0.958 | 0.986 | 4.28E-41 |
| TUBA1A | 6.68E-45 | -0.502053974 | 0.52 | 0.717 | 2.03E-40 |
| CXCL16 | 2.49E-63 | -0.502011061 | 0.775 | 0.907 | 7.56E-59 |
| ATP6V0C | 5.36E-67 | -0.501837768 | 0.954 | 0.965 | 1.63E-62 |
| FAM96A | 1.38E-69 | -0.501639732 | 0.656 | 0.82 | 4.21E-65 |
| NCF2 | 3.22E-41 | -0.500554075 | 0.614 | 0.779 | 9.78E-37 |
| NSD3 | 7.40E-127 | -0.50023778 | 0 | 0.381 | 2.25E-122 |
| NAP1L1 | 7.11E-55 | -0.499256016 | 0.592 | 0.764 | 2.16E-50 |
| RPS4Y1 | 2.86E-54 | -0.498432736 | 0.495 | 0.738 | 8.68E-50 |
| SIRPA | 2.01E-56 | -0.497967512 | 0.575 | 0.765 | 6.10E-52 |
| DNASE1L3 | 8.21E-08 | -0.497697404 | 0.066 | 0.123 | 0.002493531 |
| SLC25A3 | 2.35E-77 | -0.497563692 | 0.782 | 0.893 | 7.14E-73 |
| NEK6 | 4.28E-59 | -0.497034241 | 0.35 | 0.609 | 1.30E-54 |
| MIF | 2.97E-34 | -0.496304255 | 0.943 | 0.971 | 9.01E-30 |
| SDCBP | 6.71E-73 | -0.496068081 | 0.834 | 0.929 | 2.04E-68 |
| NDUFAB1 | 2.54E-58 | -0.495512679 | 0.584 | 0.762 | 7.71E-54 |
| KDELR1 | 7.81E-68 | -0.495259548 | 0.649 | 0.8 | 2.37E-63 |
| RRBP1 | 4.73E-57 | -0.495242636 | 0.454 | 0.689 | 1.44E-52 |
| NFE2L2 | 1.74E-47 | -0.493959506 | 0.586 | 0.782 | 5.28E-43 |
| DUSP23 | 3.40E-65 | -0.493503883 | 0.446 | 0.701 | 1.03E-60 |
| ATP5PO | 2.48E-115 | -0.493499129 | 0 | 0.354 | 7.53E-111 |
| IFIT3 | 4.59E-21 | -0.49349792 | 0.282 | 0.445 | 1.39E-16 |
| ATP6AP1 | 5.55E-63 | -0.493290729 | 0.738 | 0.854 | 1.69E-58 |
| CD151 | 3.22E-57 | -0.492923839 | 0.441 | 0.673 | 9.80E-53 |
| OAS1 | 2.01E-36 | -0.491119421 | 0.332 | 0.544 | 6.10E-32 |
| LAMP2 | 7.70E-56 | -0.490495569 | 0.557 | 0.74 | 2.34E-51 |
| PSMA7 | 8.01E-68 | -0.490277707 | 0.883 | 0.939 | 2.43E-63 |
| PTPA | 6.29E-121 | -0.490147844 | 0 | 0.367 | 1.91E-116 |
| TCIRG1 | 2.56E-58 | -0.490032646 | 0.66 | 0.815 | 7.77E-54 |
| EPHX1 | 4.47E-55 | -0.489438569 | 0.156 | 0.413 | 1.36E-50 |
| TMEM37 | 3.15E-86 | -0.489105355 | 0.057 | 0.369 | 9.58E-82 |
| EGR2 | 6.54E-38 | -0.488794284 | 0.093 | 0.28 | 1.99E-33 |
| DNASE2 | 1.80E-43 | -0.488742558 | 0.458 | 0.648 | 5.47E-39 |
| SDC2 | 7.45E-42 | -0.488495087 | 0.27 | 0.495 | 2.26E-37 |
| NFIC | 1.31E-67 | -0.488163582 | 0.275 | 0.571 | 3.97E-63 |
| TENT2 | 1.22E-117 | -0.487122043 | 0 | 0.36 | 3.70E-113 |
| PAK2 | 1.09E-64 | -0.486562009 | 0.559 | 0.781 | 3.32E-60 |
| TENT5A | 2.64E-100 | -0.486421745 | 0 | 0.317 | 8.02E-96 |
| EEF2 | 6.48E-66 | -0.485964262 | 0.83 | 0.907 | 1.97E-61 |
| MYDGF | 4.21E-52 | -0.485865313 | 0.696 | 0.801 | 1.28E-47 |
| MMP9 | 2.49E-06 | -0.485717553 | 0.488 | 0.559 | 0.075737254 |
| TMEM14C | 1.74E-66 | -0.485669986 | 0.567 | 0.762 | 5.29E-62 |
| BLVRA | 1.21E-56 | -0.485433851 | 0.51 | 0.732 | 3.68E-52 |
| FAM20C | 1.49E-73 | -0.485347103 | 0.122 | 0.427 | 4.54E-69 |
| CD4 | 1.02E-52 | -0.484938855 | 0.629 | 0.802 | 3.10E-48 |
| LAP3 | 2.26E-45 | -0.484496123 | 0.723 | 0.829 | 6.86E-41 |
| HLA-DMA | 6.51E-44 | -0.484366621 | 0.939 | 0.948 | 1.98E-39 |
| TNFRSF14 | 5.03E-56 | -0.484081268 | 0.509 | 0.735 | 1.53E-51 |
| HIKESHI | 1.28E-127 | -0.481232361 | 0 | 0.383 | 3.87E-123 |
| MATK | 1.34E-41 | -0.481040202 | 0.129 | 0.339 | 4.06E-37 |
| ABRACL | 3.01E-57 | -0.478972603 | 0.548 | 0.742 | 9.14E-53 |
| APOC2 | 5.06E-28 | -0.478784228 | 0.05 | 0.182 | 1.54E-23 |
| ARL6IP4 | 4.52E-68 | -0.478074066 | 0.715 | 0.855 | 1.37E-63 |
| SCAMP2 | 2.86E-58 | -0.477972951 | 0.527 | 0.73 | 8.70E-54 |
| BLOC1S2 | 3.81E-70 | -0.47664823 | 0.295 | 0.588 | 1.16E-65 |
| CALU | 9.40E-69 | -0.476535548 | 0.271 | 0.568 | 2.86E-64 |
| CD37 | 8.66E-43 | -0.475836648 | 0.731 | 0.833 | 2.63E-38 |
| TPP1 | 9.64E-55 | -0.475776395 | 0.677 | 0.823 | 2.93E-50 |
| RTN4 | 5.20E-60 | -0.474315334 | 0.711 | 0.849 | 1.58E-55 |
| MT1G | 1.74E-09 | -0.473508623 | 0.196 | 0.29 | 5.27E-05 |
| ST14 | 7.74E-52 | -0.473099707 | 0.327 | 0.583 | 2.35E-47 |
| TAF10 | 5.50E-36 | -0.472099078 | 0.349 | 0.54 | 1.67E-31 |
| RGS1 | 3.23E-38 | -0.472061466 | 0.671 | 0.853 | 9.83E-34 |
| ROMO1 | 1.37E-57 | -0.471575849 | 0.664 | 0.85 | 4.17E-53 |
| TMBIM6 | 2.27E-68 | -0.471540711 | 0.913 | 0.944 | 6.89E-64 |
| TMEM176A | 1.86E-34 | -0.470814899 | 0.77 | 0.874 | 5.66E-30 |
| NDUFV2 | 2.15E-57 | -0.470229699 | 0.697 | 0.826 | 6.52E-53 |
| TYROBP | 5.66E-100 | -0.46974425 | 0.999 | 1 | 1.72E-95 |
| CD276 | 2.05E-64 | -0.469723555 | 0.21 | 0.497 | 6.22E-60 |
| METRNL | 2.16E-28 | -0.469114015 | 0.401 | 0.581 | 6.57E-24 |
| NDUFB7 | 1.58E-60 | -0.468740189 | 0.606 | 0.804 | 4.81E-56 |
| TFRC | 1.19E-30 | -0.467567751 | 0.422 | 0.612 | 3.62E-26 |
| MPEG1 | 6.78E-30 | -0.466875288 | 0.386 | 0.551 | 2.06E-25 |
| VAMP8 | 1.63E-82 | -0.466415078 | 0.936 | 0.97 | 4.96E-78 |
| SLC31A2 | 5.62E-50 | -0.465994337 | 0.567 | 0.736 | 1.71E-45 |
| ANXA4 | 8.16E-60 | -0.465869265 | 0.391 | 0.65 | 2.48E-55 |
| COX6C | 1.72E-71 | -0.465848431 | 0.868 | 0.944 | 5.22E-67 |
| CAPZA2 | 1.51E-63 | -0.465730977 | 0.616 | 0.793 | 4.59E-59 |
| SYK | 7.90E-57 | -0.465375688 | 0.433 | 0.676 | 2.40E-52 |
| HNRNPU | 1.33E-49 | -0.464977636 | 0.642 | 0.829 | 4.03E-45 |
| FUCA2 | 2.23E-59 | -0.464970827 | 0.411 | 0.648 | 6.78E-55 |
| SPG21 | 2.71E-61 | -0.464820213 | 0.541 | 0.747 | 8.23E-57 |
| RAP2B | 4.78E-60 | -0.464580814 | 0.3 | 0.577 | 1.45E-55 |
| WASHC2A | 2.44E-109 | -0.464578416 | 0 | 0.34 | 7.40E-105 |
| OSTF1 | 4.28E-61 | -0.464180596 | 0.65 | 0.806 | 1.30E-56 |
| RPLP1 | 7.54E-92 | -0.464120114 | 0.999 | 0.999 | 2.29E-87 |
| GAA | 1.57E-54 | -0.463156353 | 0.517 | 0.735 | 4.78E-50 |
| SLC49A3 | 3.80E-113 | -0.462408873 | 0 | 0.349 | 1.15E-108 |
| GUSB | 1.37E-55 | -0.462275275 | 0.419 | 0.641 | 4.16E-51 |
| PPIA | 2.79E-99 | -0.462022452 | 0.994 | 0.993 | 8.49E-95 |
| BHLHE40 | 6.17E-31 | -0.460487462 | 0.458 | 0.641 | 1.87E-26 |
| MLF2 | 2.49E-62 | -0.460418346 | 0.472 | 0.709 | 7.56E-58 |
| LAPTM4A | 3.50E-51 | -0.460293463 | 0.705 | 0.822 | 1.06E-46 |
| PPP1CA | 1.88E-64 | -0.459235001 | 0.726 | 0.835 | 5.72E-60 |
| NPL | 8.93E-55 | -0.4589026 | 0.384 | 0.637 | 2.71E-50 |
| ATP6V1A | 2.73E-65 | -0.458894206 | 0.273 | 0.57 | 8.30E-61 |
| MOB1A | 4.16E-58 | -0.458604194 | 0.686 | 0.843 | 1.26E-53 |
| FPR3 | 3.39E-38 | -0.458297288 | 0.566 | 0.742 | 1.03E-33 |
| FGD5-AS1 | 2.48E-115 | -0.457955194 | 0 | 0.354 | 7.53E-111 |
| PEPD | 4.93E-58 | -0.457916144 | 0.424 | 0.665 | 1.50E-53 |
| ARHGDIA | 3.19E-56 | -0.457785262 | 0.685 | 0.832 | 9.69E-52 |
| NCL | 3.75E-49 | -0.457779909 | 0.648 | 0.808 | 1.14E-44 |
| LAMTOR2 | 1.33E-60 | -0.45735101 | 0.677 | 0.822 | 4.06E-56 |
| SELENON | 6.50E-108 | -0.457009641 | 0 | 0.336 | 1.98E-103 |
| SNX17 | 9.63E-62 | -0.456823049 | 0.369 | 0.629 | 2.93E-57 |
| CNDP2 | 4.70E-52 | -0.456130058 | 0.554 | 0.749 | 1.43E-47 |
| PFN1 | 1.01E-80 | -0.455924693 | 0.993 | 0.998 | 3.07E-76 |
| CDC42 | 7.44E-65 | -0.455469303 | 0.797 | 0.898 | 2.26E-60 |
| SRP19 | 9.33E-80 | -0.455460847 | 0.157 | 0.481 | 2.84E-75 |
| TMEM179B | 2.36E-58 | -0.454749505 | 0.569 | 0.761 | 7.17E-54 |
| AXL | 8.87E-49 | -0.454523035 | 0.233 | 0.475 | 2.69E-44 |
| ANAPC11 | 4.30E-61 | -0.453805303 | 0.753 | 0.869 | 1.31E-56 |
| RAB9A | 1.36E-60 | -0.453556335 | 0.257 | 0.536 | 4.13E-56 |
| IFI27 | 6.54E-09 | -0.452127712 | 0.3 | 0.397 | 0.000198621 |
| NAA20 | 2.70E-66 | -0.451526687 | 0.309 | 0.587 | 8.20E-62 |
| RER1 | 4.55E-60 | -0.451492827 | 0.572 | 0.758 | 1.38E-55 |
| NPTN | 1.24E-62 | -0.451264611 | 0.302 | 0.577 | 3.76E-58 |
| SLC43A3 | 7.22E-49 | -0.451074587 | 0.371 | 0.605 | 2.19E-44 |
| ARF1 | 6.18E-68 | -0.45066071 | 0.81 | 0.889 | 1.88E-63 |
| PPT1 | 4.18E-47 | -0.450589897 | 0.767 | 0.861 | 1.27E-42 |
| RAB5C | 2.05E-53 | -0.450390885 | 0.742 | 0.849 | 6.24E-49 |
| NDUFA6 | 4.42E-51 | -0.450085183 | 0.652 | 0.796 | 1.34E-46 |
| B3GNT5 | 2.28E-16 | 0.450228631 | 0.459 | 0.369 | 6.92E-12 |
| FO538757.2 | 1.35E-297 | 0.451001941 | 0.318 | 0 | 4.12E-293 |
| CTD.2336O2.1 | 2.17E-281 | 0.457969994 | 0.302 | 0 | 6.59E-277 |
| X2.Mar | 4.40E-284 | 0.458937526 | 0.304 | 0 | 1.34E-279 |
| KIAA1033 | 4.41E-293 | 0.459440251 | 0.313 | 0 | 1.34E-288 |
| C6orf1 | 7.82E-305 | 0.460591263 | 0.325 | 0 | 2.38E-300 |
| HLA-C | 1.47E-55 | 0.466187956 | 0.991 | 0.989 | 4.47E-51 |
| CTA.29F11.1 | 2.86E-194 | 0.466956134 | 0.212 | 0 | 8.69E-190 |
| C9orf142 | 0 | 0.472832499 | 0.331 | 0 | 0 |
| RGS2 | 6.54E-09 | 0.479090861 | 0.733 | 0.713 | 0.000198754 |
| APOA1BP | 0 | 0.48446749 | 0.336 | 0 | 0 |
| 6-Sep | 0 | 0.487822955 | 0.348 | 0 | 0 |
| TWISTNB | 6.01E-05 | 0.489337472 | 0.335 | 0.3 | 1 |
| RP11.670E13.6 | 7.33E-197 | 0.491352167 | 0.214 | 0 | 2.23E-192 |
| CD300E | 5.55E-74 | 0.492049002 | 0.259 | 0.068 | 1.69E-69 |
| SOX4 | 0.042972726 | 0.499288658 | 0.228 | 0.22 | 1 |
| 9-Sep | 0 | 0.499761439 | 0.34 | 0 | 0 |
| ADM | 3.58E-14 | 0.502010726 | 0.401 | 0.31 | 1.09E-09 |
| IGKV4.1 | 1.19E-204 | 0.513400717 | 0.223 | 0 | 3.60E-200 |
| MALAT1 | 3.14E-26 | 0.514403733 | 0.953 | 0.986 | 9.53E-22 |
| ZMYM6NB | 0 | 0.518671769 | 0.351 | 0 | 0 |
| FAM195B | 0 | 0.518859789 | 0.348 | 0 | 0 |
| STRA13 | 0 | 0.525385689 | 0.348 | 0 | 0 |
| UFD1L | 0 | 0.528643419 | 0.362 | 0 | 0 |
| HIST2H2AA4 | 1.20E-144 | 0.531666669 | 0.263 | 0.03 | 3.65E-140 |
| C10orf11 | 0 | 0.532842212 | 0.383 | 0 | 0 |
| IGKV1.5 | 7.52E-238 | 0.532903415 | 0.257 | 0 | 2.28E-233 |
| RP11.796E2.4 | 7.33E-197 | 0.534370099 | 0.214 | 0 | 2.23E-192 |
| LINC01420 | 0 | 0.540258856 | 0.375 | 0 | 0 |
| FAM127A | 0 | 0.544857148 | 0.365 | 0 | 0 |
| HSPD1 | 0.485802921 | 0.552155166 | 0.724 | 0.782 | 1 |
| CCDC109B | 0 | 0.558036168 | 0.358 | 0 | 0 |
| WBP5 | 5.09E-244 | 0.575333242 | 0.263 | 0 | 1.55E-239 |
| C17orf62 | 0 | 0.578390354 | 0.407 | 0 | 0 |
| C16orf13 | 0 | 0.58935315 | 0.4 | 0 | 0 |
| IDO1 | 1.58E-35 | 0.591885643 | 0.196 | 0.073 | 4.80E-31 |
| SERPINH1 | 1.78E-09 | 0.592076429 | 0.322 | 0.27 | 5.41E-05 |
| MIR4435.2HG | 0 | 0.59420167 | 0.354 | 0 | 0 |
| SNAI1 | 5.29E-28 | 0.599012351 | 0.402 | 0.257 | 1.61E-23 |
| LINC00116 | 0 | 0.599358878 | 0.347 | 0 | 0 |
| HSPE1 | 0.246538216 | 0.601713379 | 0.827 | 0.873 | 1 |
| AD000671.6 | 0 | 0.614601343 | 0.401 | 0 | 0 |
| LINC00936 | 7.32E-253 | 0.614869827 | 0.272 | 0 | 2.22E-248 |
| HSP90AA1 | 2.37E-05 | 0.622312831 | 0.967 | 0.989 | 0.721138952 |
| IGKV3.20 | 1.49E-247 | 0.622477098 | 0.267 | 0 | 4.54E-243 |
| HSPA1A | 2.88E-10 | 0.625181454 | 0.872 | 0.928 | 8.74E-06 |
| MESDC2 | 0 | 0.629318095 | 0.406 | 0 | 0 |
| X1.Mar | 0 | 0.629628156 | 0.392 | 0 | 0 |
| CLK1 | 3.34E-14 | 0.630910745 | 0.553 | 0.511 | 1.02E-09 |
| PHLDA2 | 6.69E-14 | 0.641843584 | 0.477 | 0.398 | 2.03E-09 |
| IGLV1.40 | 1.30E-156 | 0.643273851 | 0.172 | 0 | 3.95E-152 |
| RTFDC1 | 0 | 0.645894237 | 0.447 | 0 | 0 |
| SLC2A3 | 7.94E-14 | 0.656590772 | 0.645 | 0.609 | 2.41E-09 |
| BTG1 | 1.06E-19 | 0.688215658 | 0.848 | 0.859 | 3.22E-15 |
| CEBPD | 1.50E-29 | 0.695620092 | 0.807 | 0.731 | 4.55E-25 |
| LINC00152 | 0 | 0.70338797 | 0.388 | 0 | 0 |
| KLF2 | 9.39E-19 | 0.705715469 | 0.404 | 0.302 | 2.85E-14 |
| RASD1 | 3.71E-85 | 0.706036304 | 0.23 | 0.047 | 1.13E-80 |
| C7orf73 | 0 | 0.721483717 | 0.483 | 0 | 0 |
| FCN1 | 8.01E-53 | 0.725626461 | 0.243 | 0.079 | 2.44E-48 |
| ID3 | 1.04E-06 | 0.744947576 | 0.484 | 0.449 | 0.031567382 |
| 7-Sep | 0 | 0.761035525 | 0.502 | 0 | 0 |
| VIMP | 0 | 0.76353902 | 0.477 | 0 | 0 |
| LINC00493 | 0 | 0.773787831 | 0.511 | 0 | 0 |
| CLEC4E | 4.28E-90 | 0.775485017 | 0.518 | 0.238 | 1.30E-85 |
| 2-Sep | 0 | 0.782701505 | 0.501 | 0 | 0 |
| GLTSCR2 | 0 | 0.787496261 | 0.506 | 0 | 0 |
| MMP24.AS1 | 0 | 0.797489377 | 0.5 | 0 | 0 |
| C14orf166 | 0 | 0.805761617 | 0.547 | 0 | 0 |
| SELT | 0 | 0.810224895 | 0.528 | 0 | 0 |
| ATP5G1 | 0 | 0.823582741 | 0.531 | 0 | 0 |
| GADD45G | 2.64E-26 | 0.831003306 | 0.401 | 0.269 | 8.03E-22 |
| HSPA1B | 1.24E-15 | 0.833824376 | 0.788 | 0.834 | 3.78E-11 |
| DNAJB4 | 1.09E-33 | 0.839416632 | 0.354 | 0.214 | 3.30E-29 |
| IGKV3.15 | 0 | 0.849280543 | 0.361 | 0 | 0 |
| HSPH1 | 4.66E-10 | 0.866609247 | 0.697 | 0.739 | 1.42E-05 |
| S100A8 | 2.66E-17 | 0.870630363 | 0.61 | 0.533 | 8.08E-13 |
| ATP5A1 | 0 | 0.876951432 | 0.547 | 0 | 0 |
| RHOB | 3.18E-15 | 0.877430695 | 0.698 | 0.692 | 9.66E-11 |
| MYEOV2 | 0 | 0.881967498 | 0.534 | 0 | 0 |
| DNAJA4 | 1.41E-28 | 0.91351606 | 0.415 | 0.288 | 4.27E-24 |
| SQRDL | 0 | 0.926447117 | 0.572 | 0 | 0 |
| X15.Sep | 0 | 0.953294972 | 0.596 | 0 | 0 |
| DNAJB1 | 3.32E-15 | 0.963993982 | 0.812 | 0.851 | 1.01E-10 |
| C20orf24 | 0 | 0.975134029 | 0.565 | 0 | 0 |
| IER5 | 8.38E-33 | 0.984615396 | 0.639 | 0.556 | 2.55E-28 |
| PVRL2 | 0 | 1.011201786 | 0.6 | 0 | 0 |
| CECR1 | 0 | 1.024107154 | 0.558 | 0 | 0 |
| C19orf60 | 0 | 1.069969927 | 0.64 | 0 | 0 |
| FAM46A | 0 | 1.084673548 | 0.369 | 0 | 0 |
| ATP5F1 | 0 | 1.095403636 | 0.655 | 0 | 0 |
| TCEB1 | 0 | 1.116093296 | 0.634 | 0 | 0 |
| TIMP1 | 1.56E-35 | 1.121357667 | 0.823 | 0.757 | 4.74E-31 |
| HSPB1 | 4.87E-25 | 1.130895553 | 0.887 | 0.904 | 1.48E-20 |
| LINC01272 | 0 | 1.146959816 | 0.542 | 0 | 0 |
| ATPIF1 | 0 | 1.175858726 | 0.649 | 0 | 0 |
| ZFAND2A | 1.13E-33 | 1.205506399 | 0.46 | 0.328 | 3.42E-29 |
| DDIT4 | 6.19E-64 | 1.230396855 | 0.675 | 0.472 | 1.88E-59 |
| C19orf43 | 0 | 1.268979134 | 0.74 | 0 | 0 |
| BAG3 | 9.91E-51 | 1.300004883 | 0.573 | 0.41 | 3.01E-46 |
| ATP5C1 | 0 | 1.317716483 | 0.748 | 0 | 0 |
| ATP5I | 0 | 1.339683464 | 0.629 | 0 | 0 |
| SEPW1 | 0 | 1.414174579 | 0.737 | 0 | 0 |
| C14orf2 | 0 | 1.423203194 | 0.729 | 0 | 0 |
| C10orf54 | 0 | 1.446434824 | 0.685 | 0 | 0 |
| HN1 | 0 | 1.459453544 | 0.705 | 0 | 0 |
| ATP5J | 0 | 1.474808615 | 0.757 | 0 | 0 |
| SHFM1 | 0 | 1.486377558 | 0.767 | 0 | 0 |
| C11orf31 | 0 | 1.506568743 | 0.775 | 0 | 0 |
| ATP5B | 0 | 1.530066628 | 0.787 | 0 | 0 |
| SELK | 0 | 1.58469509 | 0.667 | 0 | 0 |
| ATP5D | 0 | 1.587098897 | 0.798 | 0 | 0 |
| FYB | 0 | 1.675182453 | 0.737 | 0 | 0 |
| ATP5H | 0 | 1.69089976 | 0.833 | 0 | 0 |
| USMG5 | 0 | 1.808706556 | 0.815 | 0 | 0 |
| FAM26F | 0 | 1.810874314 | 0.699 | 0 | 0 |
| APOC4.APOC2 | 1.30E-239 | 1.857911239 | 0.259 | 0 | 3.96E-235 |
| ATP5G3 | 0 | 1.868370477 | 0.837 | 0 | 0 |
| ATP5J2 | 0 | 1.892428125 | 0.823 | 0 | 0 |
| RPS10 | 0 | 1.950536714 | 0.983 | 0.779 | 0 |
| HSPA6 | 6.89E-68 | 2.146768369 | 0.588 | 0.396 | 2.09E-63 |
| TCEB2 | 0 | 2.229062118 | 0.874 | 0 | 0 |
| ATP5L | 0 | 2.400789108 | 0.926 | 0 | 0 |
| ATP5G2 | 0 | 2.532927474 | 0.939 | 0 | 0 |
| CCL3L3 | 5.09E-244 | 2.639504438 | 0.263 | 0 | 1.55E-239 |
| GNB2L1 | 0 | 2.984289326 | 0.965 | 0 | 0 |
| GPX1 | 0 | 3.340844372 | 0.975 | 0 | 0 |
| ATP5E | 0 | 3.747134687 | 0.972 | 0 | 0 |
| AC090498.1 | 0 | 4.463661712 | 0.994 | 0 | 0 |
