# Supplementary material for: Identification of a distinct cluster of LY6E+ macrophages in esophageal squamous cell carcinoma: functional phenotype, spatial interaction, and prognostic significance
Source: Br J Cancer. 2026 Apr 29;135(3):382–93. doi: 10.1038/s41416-026-03456-4 (PMC13372808; doi:10.1038/s41416-026-03456-4)
Supplement: Supplementary file 4 — Supplementary table3 [file 41416_2026_3456_MOESM4_ESM.docx]

| M1-like | "IL23","TNF","CXCL9","CXCL10","CXCL11","CD86","IL1A","IL1B","IL6","CCL5","IRF5","IRF1","CD40","IDO1","KYNU","CCR7", |
| --- | --- |
| M2-like | "IL4R","CCL4","CCL13","CCL20","CCL17","CCL18","CCL22","CCL24","LYVE1","VEGFA","VEGFB","VEGFC","VEGFD","EGF","CTSA","CTSB","CTSC","CTSD","TGFB1","TGFB2","TGFB3","MMP14","MMP19","MMP9","CLEC7A","WNT7B","FASL","TNFSF12","TNFSF8","CD276","VTCN1","MSR1","FN1","IRF4" |
| Phagosome | "PIK3C3","FAB1","C1R","CTSL","CTSS","ATPeV1H","ATP6A","ATP6B","ATP6C","ATP6D","ATP6E","ATP6F","ATP6G","ATP6H","ATP6N","ATP6L","ATP6S14","ATP6Sl","C3","MBL","CD14","RAC1","THBS2S","ABCB2","ABCB3","ACTB_G1","ITGB1","ITGB2","ITGAM","CD36","FCGR3","FCGR2A","ITGA2","ITGA5","ITGAV","ITGB3","FCGR1A","TFRC","FCAR","CD107","MSR1","MRC","CD209","ITGB5","SEC61G","IGH","TUBA","TUBB","RAB5A","RAB5B","RAB7A","RAB7B","RAB5C","CYBA","NCF1","NCF2","NCF4","CANX","CALR","STX7","STX18","SEC22","OLR1","SEC61B","COLEC12","COLEC11","SFTPA","SFTPD","CLEC7A","M6PR","TLR2","TLR4","TLR6","DYNC1H","DYNC2H","DYNC1I","DYNC1LI","MPO","SEC61A","HGS","EEA1","FCGR2B","NOS1","VAMP3","STX12","CORO1A","RILP","MARCO","SCARB1","FCGR2C","THBS1","NOX2" |
| Angiogenesis | "CCND2","CCNE1","CD44","CXCR4","E2F3","EDN1","EZH2","FGF18","FGFR1","FYN","HEY1","ITGAV","JAG1","JAG2","MMP9","NOTCH1","PDGFA","PTK2","SPP1","STC1","TNFAIP6","TYMP","VAV2","VCAN","VEGFA" |
| Antigen presentation | "B2M","CALR","CANX","CD4","CD74","CD8A","CD8B","CIITA","CREB1","CTSB","CTSL","CTSS","HLA-A","HLA-B","HLA-C","HLA-DMA","HLA-DMB","HLA-DOA","HLA-DOB","HLA-DPA1","HLA-DPB1","HLA-DQA1","HLA-DQA2","HLA-DQB1","HLA-DRA","HLA-DRB1","HLA-DRB3","HLA-DRB4","HLA-DRB5","HLA-E","HLA-F","HLA-G","HSP90AA1","HSP90AB1","HSPA1A","HSPA1B","HSPA1L","HSPA2","HSPA4",'HSPA5','HSPA6','HSPA8',"IFI30","IFNA1","IFNA10",'IFNA13',"IFNA14",'IFNA16',"IFNA17","IFNA2","IFNA21","IFNA4",'IFNA5',"IFNA6","IFNA7","IFNA8","KIR2DL1","KIR2DL2","KIR2DL3","KIR2DL4","KIR2DL5A","KIR2DS1","KIR2DS3","KIR2DS4","KIR2DS5","KIR3DL1","KIR3DL2","KIR3DL3","KLRC1","KLRC2","KLRC3","KLRC4","KLRD1","LGMN","LTA","NFYA","NFYB","NFYC","PDIA3","PSME1","PSME2","PSME3","RFX5","RFXANK","RFXAP","TAP1","TAP2","TAPBP" |
| Lysosome | "LIPA","PPT","NAGPA","GALNS","ARSA","ARSB","IDS","GNS","DNASE2","NEU1","GLA","MANBA","GUSB","GBA","GALC","NAGA","NAGLU","FUCA","IDUA","CTSC","TPP1","CTSB","CTSG","CTSL","CTSS","CTSH","CTSK","CTSF","CTS0","LGMN","CTSD","CTSE","LGMN","AGA","SGSH","ATPeV1H","ATP6D","ATP6N","ATP6L","ATP6F","ATP6Sl","CLTA","CLTB","CLTC","MCOLN1","ABCA2","ABCB9","LYPLA3","CD63","CD68","CD107","CD164","LAMP3","IGF2R","GNPTAB","NAPSA","CTSZ","CTSW","CTSP","CTSM","GNPTG","M6PR","HGSNAT","SLC17A5","ENTPD4","MSFD8","GLB1","MAN2B1","GAA","SLC11A1","ASAH1","SMPD1","HEXA_B","ARSG","PSAP","GM2A","LIMP2","NPC1","CTNS","LAPTM","SORT1","BTS","CLN5","AP1G1","AP1B1","AP1M","AP1S1_2","AP1S3","AP3D","AP3B","AP3M","AP3S","AP4E1","AP4B1", "AP4M1", "AP4S1", "GGA","CTSA","NPC2","SUMF1","ACP5","ACP2","LITAF","SLC11A2","DMXL","WDR7","NCOA7" |
| Immune inhibiting | "CD274","PDCD1LG2","LGALS9","SIGLEC10","VSIR","VSIG4","SIRPA" |
| niche signature | "APOE","SPP1","APOC1","CTSB","ACP5","TREM2","GPNMB","FBP1","CTSD","LIPA","PLA2G7","CAPG","CXCL9","IFI6","FABP5","LY6E","CD9","MMP9","MMP12","ISG15","CXCL13","GZMB","KRT86","GNLY","ITGAE","LAG3","GZMA","CD8A","CSF1","PLPP1","KIR2DL4","HAVCR2","ALOX5AP","KLRC1","ACP5","CCL5","LINC01871","RGS2","SOX4","CXCR6","GRP","SEC11C","TFF3","ASCL1","IGFBP5","BEX1","SMAD9","MGST1","TUBA1A","TAGLN3","CDKN2C","NR2F1","ID2","ICAM2","COTL1","ALCAM","SYT1","XBP1","LSR","DDC" |
| LY6E+ macrophage | APOE,"SPP1","APOC1","CTSB","ACP5","TREM2","GPNMB","FBP1","CTSD","LIPA","PLA2G7","CAPG","CXCL9","IFI6","FABP5","LY6E","CD9","MMP9","MMP12","ISG15" |
| Progenitor Exhausted CD8+ T Cell Signature | IL7R GPR183 LMNA NR4A3 TCF7 MGAT4A CD55 AIM1 PER1 FOSL2 EGR1 TSPYL2 YPEL5 CSRNP1 REL SKIL PIK3R1 FOXP1 RGCC PFKFB3 MYADM ZFP36L2 USP36 TC2N FAM177A1 BTG2 TSC22D2 FAM65B STAT4 RGPD5 NEU1 IFRD1 PDE4B NR4A1 |
| Terminally Exhausted CD8+ T Cell Signature | NKG7 RAC2 CLIC1 GZMA PRF1 APOBEC3C RHOA CCL4 COTL1 PSME2 HLA-DPA1 HMGN2 LSP1 PSMB9 LCK SRP14 ARPC3 ARPC1B TPI1 APOBEC3G HLA-DPB1 LDHB ATP5G2 MYL12B PSMB8 PSMA7 HLA-DRB1 SUB1 ARPC4 CTSW SUMO2 TAP1 GZMB RARRES3 CAP1 UCP2 PPIB RAN CHCHD2 PARK7 HCST GABARAP HLA-DRA SOD1 CAPZB S100A4 RNASEK PPP1CA PKM IFI16 ACTR3 ITM2A SLC25A5 PGAM1 ANXA6 CD27 ATP5B LYST PSMB10 MIF LY6E ANKRD10 CTSD UBE2L6 EDF1 NONO TIGIT FKBP1A IL2RB HMGN1 ATP5L GZMH STAT1 GPI LCP2 GBP2 ARL6IP5 CCL4L1 PRDM1 OST4 PDCD1 HINT1 HNRNPF GBP5 COX7C ARPC5 GIMAP4 XRCC6 C17orf62 PRR13 HLA-DRB5 WDR1 ARL6IP1 ISG15 ATP5A1 EWSR1 COPE HAVCR2 EIF3H ANXA5 C11orf58 IFI6 SIRPG CALM3 SHISA5 DENND2D MAP4K1 BUB3 IKZF3 SNRPB EID1 PSMB1 PTPN6 NDUFA13 SSR4 COX8A PTPN7 MAT2B PSTPIP1 GSTP1 PSMB3 IRF9 TRAF3IP3 GIMAP7 PSMA2 SASH3 CD164 ETNK1 S100A11 KLRD1 MOB1A SH2D1A UBE2V1 SH3KBP1 ATP5G3 PSMA5 MT2A LAT IFNG RAB27A COX5A DDOST PSMB4 SRP9 BRK1 TNFRSF1B EIF4H GMFG ANXA2 TCEB2 RBPJ COX6A1 UBXN1 PSMD8 CD63 ATP6V0E1 NDUFB8 CTSC SNRPD2 ATP5C1 PRELID1 COX7A2 PSMA6 ECH1 U2AF1 HMGB2 FAM49B CD38 TSPO IDH2 CASP4 CCL3 TRMT112 SURF4 PSMA1 YWHAE LASP1 PYHIN1 ANAPC16 TUBB CSNK2B PRKAR1A SLAMF7 GNG5 PRDX1 RQCD1 CCNDBP1 INPP4B CDK2AP2 CBX3 RPN1 SPCS1 PSMA3 SIT1 XRCC5 EIF3C CXCR6 COX6C M6PR ANP32E |
